# Developing and validating the co-creation rainbow framework for intrinsic evaluation of methods: a health CASCADE structured review of models representing co-creation principles

**DOI:** 10.1186/s12961-025-01381-1

**Published:** 2025-10-10

**Authors:** Danielle Marie Agnello, Niamh Smith, Mira Vogelsang, Artur Steiner, Qingfan An, Janneke de Boer, Francesca Calo, Lea Delfmann, Danielle Hutcheon, Giuliana Raffaella Longworth, Quentin Loisel, Micaela Mazzei, Lauren McCaffrey, Jessica Renzella, Sebastien Chastin

**Affiliations:** 1 School of Health and Life Sciences, Glasgow Caledonian University, Glasgow, Scotland, UK; 2 Global Health Section, Department of Public Health Sciences, University of Copenhagen, Copenhagen, Denmark; 3 Department of Community Medicine and Rehabilitation, Umeå University, Umeå, Sweden; 4 Department of Public Health and Primary Care, Ghent University, Ghent, Belgium; 5 Department of Movement and Sports Sciences, Ghent University, Ghent, Belgium; 6 Faculty of Business and Law, The Open University, Milton Keynes, England, UK; 7 Yunus Centre, Glasgow Caledonian University, Glasgow, Scotland, UK; 8 Department of Sport Sciences, Faculty of Psychology Education and Sport Sciences Blanquerna, Ramon Llull University, Barcelona, Spain; 9 Nuffield Department of Primary Care Health Sciences, University of Oxford, Oxford, UK

**Keywords:** Co-Creation, Co-Production, Co-Design, Methods, System-Based methods, Participatory, Framework, Public health, Systems

## Abstract

**Background:**

The growing interest in co-creation for public health innovation highlights the need for systematic approaches to stakeholder engagement. Despite its potential, co-creation faces substantial challenges, including unresolved power dynamics, poor reporting of methods and the absence of a universally agreed-upon definition. Current research reveals substantial fragmentation in co-creation literature, with limited guidance on method selection and principle alignment. This study addresses these gaps by developing a framework for systematically evaluating method alignment with key co-creation principles, offering a structured approach to fostering more effective and adaptive collaborative processes.

**Methods:**

Using a structured review approach based on the Preferred Reporting Items for Systematic Reviews and Meta-Analyses guidelines, image-based models representing co-creation principles from academic and non-academic sources were identified and assessed. A framework was created through an iterative development process. The framework was subsequently validated by 12 geographically diverse co-creation researchers using a closed card sort method, ensuring its robustness and applicability across different research contexts.

**Results:**

The Co-Creation Rainbow Framework was developed by integrating key features from 20 included models, creating an individual-to-collective continuum with five sections (informing, understanding, stimulating, collaborating and collective decision-making), and three themes (engage, participate and empower). Successfully mapping 416 methods to the framework demonstrated its robust capability to differentiate and categorize co-creation methods, and reveaed nuanced variations in methodological strategies used by researchers and practitioners across different contexts.

**Conclusions:**

The Co-Creation Rainbow Framework addresses the disconnect between theoretical and practical co-creation approaches through operationalising co-creation principles. By challenging traditional linear models and acknowledging the diversity of co-creation methods, the framework provides a nuanced and adaptable tool for systematically evaluating method alignment. The framework offers researchers and practitioners a robust tool for meaningful collaborative innovation, ultimately opening new pathways for collective problem-solving and knowledge generation.

**Supplementary Information:**

The online version contains supplementary material available at 10.1186/s12961-025-01381-1.

## Introduction

There is a growing interest in applying co-creation as a process for innovation and stakeholder engagement in public health, as it has been shown to enhance the uptake of evidence-based interventions by engaging relevant stakeholders [1–3]. Co-creation integrates social determinants and contextual factors from the earliest stages of intervention design [1]. Unlike traditional approaches that merely merge different types of knowledge, co-creation actively engages diverse stakeholders who are impacted by or can influence a defined problem to co-create relevant solutions [4]. It emphasizes equitable collaboration, where power is shared, and each stakeholder’s insights are valued [5]. By doing so, co-creation transcends participation by a single group of actors and facilitates the meaningful generation and application of knowledge to achieve impactful outcomes [4, 6].

Despite its growing popularity, working with diverse stakeholders is not straightforward, and co-creation faces various challenges, such as the navigation of varied perspectives, miscommunication, the need for coordination and facilitation, unresolved power dynamics, inconsistencies in collaborative commitment throughout the process and substantial time and resources [5, 7–9]. Furthermore, the lack of consensus about co-creation, coupled with the interchangeable use of terms such as co-creation, co-design and co-production, contributes to fragmentation in the literature and hinders the development of a cohesive co-creation process [7]. Additionally, Agnello et al. [10] recently uncovered little to no consistency in the methods and combinations of methods used in co-creation, with a stark difference between methods sourced from academic literature from those found in grey literature. While academics leaned on qualitative methods, practitioners utilized more participatory and systems-based methods that enable collaboration [10]. This discrepancy highlights a significant divide in bridging academic and practitioner co-creation efforts, further complicating the implementation of cohesive and systematic co-creation practices.

Research methods are essential for data collection. In co-creation, however, methods not only gather data but also facilitate participant engagement and structure the research process throughout all phases, from conceptualisation to implementation, utilizing a range of approaches such as written, visual, observational and arts-based techniques [8, 10, 11]. For instance, system-based methods (for example, Causal Loop Diagrams or Group Model Building) offer a valuable means to harmonize and integrate different perspectives and visualize their interconnected and interdependent nature [9]. Grindell et al. [12] emphasized the importance of making principles visible through methods. By effectively employing methods, researchers can embody and operationalize their chosen methodology’s core principles, ensuring that the research process is rigorous and reflective of its methodological foundations. Therefore, successful adherence to co-creation principles relies on thoughtful consideration of how they are operationalized by methods used in the co-creation process.

Co-creation is inherently dynamic, interdisciplinary and context-dependent, requiring methods that can adapt to diverse settings and stakeholder groups [9]. Therefore, designing a co-creation project requires not only consideration of the project’s objectives but also how co-creators will work together in each step of the process, using various methods. The lack of guidance on how to select methods, leaves practitioners and researchers to navigate this complex terrain without clear tools. The fragmented nature of the literature and the lack of models or frameworks that integrate methods with principles further complicate the design and implementation of co-creation.

Existing frameworks, while valuable, are often limited by their focus on particular aspects of participation. As Grcheva and Vehbi argue, experts in participation are claiming that public involvement and its frameworks (such as IAP2 Spectrum and different participation ladder models) cannot work in various situations owing to haziness regarding decision-making processes [13].

To address these challenges, a framework for evaluating the alignment between methods and co-creation principles is needed. Such a framework can provide a structured approach to selecting, applying and reporting methods, ensuring that co-creation processes are adaptable, inclusive and effective across diverse contexts. Researchers have developed various models and frameworks for co-creation [14–17], including one for selecting participatory methods [11]. Previous research has also combined existing co-creation models to guide the overall process [16, 18–20]. However, despite the variety of models, there is no universally accepted framework, and none specifically focuses on the various methods used throughout co-creation, and whether they enact co-creation principles.

Therefore, this study is grounded in five key principles consistently recognized in the co-creation literature: empowerment, participation, collective creativity, collective intelligence and decision-making [1, 4, 16–18, 21–30]. These five principles were selected because they are repeatedly cited across the co-creation literature, represent conceptually distinct dimensions of collaborative practice, and are sufficiently operational to allow assessment at the level of method selection and use. By framing these as guiding principles derived from shared themes in the literature, this approach aligns with the recurring values that underpin successful co-creation processes.

### Aim

Building on prior theoretical and empirical work, this study seeks to address a gap in the co-creation literature by developing and validating a heuristic framework that supports intrinsic evaluation of methods [54], which analyses whether a method enacts some or all of the principles of co-creation.

In this way, the framework aims to serve as a practical framework that supports researchers and practitioners in moving from theory and practice, offering researchers and practitioners a robust tool for intentional and principle-informed method selection. This framework builds on existing models and co-creation methods to ensure relevance and practical applicability.

## Methods

### Working definitions

Co-creation can be used interchangeably with other terms, such as co-design and co-production, and can be confused with some participatory and public engagement methodologies [3]. Therefore, this study aligns with the research of Vargas et al. 2022, which treats co-creation, co-design and co-production as methodologically distinct yet related concepts [31].

The definitions used to inform this study, and newly established definitions, are the following:

- *Co-Creation*: Co-creation is defined as the active involvement of stakeholders, from the exploration and articulation of problems or needs to the creation, implementation and evaluation of solutions or initiatives [31].
- *Co-Creation Method*: Co-creation methods encompass a diverse range of tools, activities, approaches and techniques strategically employed across the entirety of the co-creation process. These methods serve various purposes, including but not limited to data collection, facilitation, recruitment, reflection, data analysis and dissemination, allowing for flexibility in achieving diverse objectives [10].
- *Framework*: A framework usually denotes a structure, overview, outline, system or plan consisting of various descriptive categories, for example, concepts, constructs or variables and the relations between them that are presumed to account for a phenomenon. Frameworks do not provide explanations; they only describe empirical phenomena by fitting them into a set of categories [32].
- *Model*: A model typically involves a deliberate simplification of a phenomenon or a specific aspect of a phenomenon. Models need not be completely accurate representations of reality to have value. Models are closely related to theory and the difference between a theory and a model is not always clear [32].
- *Participation*: Several definitions of participation are available; in this study, the definition by the Cambridge Dictionary was used: participation is ‘to take part in or become involved in an activity’ [33].
- *Empowerment*: Empowerment is the transfer of power from one entity (Agent A) to another (Agent B), enabling a shift from powerlessness to relative control, particularly in decision-making processes [22, 34].
- *Collective Decision-Making*: A new definition was adapted from Amorim and Ventura [35]. Collective decision-making is the coordination of the decision-making process, involving every co-creator in a manner that harmonizes with their common priorities and overarching objectives.
- *Collective Intelligence*: Collective intelligence is shared intelligence emerging from a group of people when they work on the same tasks, which could result in more innovative outcomes than when individuals work alone [36].
- *Collective Creativity*: Collective creativity occurs when group members stimulate one another’s divergent thinking, and their individual ideas are aggregated into the group’s creative output. Explanations for collective creativity are then based on how the group’s cognitions, dynamics and environments affect the creative process [37].

### Developing and validating the co-creation rainbow

#### The Co-Creation Rainbow Framework was developed using a two-phase approach:

*Phase 1*: Framework Development. This phase included two steps: (1) a structured review of visual models to identify and evaluate image-based models of co-creation principles (empowerment, participation, collective creativity, collective intelligence and decision-making) from both academic and non-academic sources; and 2) multiple rounds of testing and consensus-building were conducted with four co-creation researchers, and tested on a set of co-creation methods using the closed card sort method to develop the initial framework.

*Phase 2*: Validating the Framework. This phase validated and refined the framework created in Phase 1. It consisted of five steps: (1) identification, (2) criteria, (3) closed card sort method, (4) analysis and (5) summary.

This two-phased approach ensures that the framework is both theoretically grounded and empirically tested, enhancing its reliability and applicability.

### Phase 1: Framework development

The framework was developed through a two-step approach: Step 1, systematically identified relevant visual models for these principles, and Step 2, involved iterative testing and consensus-building to integrate key elements from the included models into a hybrid framework. This process ensured a comprehensive and inclusive synthesis of co-creation principles across diverse contexts.

#### Step 1: Screening and testing existing models

A structured review approach was used, which involves applying objective search criteria and systematically screening and extracting relevant information from each source to ensure transparency, repeatability and comprehensiveness. This approach also allows flexibility in review design when standard systematic review protocols are not feasible [38].

This structured review is reported following the Preferred Reporting Items for Systematic Reviews and Meta-Analyses (PRISMA) checklist [39] and is designed on the basis of the approaches of de Koning et al. [18] and Payne et al. [20]. Specifically, we applied de Koning et al.’s search and screening strategy and drew on Payne et al.’s framework development process, adapting it to fit the focus and scope of our study.

Inspiration was drawn from Koning et al., who took a similar visual approach in their investigation of models of co-creation. Their work demonstrated the value of synthesising visual frameworks to map conceptual structures across different contexts [18].

A systematic search was conducted in Google Images to identify visual models related to key co-creation principles. Search terms were combined using ‘AND’ to refine results. For example, ‘co-creation’ was paired with ‘decision-making’ and ‘methods’ to narrow down broad terms. However, for ‘participation’ and ‘empowerment’, co-creation was not added, as these terms are inherently linked to co-creation processes. Similarly, ‘collective intelligence’ and ‘collective creativity’ were specific enough to stand alone. Table 1 shows the search term combinations used.

**Table 1:** Search term combinations for the Google Images search

Search term 1	Search term 2	Search term 3
Model	Participation	
Framework	Participation	
Model	Empowerment	
Framework	Empowerment	
Model	Collective intelligence	
Framework	Collective intelligence	
Model	Collective creativity	
Framework	Collective creativity	
Model	Decision-making	Co-creation
Framework	Decision-making	Co-creation
Model	Methods	Co-creation
Framework	Methods	Co-creation

Owing to the high number of search results for the Google Images search, when repetition was apparent at around 100 images, with almost no new relevant images found, this served as the stopping point. Once the search was conducted, the models were screened with the selection criteria 1 in Table 2.

**Table 2:** Selection criteria 1

Inclusion criteria	Exclusion criteria
Contains the name of one or more co-creation principles	Does not contain any of the co-creation principles
It is clear and concise in both language and image quality	It is unclear and difficult to understand on the basis of the image
It is not specific to a certain field, and can be used in different sectors or methodologies	It is designed for a specific field of research
It is written in English	It is not written in English

Models adhering to the inclusion criteria were downloaded and then screened for a second time using selection criteria 2 in Table 3. Models were not screened or excluded on the basis of the presence of the term ‘co-creation.’ Instead, inclusion was determined by whether a model aligned with one or more of the five co-creation principles of interest. The aim was to determine their applicability in evaluating methods employed in specific steps of the co-creation process, rather than the entire co-creation project. Models meeting these criteria were taken to Step 2.

**Table 3:** Selection criteria 2

Inclusion criteria	Exclusion criteria
Provided example methods	Did not provide example methods
Includes a clear and sufficient description of the model’s structure, components or intended use, and demonstrates alignment with the intended co-creation principle as defined in this study	Lacks a clear or sufficient description of the model’s structure, components or intended use, or does not demonstrate alignment with the intended co-creation principle as defined in this study
It is written in English	It is not written in English

To ensure quality and consistency throughout the screening process, all included models were reviewed by the lead author (D.M.A.) and co-authors (N.S., M.V. and A.S.). Discrepancies were discussed and resolved through consensus.

#### Step 2: hybrid framework development

To create the framework, an adapted version of the framework development approach described by Payne et al. [20] was applied, starting with creating an initial version of the framework that was progressively refined through iterative testing and consensus-building.

The lead author (D.M.A.) and co-authors (N.S., M.V. and A.S.) assessed the identified models by testing whether their structure could meaningfully accommodate co-creation methods. This involved mapping a sample of methods onto the model using the close card sort method to evaluate their flexibility and fit. Models or model features that successfully allowed for the categorization of multiple methods were considered suitable for inclusion.

The synthesis process involved iterative rounds of consensus-building. First, the lead author (D.M.A.) and co-authors (N.S., M.V. and A.S.) independently evaluated the models. This was followed by group discussions to clarify assumptions and findings, and finally, a collaborative meeting to agree on the final structure. The resulting framework, named the Co-Creation Rainbow Framework, was advanced to the validation stage.

### Phase 2: validating the framework

To validate the Co-Creation Rainbow Framework, a comparative analysis approach was applied to evaluate its accuracy and utility in differentiating co-creation methods. The analysis followed five steps, summarized in Table 4.

**Table 4:** Steps of the comparative analysis

Step	Description
1. Identification	The Health CASCADE Co-Creation Methods Inventory [10, 40] was selected as the dataset for method comparison, given its comprehensive collection of co-creation methods. The inventory includes a diverse set of participatory, qualitative, quantitative and mixed methods sourced from both academic and grey literature
2. Criteria	The five sections of the Co-Creation Rainbow Framework (informing, understanding, stimulating, collaborating and collective decision-making) served as the criteria for comparison of the different methods. The sections are described in the Results section and Fig. 3
3. Closed Card Sort method [41]	Twelve co-creation researchers (D.M.A., D.H., F.C., G.R.L., J.B., L.D., L.Mc.C., M.M., M.V., N.S., Q.A. and Q.L.), diverse in experience with co-creation, field of research, and varying geographical locations, participated in a closed card sorting task using the MAZE platform [42]. The closed card sort method enabled us to understand how people understand and categorize methods, and ensures the framework matches users’ expectations. This process assessed the alignment of methods with the framework’s categories
4. Analysis	The agreement rates for method categorization were calculated, and a comparison was made between methods sourced from academic versus grey literature
5. Summary	Researchers reflected on the findings and provided feedback on the framework’s strengths and areas for improvement. These insights were used to refine the framework and summarize the study’s results and are presented in Additional File 5

#### Data analysis

Upon completion of the closed card sort, the agreement rates were calculated for each method and its assigned section. In alignment with the close card sort protocol, methods with an agreement rate of 50% or higher were allocated to a section of the Co-Creation Rainbow Framework. Methods with agreement rates below 50% were classified as unsorted. The outcomes were compiled into a report and shared with the research team for further reflection and discussion.

To validate the framework, an overall agreement rate was calculated for the sorted methods. A 100% agreement rate indicated unanimous categorization by all 12 researchers, while a 50% agreement rate was the threshold for satisfactory performance. Agreement rates for each section of the framework were organized into 10% increments and visualized using a histogram.

Additionally, methods sourced from academia were compared with methods sourced from grey literature to uncover trends in the degree of alignment by source type. Sorted methods were categorized by source type, and the percentage distribution of academic and grey literature methods was calculated for each section of the framework.

## Results

### Phase 1: framework development

#### Step 1: screening and testing existing models

**Fig. 1:**
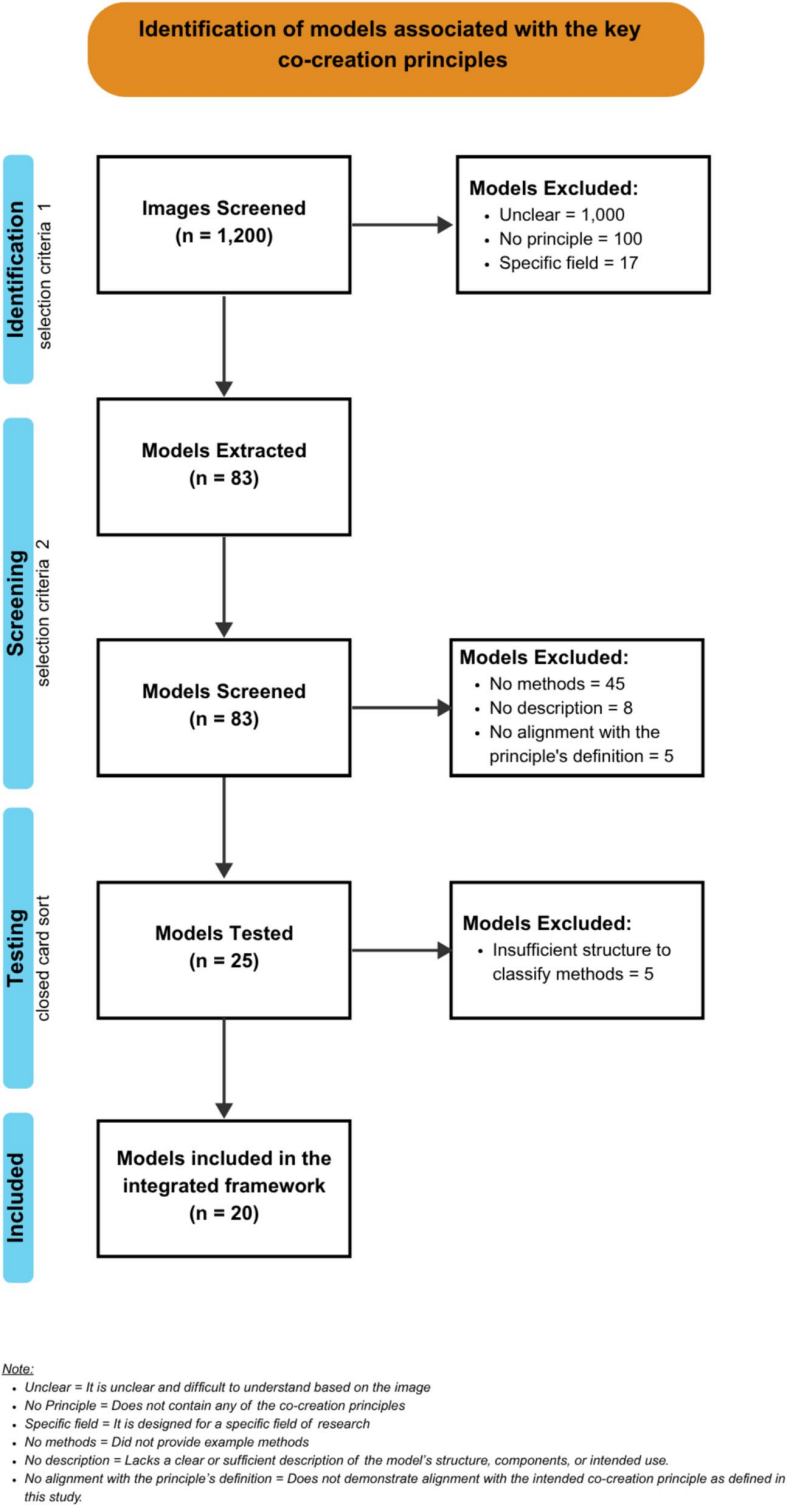
PRISMA-like flow chart of models and frameworks that visualizes the steps from identification to the inclusion of the final set of 20 models associated with the co-creation principles. Definitions for the exclusion criteria used at each step are provided at the bottom of the figure

**Fig. 2:**
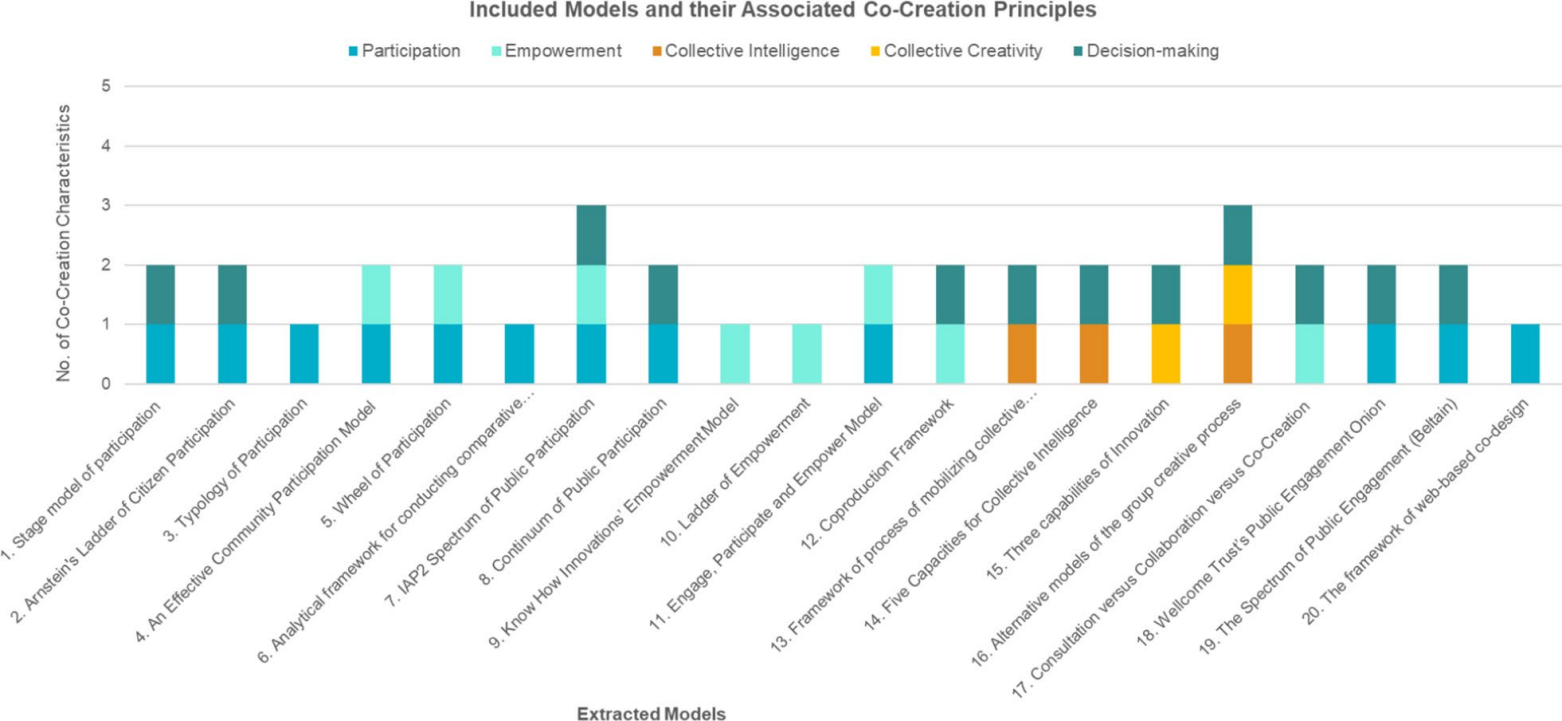
Included models and their associated co-creation principles. The graph represented the distribution of the co-creation principles among the 20 included models

From an initial 1200 hits, 1117 models were excluded on the basis of selection criteria 1, leaving 83 models for inclusion. Of these 83 models, 25 met selection criteria 2 and proceeded to the testing phase. An additional file provides detailed information on these models, including their sources, descriptions and co-creation principles (see Additional File 1). Testing the models revealed that five did not offer sufficient structure or guidance to support the classification of methods and were therefore excluded. Figure 1 shows the process of model inclusion, resembling a PRISMA-like flow chart. Figure 2 illustrates the included models and their associated principles, and an additional file contains the completed PRISMA checklist (see Additional File 2).

Of the 20 models included in the study, 60% (12/20) feature participation, 60% (12/20) include decision-making, 40% (8/20) address empowerment, 15% (3/20) involve collective creativity and 10% (2/20) incorporate collective intelligence.

#### Step 2: hybrid framework development

These 20 included models were reviewed through four iterative rounds of testing and consensus-building among the authors to determine which structural features could accommodate the categorization of co-creation methods. This process informed the integration of key elements into a hybrid framework, ensuring that the final structure reflected both conceptual alignment and practical applicability to method selection. An additional file contains details about which features of the source models were added to the hybrid framework (see Additional File 3). An additional file contains further details on the results of Phase 1 (see Additional File 4).

#### The co-creation rainbow framework

**Fig. 3:**
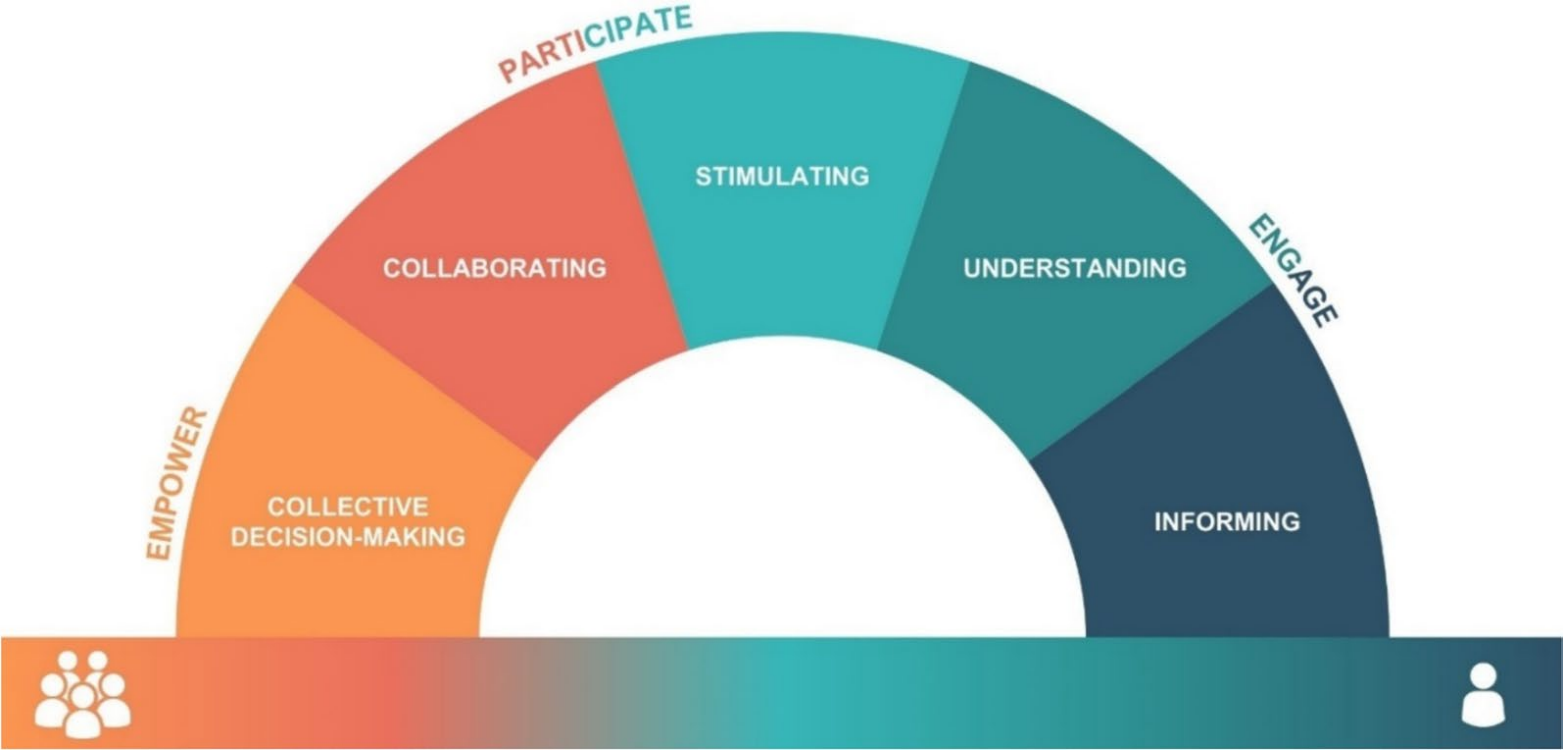
The Co-Creation Rainbow Framework, including the individual-to-collective continuum across the bottom of the figure; five sections that form the arc of the rainbow (informing, understanding, stimulating, collaborating and collective decision-making), and three themes (engage, participate and empower) that span the five sections

The Co-Creation Rainbow Framework integrates the strengths and features of 20 distinct models to form a tool for evaluating methods, resulting in an individual-to-collective continuum, five sections (informing, understanding, stimulating, collaborating and collective decision-making) and three themes (engage, participate and empower), which is visualized in Fig. 3.

The name ‘Co-Creation Rainbow’ was chosen to reflect the framework’s role in examining the spectrum of methods and to symbolize the diverse range of co-creation principles it encompasses. The term ‘rainbow’ signifies the diverse principles and positions for methods within the framework, serving as a visual metaphor that underscores that no single point within the framework holds more significance than another. Instead, the framework is designed to discern how a particular method contributes to the spectrum of co-creation principles. This name highlights the concept that co-creation can manifest in diverse forms and intensities; the framework provides a systematic approach to understanding and assessing methods used in the co-creation process.

#### Individual-to-collective continuum

Co-creation is not an individual achievement, but a cumulative work of a collective variety of individuals, using the dimension of creativity in their work [43]. While dimensions of individual and collective creativity are distinct, they are also interconnected and must be considered together [44]. Therefore, along the bottom of the Co-Creation Rainbow Framework is a continuum from individual to collective, reflecting integrated features of collective intelligence and collective creativity. This continuum emphasizes how some methods only involve the co-creators on an individual basis, while others enable the participation of the collective. The continuum is intended to illustrate the type of engagement methods that can foster, with informing methods often targeting individual understanding, while later stages, such as collaborating and collective decision-making, facilitate more explicitly collective forms of intelligence input. This continuum is visualized across the bottom of the framework in Fig. 3.

#### Framework sections

An integral aspect of the Co-Creation Rainbow Framework is its arch structure, formed by five sections spanning the individual-to-collective continuum. These sections mirror the order of some of the included models, such as Duarte et al. (2018) Stage Model of Participation, the Wellcome Trust’s Public Engagement Onion [46, 47], the Spectrum of Public Engagement and the IAP2 Spectrum of Public Participation [48, 49]. Each section within the framework holds a distinct position on the individual-to-collective continuum, aligning with specific objectives users aim to accomplish through their selected methods. To illustrate the framework’s sections, Table 5 presents some examples from the literature of where methods fit in the features of some related models.

**Table 5:** Sections of the co-creation rainbow framework

Section	Description	Examples
Informing	A method to provide information to the co-creators or inform them of the key aspects of the co-creation process	Fact sheets, websites, public lectures and leaflets.^1^
Understanding	A method aimed at comprehending the co-creator’s experience: involves actively or passively gathering knowledge, data or feedback from the co-creator	Focus groups, surveys and interactive exhibits.^2^
Stimulating	A method to stimulate the co-creator’s individual or collective intelligence and creativity, or energize them, thereby stimulating the co-creation process	Hackathon^3^
Collaborating	A method that facilitates collaborative engagement among co-creators, fostering collective intelligence and creativity. It provides a platform for elaborating on existing ideas or generating new ones. Co-creators with diverse expertize and perspectives engage in collaborative activities, excluding involvement in decision-making	Design games and workshops.^4^
Decision-making	A method designed to involve co-creators in decision-making. Co-creators collectively influence decisions to define the direction of the co-creation process or take specific actions	Consensus-building or participatory decision-making methods, citizen juries and ballots.^5^
^1^[47–49] ^2^[46–51] ^3^[52] ^4^[46, 48, 50, 51, 53] ^5^[48, 49]		

#### Overlayed themes

To underscore the progression from engaging to participating, and then to empowerment in co-creation, introduced by an included model by Steiner and Farmer (2018), the Co-Creation Rainbow Framework includes three themes that overlap with the five sections of the framework: engage, participate and empower. Descriptive details and associated sections are presented in Table 6.

**Table 6:** Engage, participate and empower themes of the co-creation rainbow framework

Themes	Description	Associated section(s)
Engage	Co-creators identify potential co-creation opportunities and relevant stakeholders. They’re involved through various communication methods, laying the groundwork for co-creation. They also provide their individual knowledge, experiences and perspectives to enrich the co-creation process	Informing and understanding
Participate	Co-creators actively contribute to the co-creation process, collaborating through iterative dialogue and fostering in-depth working relationships to develop solutions	Stimulating and collaborating
Empower	Co-creators are empowered to participate in decision-making processes, enabling them to achieve their personal and community goals	Collective decision-making

The strategic positioning of each theme with respect to a section highlights the sequential stages of transferring power or agency from organizers to the co-creators during the co-creation sessions. As articulated by Grove [54], empowerment extends beyond mere participation but encompasses the ability to take part in decision-making processes and affect the outcomes.

### Phase 2: validating the framework

**Fig. 4:**
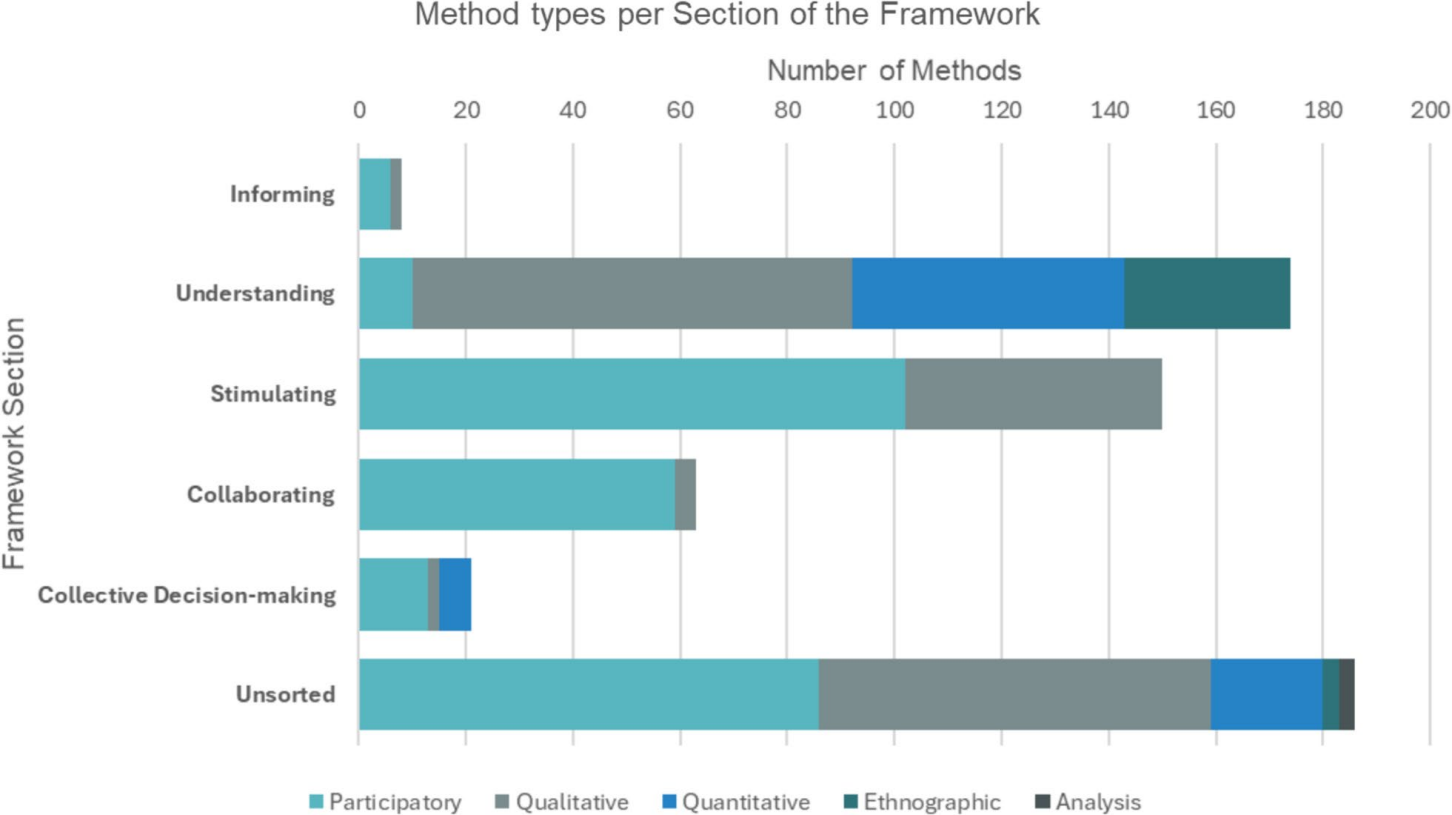
Number and type of methods sorted to each of the five sections of the Co-Creation Rainbow Framework (informing, understanding, stimulating, collaborating and collective decision-making), and those that were unsorted

**Fig. 5:**
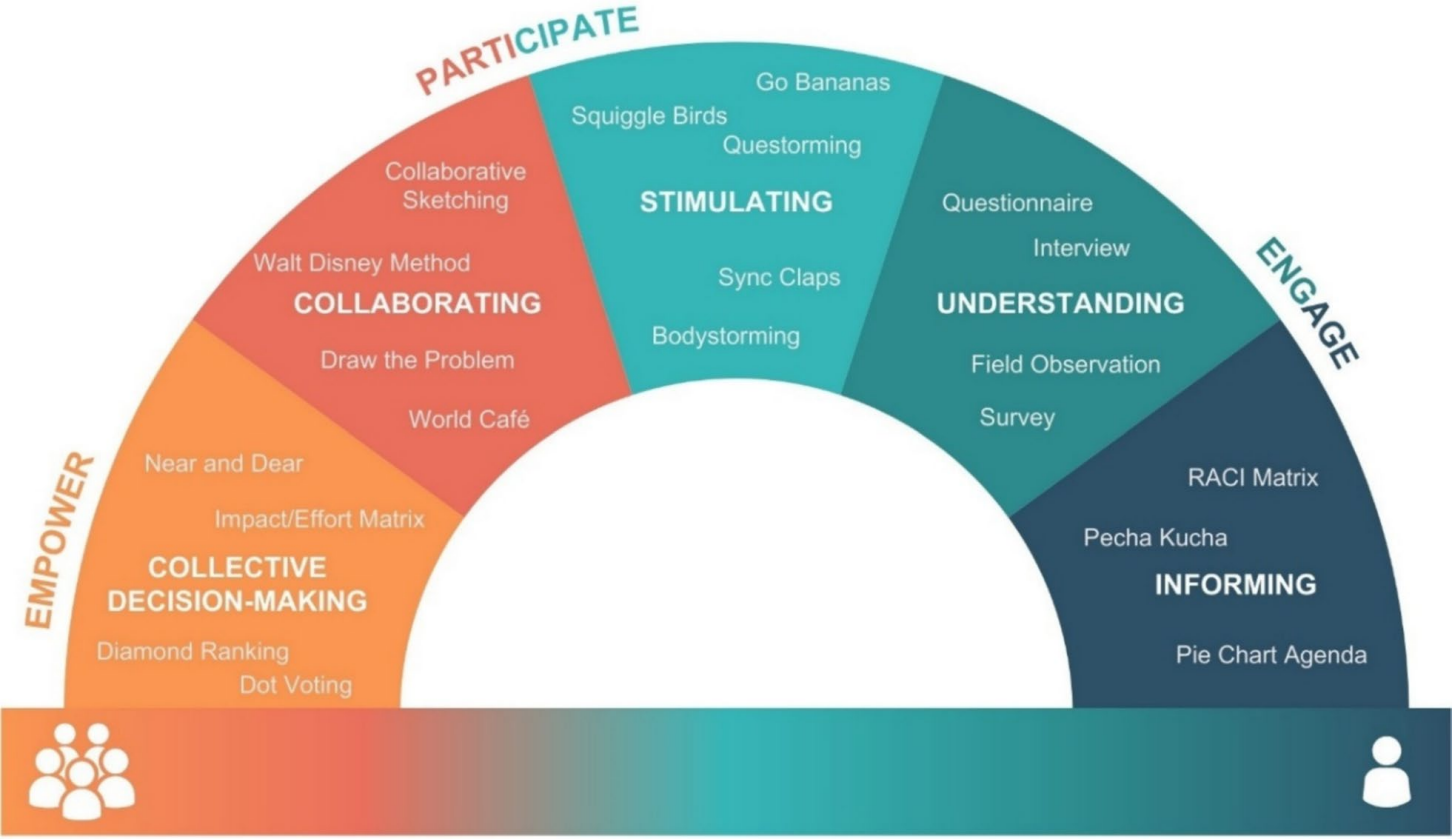
Visualizes only a few illustrative examples of methods sorted into the Co-Creation Rainbow Framework, not the full validation set (see Additional File 5). Presented: informing (*n* = 3), understanding (*n* = 4), stimulating (*n* = 5), collaborating (*n* = 4) and collective decision-making (*n* = 4)

Out of 619 methods sorted to the framework using the closed card sort method, 67% (*n* = 416) were successfully sorted into the five sections of the Co-Creation Rainbow Framework: informing section (*n* = 8), understanding (*n* = 174), stimulating (*n* = 150), collaborating (*n* = 63) and collective decision-making (*n* = 21). Conversely, 33% (*n* = 203) of the methods did not meet the agreement threshold to be mapped to the framework. An additional file contains a detailed list of methods sorted into the framework (see Additional File 5), Fig. 4 visualizes the method types per section of the framework, and Fig. 5 visualizes a few illustrative examples of methods sorted into the framework.

**Fig. 6:**
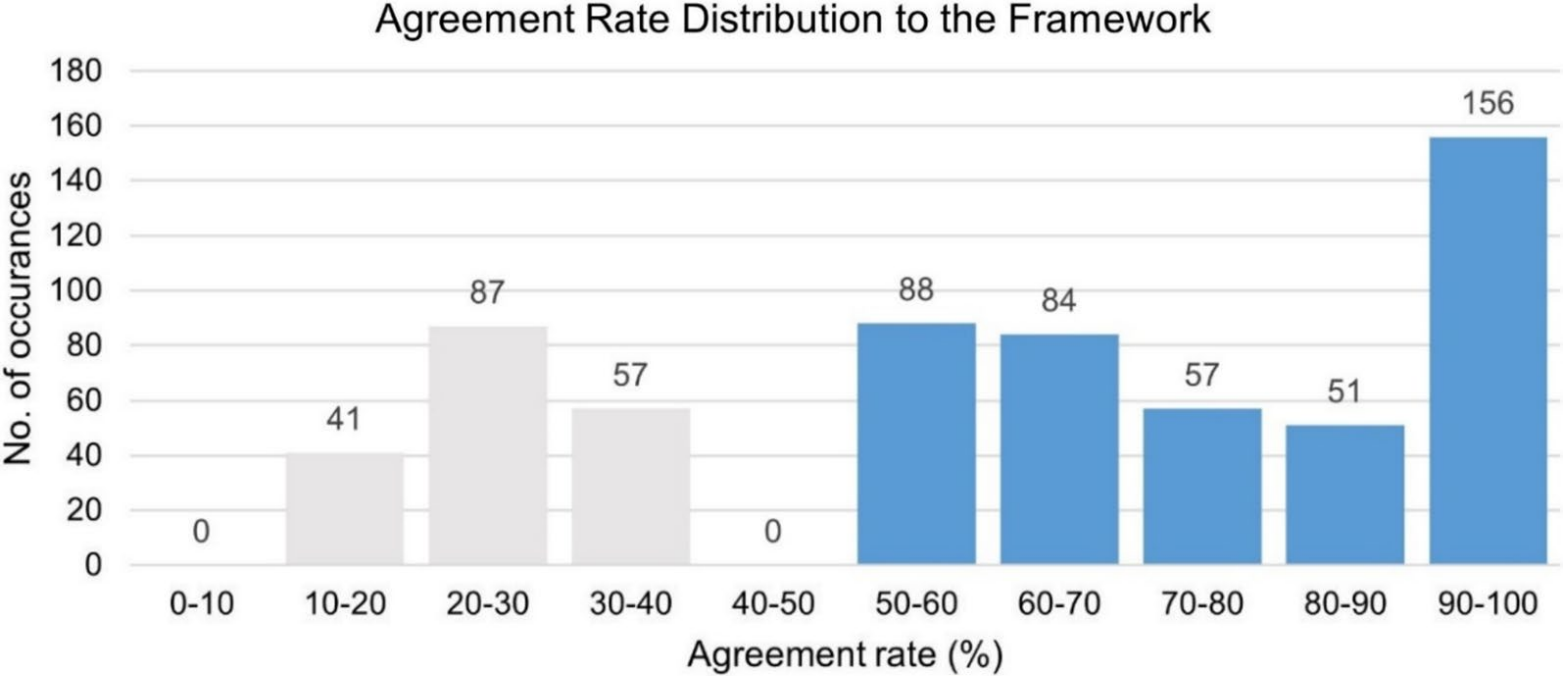
Agreement rate distribution from 0 to 100%. The agreement rates are divided into 10% brackets. The occurrence is the method plus its assigned section, for example, a decision made by the researcher taking part in the sorting method

Figure 6 visualizes the distribution of overall agreement rates for the Co-Creation Rainbow Framework. As noted, 70% of methods had agreement rates of 50% or higher, with a significant concentration in the 90–100% range, showcasing a high level of consensus.

#### Comparison of methods

**Fig. 7:**
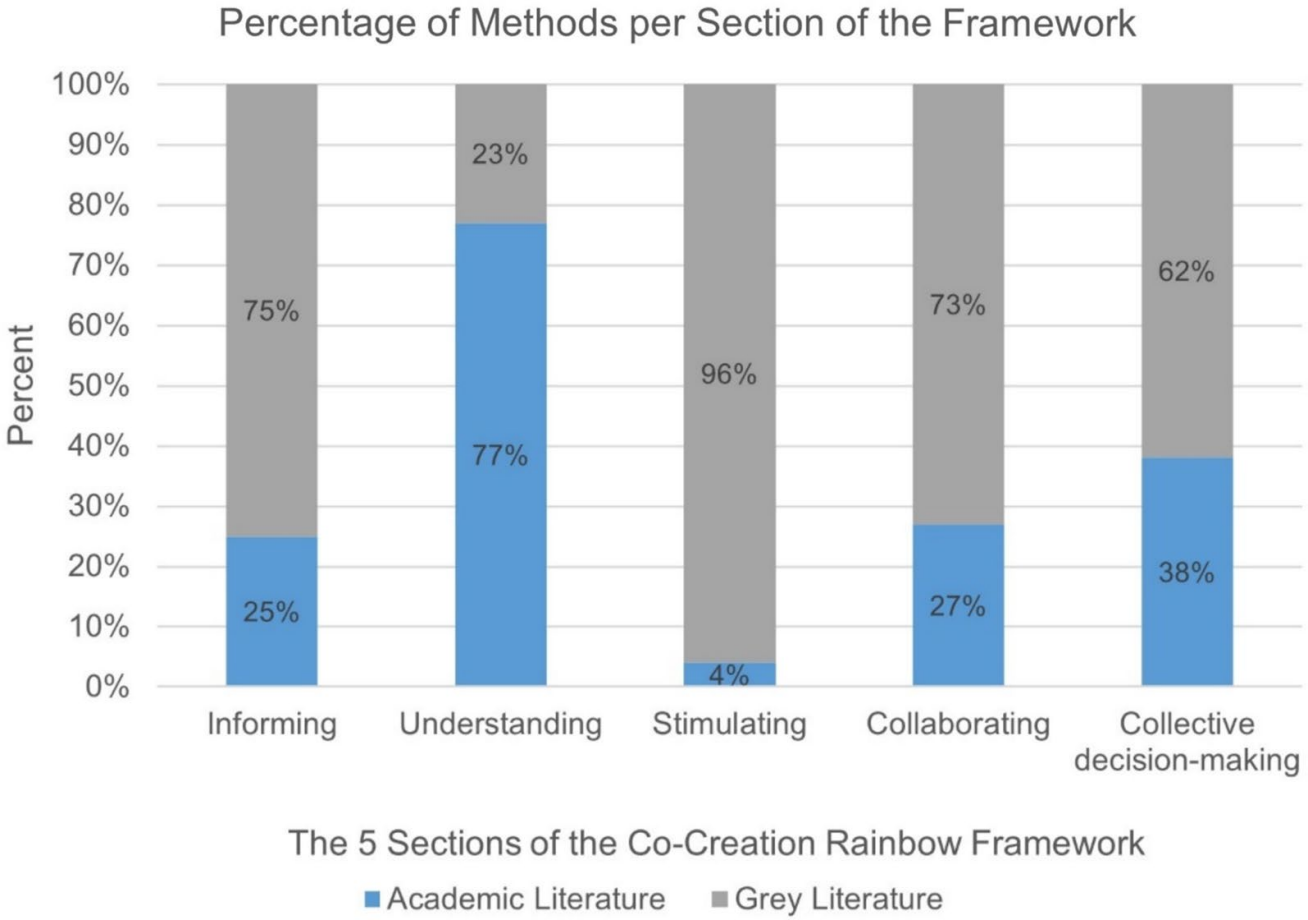
Percentage of methods sourced from either academic or grey literature. The Co-Creation Rainbow Framework sections are on the x-axis, and the percentage is on the y-axis

Figure 7 illustrates the proportion of sorted methods based on their source literature. It shows that methods from academic literature predominantly focus on understanding co-creators (77% in the understanding section), often drawing on individual knowledge and thus limiting their capacity to address other co-creation principles such as collective intelligence, collective creativity, decision-making or empowerment. Notably, only 4% of methods in the academic literature are categorized under the stimulating section, indicating a low emphasis on stimulating the co-creation process. In contrast, methods from grey literature were primarily sorted into the stimulating (96%), informing (75%) and collaborating (73%) sections. This suggests that methods used by practitioners (in the grey literature) more actively enact principles such as participation, collective intelligence and collective creativity, with less emphasis on empowerment and collective decision-making (62%) and on individual experience or knowledge (23%).

## Discussion

The framework created in this study synthesizes key elements identified across diverse models to guide an assessment of the extent to which methods adhere to co-creation principles. The framework supports intrinsic evaluation of methods [55], which is analysing whether a method is designed to enact some or all of the principles of co-creation. This type of evaluation does not assess outcomes such as behaviour change or health impact but instead provides practical guidance for selecting methods that are intrinsically aligned with the values and goals of co-creation initiatives. Moreover, the Co-Creation Rainbow Framework introduces a structured language for articulating how methods contribute to the overall process, fostering transparency about co-creative endeavours. In this way, the framework provides an internal consistency check for co-creation methods and examines how well methods align with co-creation. This framework is therefore a valuable addition to part of a broader evaluation of co-creation [1].

### Method selection

Although the five co-creation principles informed the selection of models in the synthesis phase, the subsequent sorting of individual methods was based on the five sections of the Co-Creation Rainbow Framework. This reflects the purpose of a framework, which, as defined by Nilsen [32], serves to organize empirical phenomena into structured categories without offering explanatory claims. In this case, the framework categorizes methods according to their primary function within the co-creation process, providing researchers with a heuristic structure to support transparent and intentional method selection. The expectation is that by employing methods across all five sections of the framework, researchers can operationalize and uphold all the principles of co-creation throughout the full co-creation process.

While co-creation is often situated within qualitative or collaborative research traditions that emphasize participation and shared decision-making, principles are not always explicitly operationalized in practice. As noted by Messiha et al. (2023), there remains an inconsistency in the articulation of co-creation theories and how they shape methodological choices in co-creation [56]. This limits the ability to assess or compare co-creation efforts and may contribute to a fragmented evidence base lacking coherence and evaluative rigour [3].

The Co-Creation Rainbow Framework seeks to address this gap by providing a practical tool that helps researchers evaluate whether and how specific methods align with commonly accepted co-creation principles. By offering a structured lens for assessing method-principle fit, the framework supports more transparent, deliberate design choices and contributes to improving the comparability, quality and inclusivity of co-creation research.

This tool may be particularly valuable in applied, interdisciplinary or policy-driven contexts where co-creation is increasingly adopted but often lacks concrete guidance on implementation. It may also support experienced co-creation researchers in reflecting more critically on the alignment between their methodological choices and their objectives.

While the Co-Creation Rainbow Framework supports the selection of methods on the basis of the method’s intrinsic characteristics, it remains essential to consider cultural appropriateness and contextual fit, as additional factors can influence a method’s success. The framework is intended to guide the initial planning and selection phase, but piloting and adapting methods to specific co-creation processes is still necessary to ensure effective implementation.

### Individual to collective

The individual-to-collective continuum of the framework captures two co-creation principles: collective intelligence and collective creativity. Co-creation blends ideas, perspectives and experiences, leveraging crowd dynamics to unlock collective intelligence [57, 58]. In 2015, Woolley et al. [59] highlighted that collective intelligence is an emergent property that arises from both the aggregation of the group members ’ values, as well as group structures, norms and routines, which regulate group behaviour in a way that enhances coordination and collaboration. They emphasize that effective group performance relies on social perceptiveness, balanced speaking turns, diversity and creative methods. Similarly, Skrazauskiene and Kalinauskas [44] argue that collective creativity fosters collective intelligence.

To achieve collective intelligence, co-creators must collaborate in a supportive environment that promotes cohesion and leverages diverse viewpoints [17, 23]. The increased engagement while performing a collective creative task accumulates knowledge more efficiently and improves the development of solutions, whilst successful innovation requires effective and creative work [44]. For instance, Bojovic et al. [52] highlighted how workshops can actively engage co-creators in knowledge exchange, challenge them to confront opinions, build consensus and find common solutions for potentially conflicting interests and views. Thus, collective intelligence and creativity are markedly influenced by the methods used in the co-creation process (for example, in a workshop).

Nevertheless, relying solely on collective intelligence and creativity has limitations. Bojovic et al. [52] suggest that when seeking insight into stakeholders’ needs, perceptions and rationales, a more meaningful exchange can be achieved through one-on-one interviews. Interviews encourage co-creators to express themselves freely, facilitating the emergence of new narratives. For instance, Miller [60] warns of groupthink, a phenomenon where individuals conform to group opinions at the expense of their own, underscoring the need for individual investigation before aggregating views. He argues that collective intelligence only arises when we first individually investigate the situation with our own set of data, and from the lens of our own unique experience and understanding, and then comes together to aggregate our collective view [60]. This highlights the importance of selecting methods along the full individual-to-collective continuum, allowing co-creators to first work individually and then engage collectively. This is reflected in the design of the Co-Creation Rainbow Framework, with no position valued more than another. While some methods engage individuals rather than groups, they can play an essential role in a broader co-creation process when thoughtfully sequenced and integrated to support collective outcomes.

### Engaging to empowering

The overlayed themes of the Co-Creation Rainbow Framework (engage, participate and empower) capture three co-creation principles: participation, empowerment and decision-making.

The Co-Creation Rainbow aligns with Weidenstedt [34] in recognizing that power transfer within co-creation often necessitates social and structural changes. Effective empowerment requires co-creators to recognize the variability in power distribution and assess their positions and agential options. By aligning with Weidenstedt’s insights, the Co-Creation Rainbow Framework emphasizes the dynamic nature of power transfer, considering both social dynamics and the need for structural adjustments. In this way, the framework prompts critical reflection on the distribution of power throughout the co-creation process.

Consequently, the significance of visualizing the progression from Engage to Empower is crucial, as different methods facilitate varying degrees of power transfer. Steiner and Farmer [22] describe this as a gradual process, starting with engagement and moving through participation, both of which are prerequisites for empowerment. They note that ‘engagement is a precondition of empowerment, and it is insufficient, alone, to empower communities’ (p.129) [22]. For instance, Arnstein’s Ladder [61] emphasizes the redistribution of power, with its eight rungs ranging from manipulation to citizen control. While foundational, Arnstein’s ladder risks presenting co-creation as one-dimensional, implying that higher participation alone is sufficient. Unlike earlier models, which often conceptualize public engagement as a linear progression of power or influence, the Co-Creation Rainbow Framework takes on a hybrid approach. It extends beyond empowerment, incorporating participation processes, creativity and collective intelligence, key elements critical for fostering innovation and inclusivity in co-creation. Flexibility is necessary to adjust engagement levels based on the circumstances at different stages of the co-creation process. Therefore, the objective is not to relentlessly pursue high participation levels, as this may not be appropriate in every situation [45].

### Academic and grey literature methods

Through the application of the Co-Creation Rainbow Framework, our study further refined the findings of Agnello et al. [10] by articulating the nature of the gap in how researchers and practitioners engage in co-creation, particularly in terms of the methods they employ. Academic researchers tend to utilize collaborative methods less frequently compared with their counterparts outside academia. Conversely, practitioners and individuals outside academia exhibit a higher propensity for employing stimulating and collaborative methods. Intriguingly, neither academic researchers nor practitioners are creating platforms for co-creators to engage in collective decision-making. This may be because academic co-creation processes often emphasize data extraction over shared control, while practitioners and community-based actors may prioritize relationship-building and creativity. Notably, the limited use of collective decision-making methods across both domains highlights a persistent challenge in operationalising power-sharing. This underscores the importance of using the Co-Creation Rainbow Framework not only as a tool for method selection and reporting but also as a prompt for critical reflection on where and how power is distributed throughout the co-creation processes. Moving forward, this framework stands as a valuable resource, guiding researchers and practitioners alike towards more co-creative approaches. It provides them with a tool to more easily select methods from across the Co-Creation Rainbow Framework.

### Future research

Approximately 33% of the methods identified in this study could not be sorted into the framework. These included cross-cutting participatory tools, individual reflective techniques, data analysis methods and observational or sampling strategies and analysis methods. A dedicated classification of these methods could support researchers and practitioners in identifying supportive or adjunct methods that enhance co-creation processes, particularly those that operate across multiple phases or outside of direct engagement contexts. Furthermore, future research could explore the development of a complementary framework or typology to accommodate methods that fell outside the scope of the Co-Creation Rainbow Framework.

### Limitations

There are known limits to the card sort method regarding consistency between participants. This lack of consistency can weaken results and meaning [41]. Additionally, there is a potential for bias in the study due to possible subjective interpretation by the researcher sorting each method. For instance, while most methods were successfully categorized within the framework, 33% remained unsorted. This may be owing to inconsistencies in the level of detail provided in the source descriptions. This reflects limitations in the dataset rather than in the study design. However, to mitigate these risks, an optimal number of card-sorting participants were recruited, clear instructions and definitions were provided and methods with an agreement level below 50% were excluded. Importantly, the framework’s development through a geographically diverse and interdisciplinary lens ensures its relevance across different contexts, sectors and cultural landscapes.

While this study focused on identifying models aligned with co-creation principles, we acknowledge the conceptual overlap and shared foundations across adjacent traditions such as co-production, co-design and public involvement. Importantly, models were not excluded based solely on terminology; rather, inclusion was determined by whether a model reflected one or more of the five co-creation principles. Nonetheless, some relevant models may not have appeared in our search if they did not use overlapping keywords or were not visually documented in the available sources. Furthermore, this study did not assess the intended use or application context of each source model, which may influence the types of methods they recommend.

## Conclusions

The Co-Creation Rainbow Framework was developed for actualising co-creation principles through methods, which is an essential step in advancing innovation and leveraging the full potential of co-creation to address complex and wicked problems. While existing research has often focused on the theoretical aspects (the ‘what’), this study uniquely addresses the practical application (the ‘how’) by concentrating on methods used in co-creation processes. By operationalising co-creation principles, the framework aims to bridge the gap between theoretical models and practical application, inviting researchers and practitioners to embrace the complexity, diversity and dynamic nature of co-creative processes systematically and robustly.

The Co-Creation Rainbow Framework represents more than a methodological tool, it offers a new lens through which to conceptualize collaboration. By placing methods at the centre of co-creation and providing a nuanced, adaptable approach, the framework moves beyond linear models of participation and instead reflects the holistic and multidimensional nature of co-creation. The Co-Creation Rainbow Framework emphasizes that no single method or approach can capture the full spectrum of co-creation, and that method selection needs to be guided by intended principles and the purpose of engagement.

Importantly, using this framework does not guarantee meaningful co-creation outcomes. As with all collaborative approaches, the quality of facilitation, group dynamics, context and interpersonal skills remain equally important for success. A poorly facilitated method can result in tokenism or groupthink regardless of its conceptual alignment with co-creation principles. This framework is therefore best understood as a support structure for design, reflection, reporting and evaluation, which can help researchers and practitioners make more transparent, thoughtful decisions about method selection.

As co-creation continues to emerge as a critical approach to solving complex societal and organizational challenges in public health, the Co-Creation Rainbow Framework offers a timely and adaptable tool for intrinsically evaluating methods. It strengthens both academic understanding and practice by equipping users with a flexible yet structured guide to support principle-aligned co-creation.

## Supplementary Information

Additional file 1. Methods-specific models

Additional file 2. PRISMA Checklist

Additional file 3. Source models and features

Additional file 4. Detailed Results on Phase 1

Additional file 5. The co-Creation Rainbow Framework: card sorting outcome report

## Data Availability

All data generated or analysed during this study are included in this published article and its supplementary information files.

## References

[1] Longworth GR, Goh K, Agnello DM, Messiha K, Beeckman M, Zapata-Restrepo JR, et al. A review of implementation and evaluation frameworks for public health interventions to inform co-creation: a Health CASCADE study. Health Res Policy Syst. 2024;22:39. 38549162 10.1186/s12961-024-01126-6 PMC10976753

[2] Bauman A. A personal reflection on co-creation in public health: a dream partly realised. Public Health Res Pract. 2022;32:3222210. 35702743 10.17061/phrp3222210

[3] Agnello DM, Loisel QEA, An Q, Balaskas G, Chrifou R, Dall P, et al. Establishing a health CASCADE–curated open-access database to consolidate knowledge about co-creation: novel artificial intelligence-assisted methodology based on systematic reviews. J Med Internet Res. 2023;25: e45059. 37463024 10.2196/45059 PMC10394503

[4] Kint O, Duppen D, Vandermeersche G, Smetcoren A-S, De Donder L. How ‘co’ can you go? A qualitative inquiry on the key principles of co-creative research and their enactment in real-life practices. Int J Soc Res Methodol. 2024;28:1–17.

[5] Mulvale G, Moll S, Phoenix M, Buettgen A, Freeman B, Murray-Leung L, et al. Co-creating a new Charter for equitable and inclusive co-creation: insights from an international forum of academic and lived experience experts. BMJ Open. 2024;14: e078950. 38508634 10.1136/bmjopen-2023-078950 PMC10953044

[6] Akoglu C, Dankl K. Co-creation for empathy and mutual learning: a framework for design in health and social care. CoDesign. 2021;17:296–312.

[7] Pearce T, Maple M, Shakeshaft A, Wayland S, McKay K. What is the co-creation of new knowledge? a content analysis and proposed definition for health interventions. Int J Environ Res Public Health. 2020;17:2229. 32224998 10.3390/ijerph17072229 PMC7177645

[8] Agnello DM, Anand-Kumar V, An Q, de Boer J, Delfmann LR, Longworth GR, et al. Co-creation methods for public health research — characteristics, benefits, and challenges: a Health CASCADE scoping review. BMC Med Res Methodol. 2025;25:60. 40050729 10.1186/s12874-025-02514-4 PMC11884017

[9] Smith N, Georgiou M, Jalali MS, Chastin S. Planning, implementing and governing systems-based co-creation: the DISCOVER framework. Health Res Policy Syst. 2024;22:6. 38191430 10.1186/s12961-023-01076-5 PMC10773095

[10] Agnello DM, Balaskas G, Steiner A, Chastin S. Methods used in co-creation within the health CASCADE co-creation database and gray literature: systematic methods overview. Interact J Med Res. 2024;13: e59772. 39527793 10.2196/59772 PMC11589503

[11] Vaughn LM, Jacquez F. Participatory research methods – choice points in the research process. J Particip Res Methods. 2020;21:1.

[12] Grindell C, Coates E, Croot L, O’Cathain A. The use of co-production, co-design and co-creation to mobilise knowledge in the management of health conditions: a systematic review. BMC Health Serv Res. 2022;22:877. 35799251 10.1186/s12913-022-08079-y PMC9264579

[13] Grcheva O, Oktay VB. From public participation to co-creation in the cultural heritage management decision-making process. Sustainability. 2021;13:9321.

[14] Torfing J, Ferlie E, Jukić T, Ongaro E. A theoretical framework for studying the co-creation of innovative solutions and public value. Policy Polit. 2021;49:189–209.

[15] Lo K, Karnon J. in-DEPtH framework: evidence-informed, co-creation framework for the design, evaluation and procurement of health services. BMJ Open. 2019;9: e026482. 31061038 10.1136/bmjopen-2018-026482 PMC6501978

[16] Durugbo C, Pawar K. A unified model of the co-creation process. Expert Syst Appl. 2014;41:4373–87.

[17] Lee J-J, Jaatinen M, Salmi A, Mattelmäki T, Smeds R, Holopainen M. Design choices framework for co-creation projects. Int J Des. 2018;12:15–31.

[18] de Koning J, Crul M, Wever R. Models of co-creation ServDes. In: Morelli N, de Götzen A, Grani F, editors. Proc of the ServDes Copenhagen. Norrköping: Linköping University Electronic Press; 2016.

[19] Durugbo C. Modelling user participation in organizations as networks. Expert Syst Appl. 2012;39:9230–45.

[20] Payne AF, Storbacka K, Frow P. Managing the co-creation of value. J Acad Mark Sci. 2008;36:83–96.

[21] Wong C-C, Kumpulainen K, Kajamaa A. Collaborative creativity among education professionals in a co-design workshop: a multidimensional analysis. Think Ski Creat. 2021;42: 100971.

[22] Steiner AA, Farmer J. Engage, participate, empower: modelling power transfer in disadvantaged rural communities. Environ Plann C: Polit Space. 2018;36:118–38.

[23] Darlington E, Masson J. What does co-creation mean? An attempt at definition informed by the perspectives of school health promoters in France. Health Educ J. 2021;80:746–58.

[24] Chong-Wen C. Guidance on the conceptual design of sustainable product-service systems. Sustainability. 2018;10:2452.

[25] Greenhalgh T, Jackson C, Shaw S, Janamian T. Achieving research impact through co-creation in community-based health services: literature review and case study. Milbank Q. 2016;94(2):392–429. 27265562 10.1111/1468-0009.12197 PMC4911728

[26] Brandsen T, Steen T, Verschuere B, editors. Co-production and co-creation: engaging citizens in public services. New York: Routledge; 2020.

[27] Bilous R, Hammersley L, Lloyd K, Rawlings-Sanaei F, Downey G, Amigo M, et al. ‘All of us together in a blurred space’: principles for co-creating curriculum with international partners. Int J Acad Dev. 2018;23:165–78.

[28] Robert G, Williams O, Lindenfalk B, Mendel P, Davis LM, Turner S, et al. Applying elinor ostrom’s design principles to guide co-design in health(care) improvement: a case study with citizens returning to the community from jail in Los Angeles county. Int J Integr Care. 2021;21:7. 33613139 10.5334/ijic.5569 PMC7879991

[29] Jekal M, Brandewie B, Kim I. Exploring the future of fashion design education for generation Z through ‘Co-design as community-based participatory research. CoDesign. 2023;19:1–17.

[30] Kwon J. The effect of value co-creation and service quality on customer satisfaction and commitment in healthcare management. PhD dissertation. University of North Texas; 2015.

[31] Vargas C, Whelan J, Brimblecombe J, Allender S. Co-creation, co-design, co-production for public health – a perspective on definition and distinctions. Public Health Res Pract. 2022;32:2. 10.17061/phrp3222211 35702744

[32] Nilsen P. Making sense of implementation theories, models and frameworks. Implement Sci. 2015;10:53. 25895742 10.1186/s13012-015-0242-0 PMC4406164

[33] Participate. Dictionary. 2025. https://dictionary.cambridge.org/dictionary/english/participate. Accessed 22 May 2025.

[34] Weidenstedt L. Empowerment gone bad: communicative consequences of power transfers. Socius Sociol Res Dyn World. 2016;2:23780.

[35] Amorim J, Ventura AC. Co-created decision-making: from co-production to value co-creation in health care. J Med Access. 2023;7: 27550834231177503. 37323851 10.1177/27550834231177503 PMC10262615

[36] Nguyen TV, Benchoufi M, Young B, Chall LE, Ravaud P, Boutron I. 63 Methods of mobilising collective intelligence through crowdsourcing in research:a scoping review. BMJ Evid-Based Med. 2018;23(Suppl 1):A31–2.

[37] Harvey S, Kou C-Y. Collective engagement in creative tasks: the role of evaluation in the creative process in groups. Adm Sci Q. 2013;58:346–86.

[38] Reiter E. A structured review of the validity of BLEU. Comput Linguist. 2018;44:393–401.

[39] Moher D, Liberati A, Tetzlaff J, Altman DG. Preferred reporting items for systematic reviews and meta-analyses: the PRISMA statement. PLOS Med. 2009;6: e1000097. 19621072 10.1371/journal.pmed.1000097 PMC2707599

[40] Agnello D. Co-Creation Methods Inventory: Sourced from Academic and Grey Literature. 2023.

[41] Lyn PC. A modified Delphi approach to a new card sorting methodology. J User Exp. 2008;4:7–30.

[42] Maze | The continuous product discovery platform. Maze. https://maze.co/. Accessed 24 Oct 2023.

[43] Timotheou S, Ioannou A. Collective creativity in STEAM Making activities. J Educ Res. 2021;114:130–8.

[44] Skarzauskiene A, Kalinauskas M. Fostering collective creativity through gamification. 2014.

[45] Duarte AMB, Brendel N, Degbelo A, Kray C. Participatory design and participatory research: an HCI case study with young forced migrants. ACM Trans Comput-Hum Interact. 2018;3(1–3):39.

[46] Alex. Flow’s Engagement Thresholds Model – Flow Associates. https://flowassociates.com/2021/07/flows-engagement-thresholds-model/. Accessed 26 Oct 2023.

[47] Costa GB, Smithyman R, O’Neill SL, Moreira LA. How to engage communities on a large scale? Lessons from World Mosquito Program in Rio de Janeiro. Brazil Gates Open Research. 2021;4:109. 33103066 10.12688/gatesopenres.13153.1 PMC7569240

[48] Participation Models: Citizens, Youth, Online. 2012.

[49] Stuart G. What is the Spectrum of Public Participation? Sustaining Community. 2017. https://sustainingcommunity.wordpress.com/2017/02/14/spectrum-of-public-participation/. Accessed 25 Oct 2023.

[50] Dooris M, Heritage Z. Healthy cities: facilitating the active participation and empowerment of local people. J Urban Health. 2013;90:74–91. 22125115 10.1007/s11524-011-9623-0 PMC3764265

[51] Carter C. The Power and Pitfalls of Participatory Processes. Aberdeen: Macaulay Institute for Land Use Research; 2023.

[52] Bojovic D, Clair AL, Christel I, Terrado M, Stanzel P, Gonzalez P, et al. Engagement, involvement and empowerment: Three realms of a coproduction framework for climate services. Glob Environ Change. 2021;68: 102271.

[53] Muronda B. A conceptual public participation framework for ward committees to promote local government democracy. North-West University; 2017.

[54] Grove K. Agency, affect, and the immunological politics of disaster resilience. Environ Plan Soc Space. 2014;32:240.

[55] Hartson HR, Andre TS, Williges RC. Criteria for evaluating usability evaluation methods. Int J Human-Comput Interact. 2003;15:145–81.

[56] Messiha K, Chinapaw MJM, Ket HCFF, An Q, Anand-Kumar V, Longworth GR, et al. Systematic review of contemporary theories used for co-creation, co-design and co-production in public health. J Public Health. 2023;28:723–37. 10.1093/pubmed/fdad046 PMC10470345 37147918

[57] Vermeulen A. Six Essential Elements of Cross-Sectorial Co-Creation: Unleashing Collective Intelligence. Northern Dimension Partnership on Culture. 2023. https://ndpculture.org/news/six-essential-elements-of-cross-sectorial-co-creation-unleashing-collective-intelligence/. Accessed 24 Oct 2023.

[58] Viscusi G. Collective Intelligence, Crowd Dynamics, and Co-Creation: preliminary insights from a Case Study in Robotics Innovation Facilities (RIFs). 2020.

[59] Woolley AW, Aggarwal I, Malone TW. Collective intelligence and group performance. Curr Dir Psychol Sci. 2015;24:420–4.

[60] Miller LM. Five capacities for unleashing collective intelligence: a team inventory. Institute for Leadership Excellence, LLC. 2021. https://www.instituteforleadershipexcellence.com/five-capacities-for-unleashing-collective-intelligence-a-team-inventory/. Accessed 25 Oct 2023.

[61] Arnstein SR. A ladder of citizen participation. J Am Inst Plann. 1969;35:216–24.

