# Supplementary material for: Developing and validating the co-creation rainbow framework for intrinsic evaluation of methods: a health CASCADE structured review of models representing co-creation principles
Source: Health Res Policy Syst. 2025 Oct 10;23:127. doi: 10.1186/s12961-025-01381-1 (PMC12512873; doi:10.1186/s12961-025-01381-1)
Supplement: Supplementary file 1 — Additional file 1. Methods-specific models [file 12961_2025_1381_MOESM1_ESM.pdf]

Additional File 1. Methods-specific Models

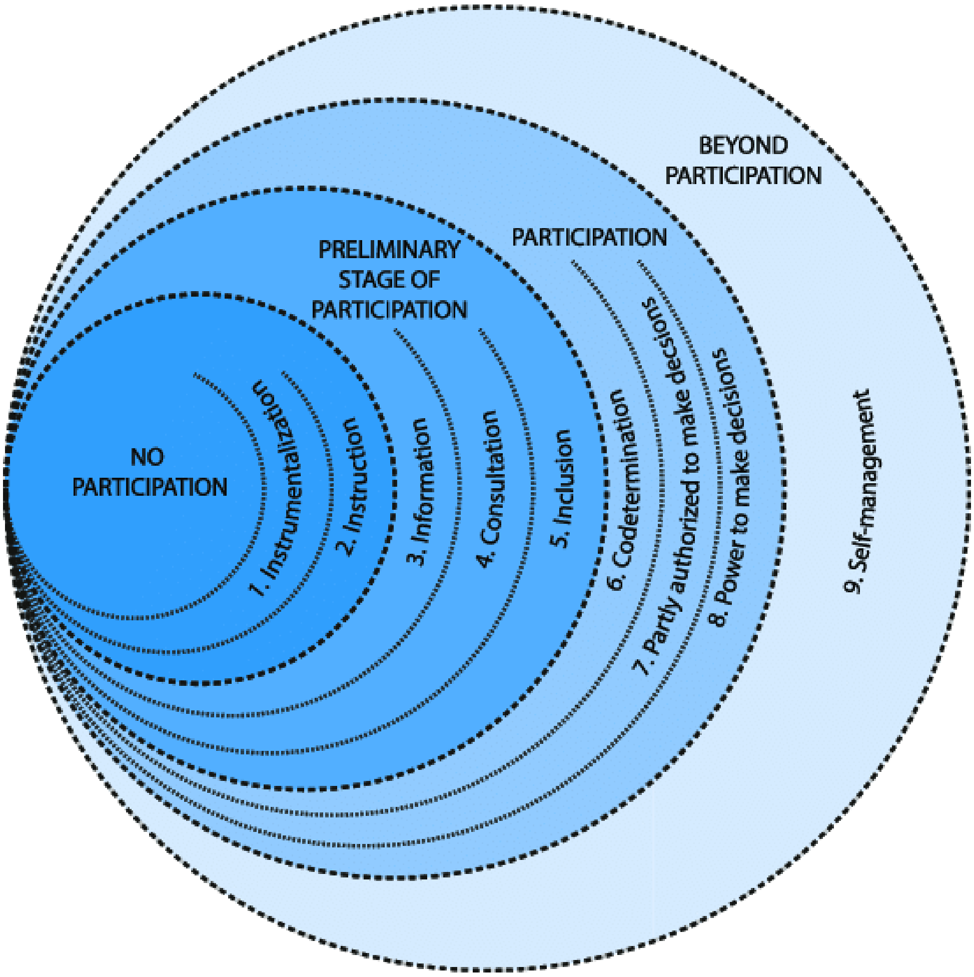

1.

Name: Stage model of participation based on Wright et al., by Duarte et al. (2018)

Source: Google Images Search > [1]

Co-Creation Characteristics: Participation and decision-making

Description:

- Instrumentalization, Instruction, and Information: levels 1 to 3 are, strictly speaking, not considered as participation. Co-researchers are consulted or asked for their opinion.
- Information, Consultation, and Inclusion: levels 3 to 5 are valuable prerequisites for participatory processes. Co-researchers are consulted or asked for their opinion.
- Codetermination: Once a process reaches level 6, it can be regarded as being truly participatory. At this level, practice partners or community partners are (partially) given the power to make decisions. co-researchers are not only consulted or asked for their opinion (as it applies to the preliminary stages). They are also encouraged to make decisions concerning the research process while liaising with the researchers.
- Partly authorized to make decisions: At level 7, they are authorized to implement minor project components of the research process at their discretion.

- Power to make decisions: Reaching level 8, the power and control over decisions shift from researchers to co-researchers. At this level, practice- or community partners make important decisions essentially by themselves, while researchers assume the role of mere consultants.
- Self-management: Level 9 surpasses participation, as individuals take full responsibility and possess total decision power. At this stage, researchers are no longer involved.

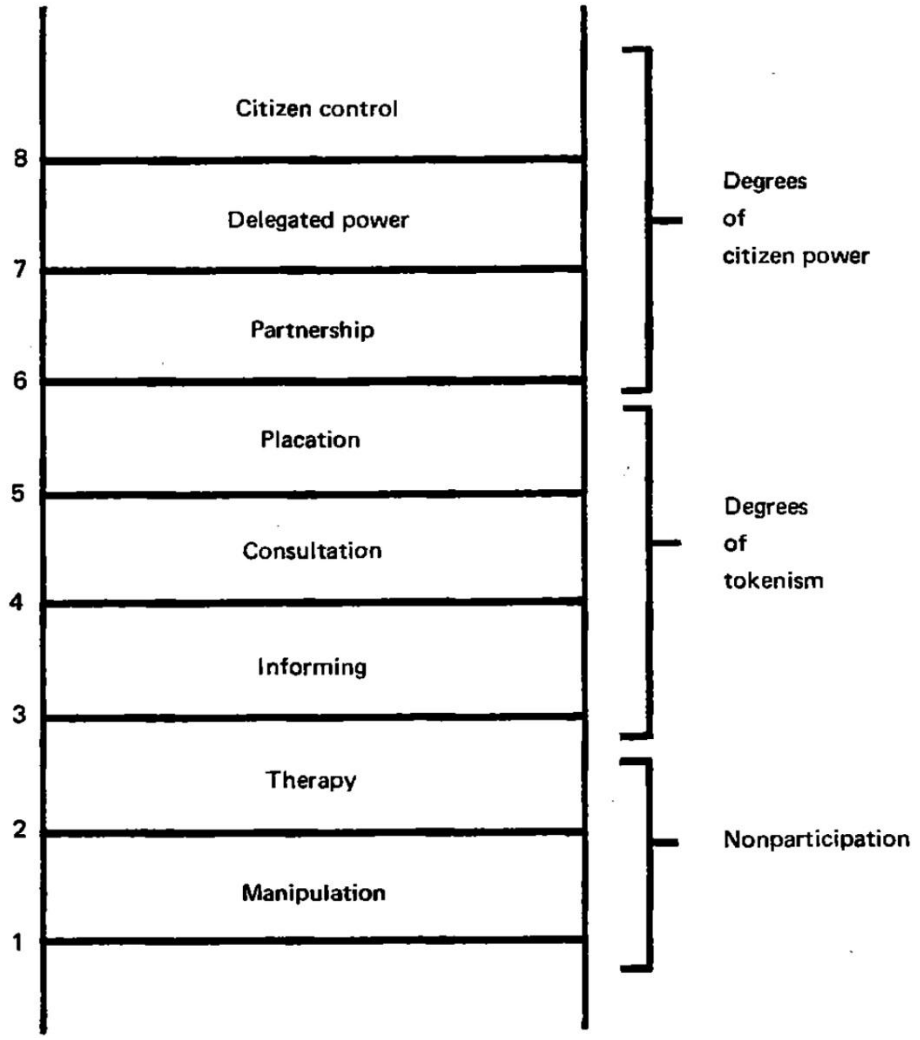

2.

Name: Ladder of Citizen Participation, by Arnstein (1969)

Source: Google Images Search > [2]

Co-Creation Characteristics: Participation and decision-making

Description:

- Manipulation: An "illusory" form of participation, manipulation occurs when public institutions, officials, or administrators mislead citizens into believing they are being given power in a process that has been intentionally manufactured to deny them power.
- Therapy: Participation as therapy occurs when public officials and administrators "assume that powerlessness is synonymous with mental illness," and they create pseudo-participatory programs that attempt to convince citizens that they are the problem when in fact it's established institutions and policies that are creating the problems for citizens.

- Informing: While Arnstein acknowledges that informing "citizens of their rights, responsibilities, and options can be the most important first step toward legitimate citizen participation," she also notes that "too frequently the emphasis is placed on a one-way flow of information—from officials to citizens—with no channel provided for feedback and no power for negotiation…meetings can also be turned into vehicles for one-way communication by the simple device of providing superficial information, discouraging questions, or giving irrelevant answers."
- Consultation: When consultation processes "are not combined with other modes of participation, this rung of the ladder is still a sham since it offers no assurance that citizen concerns and ideas will be taken into account. The most frequent methods used for consulting people are attitude surveys, neighbourhood meetings, and public hearings. When power holders restrict the input of citizens' ideas solely to this level, participation remains just a window-dressing ritual. People are primarily perceived as statistical abstractions, and participation is measured by how many come to meetings, take brochures home, or answer a questionnaire.
- Placation: Placation occurs when citizens are granted a limited degree of influence in a process, but their participation is largely or entirely tokenistic: citizens are merely involved only to demonstrate that they were involved. In Arnstein's words: "An example of placation strategy is to place a few hand-picked 'worthy' poor on boards of Community Action Agencies or public bodies like the board of education, police commission, or housing authority. If they are not accountable to a constituency in the community and if the traditional power elite holds the majority of seats, the have-nots can be easily outvoted and outfoxed."
- Partnership: Participation as a partnership occurs when public institutions, officials, or administrators allow citizens to negotiate better deals, veto decisions, share funding, or put forward requests that are at least partially fulfilled. In Arnstein's words: "At this rung of the ladder, power is redistributed through negotiation between citizens and powerholders. They agree to share planning and decision-making responsibilities through such structures as joint policy boards, planning committees, and mechanisms for resolving impasses." Arnstein does note, however, that in many partnership situations, power is not voluntarily shared by public institutions, but rather taken by the citizens through actions such as protests, campaigns, or community organizing.
- Delegated Power: Participation as delegated power occurs when public institutions, officials, or administrators give up at least some degree of control, management, decision-making authority, or funding to citizens. For example, a citizen board or corporation that is tasked with managing a community program, rather than merely participating in a program managed by a city, would be an example of delegated power.
- Citizen Control: Arnstein's words, when "participants or residents can govern a program or an institution, be in full charge of policy and managerial aspects, and be able to negotiate the conditions under which 'outsiders' may change them." In citizen-control situations, for example, public funding would flow directly to a community organization, and that organization would have full control over how that funding is allocated.

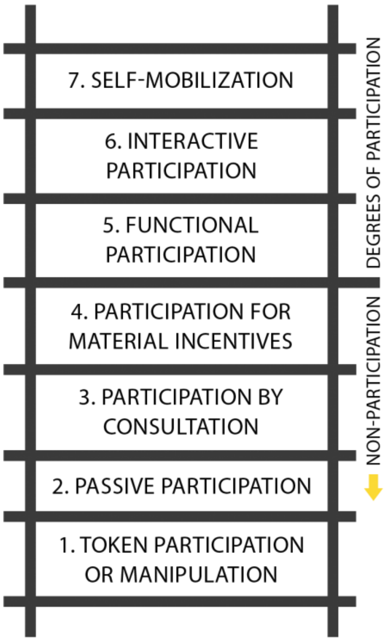

3.

Name: Typology of Participation, by Pretty (1995)

Source: Google Images Search > [3,4]

Co-Creation Characteristics: Participation

Description:

- Manipulative participation: a pretence, unelected and powerless individuals.
- Passive participation: people just receive decisions in which they have had no part.
- Participation by consultation: no sharing of problem definition nor analysis of responses.
- Participation for material incentives: cash or other bonuses but only in the short term.
- Functional participation: not real power-sharing; looks good but decisions are taken externally.
- Interactive participation: joint responsibility for defining and achieving goals, analysis, and stakeholder development.
- Self-mobilization: independent initiatives, external agents invited in, local control of resources and outcomes.

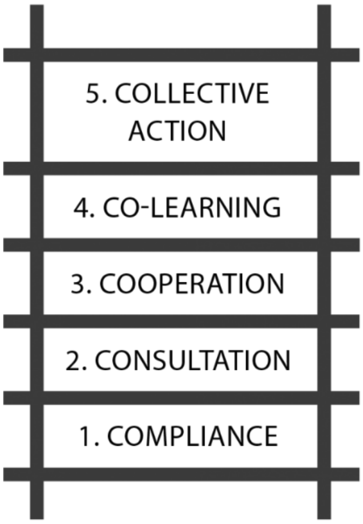

4.

Name: A ladder, by Kanji and Greenwood (2001)

Source: Google Images Search > [3]

Co-Creation Characteristics: Participation

Description:

- Compliance: where tasks with incentives are assigned but the agenda and process are directed by outsiders.
- Consultation: where local opinions are sought, outsiders analyse and decide the course of action.
- Cooperation: where local people work with outsiders to determine priorities; the responsibility to direct the process lies with outsiders.
- Co-learning: where local people and outsiders share knowledge, create new understanding and work together to form action plans.
- Collective action: where local people set their own agenda and mobilise to carry it out in the absence of outsiders.

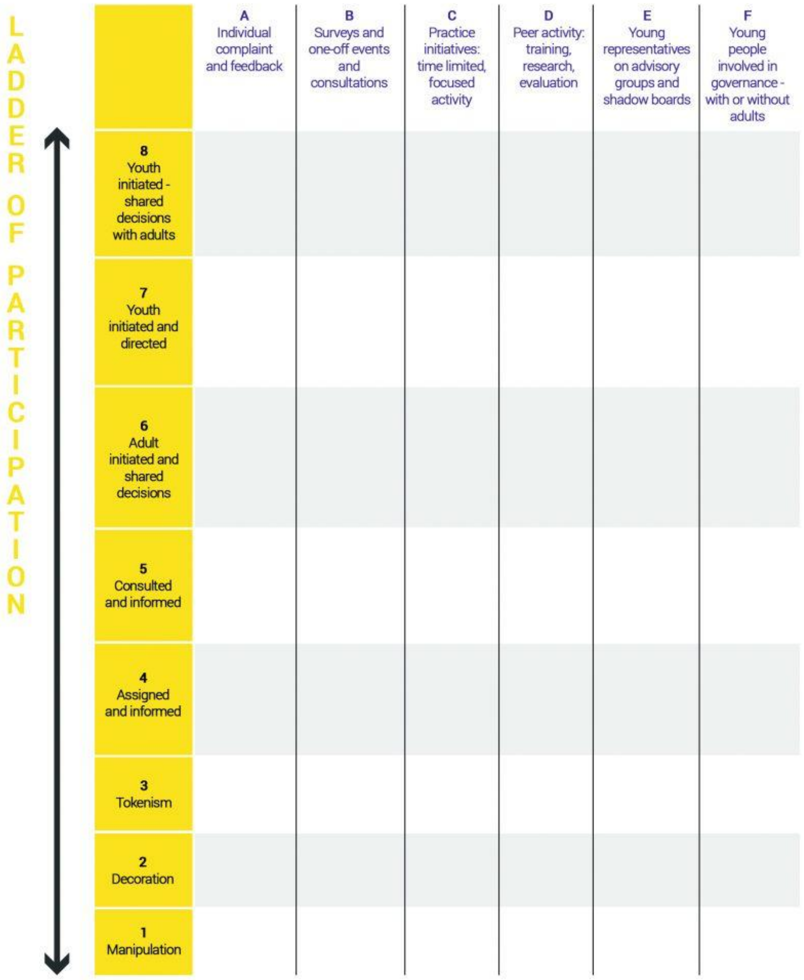

5.

Name: Matrix of Participation, by Badham and Davies (2007)

Source: Google Images Search > [3]

Co-Creation Characteristics: Participation an empowerment

Description: When using the Matrix, participants could use different colours to differentiate between one-off, short- and long-term initiatives and approaches. This continuum commences at the left-hand side, representing one-off and short-term events or activities; and progresses to capture more structured, intensive, and long-term initiatives on the right. A spread of engagement across the Matrix is likely to evidence an organizational practice that is dynamic, energetic, and responsive to the needs of young people; such a mix illustrates a commitment to a sustainable practice that shares responsibility with young people.

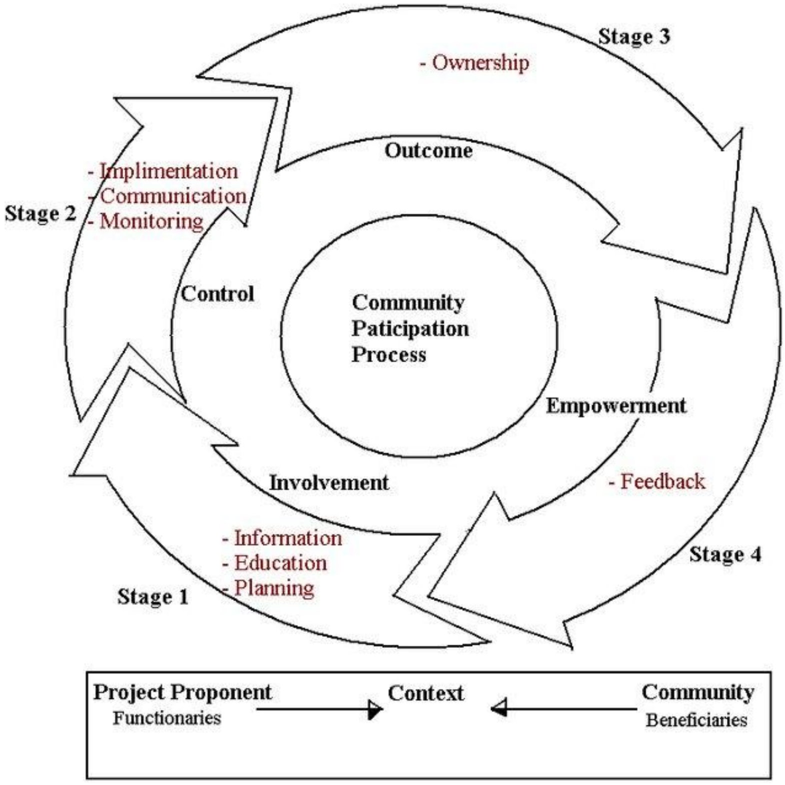

6.

Name: An Effective Community Participation Model (World Bank, 2004)

Source: Google Images Search > [5]

Co-Creation Characteristics: Participation and Empowerment

Description:

- **Stage 1: Information, Education and Planning (IEP) Stage** = provides adequate and timely information, educating people about the development initiatives and outlining a plan of action which is critical in generating a process of participation.
- **Stage 2: Implementation, Coordination and Monitoring (ICM) Stage** = which holds that once local people are well informed about the development project, they are in a better position to plan activities by themselves to implement in a project.
- **Stage 3: Ownership and Control Stage =** where local people should share the project cost if not in monetary terms, at least in time and effort. This sharing of cost will give them a feeling of ownership, and belonging and commit them to the project.
- **Stage 4: Feedback Stage of Participation** = includes consultations with local people to assess their needs and evaluate the outcome of development projects, and hold local people accountable for successes and failures.

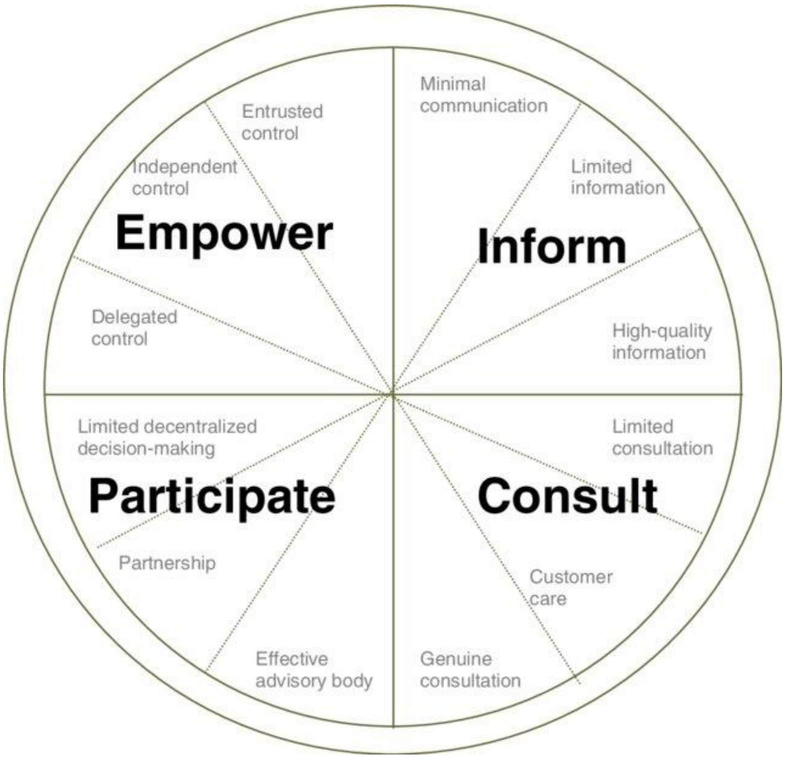

7.

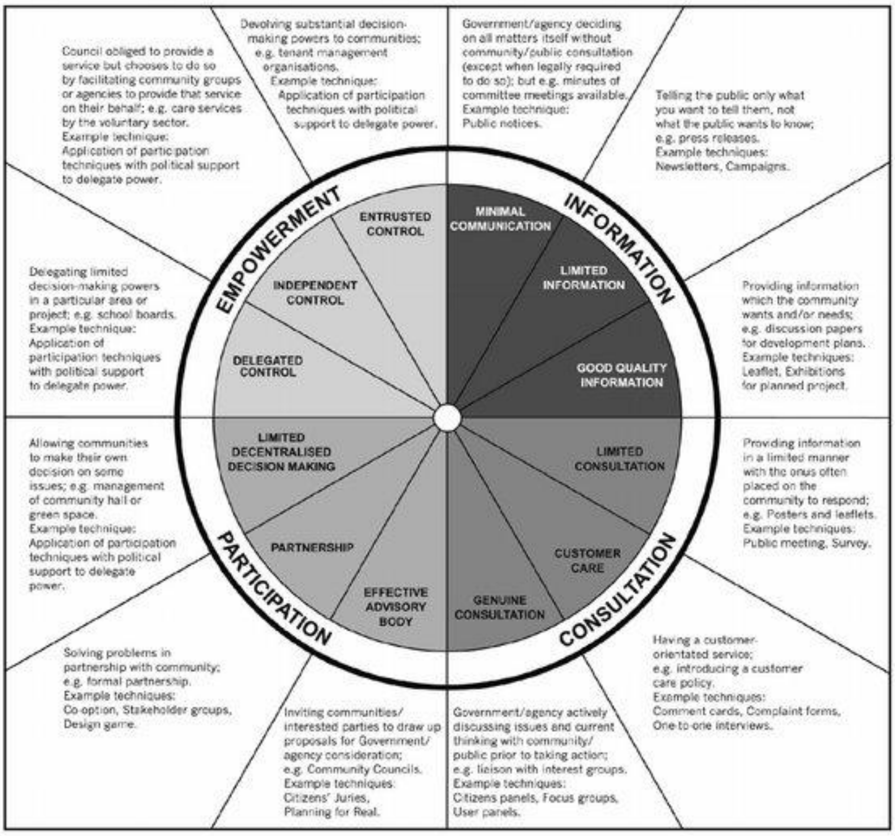

Name: Wheel of Participation, by Davidson (1998)

Source: Google Images Search > [6–9]

Co-Creation Characteristics: Participation and Empowerment

Description:

- Minimal Communication: Government/agency deciding on all matters itself without community/public consultation (except when legally required to do so); but e.g. minutes of committee meetings available. Example techniques: Public notices.
- Limited Information: Telling the public only what you want to tell them, not what the public wants to know; e.g. press releases. Example techniques = Newsletters and campaigns.
- Good Quality Information: Providing information that the community wants and/or needs; e.g. discussion papers for development plans. Example techniques: Leaflets and exhibitions for planned projects.
- Limited Consultation: Providing information in a limited manner with the onus often placed on the community to respond; e.g. Posters and leaflets. Example techniques: Public meeting, Survey.
- Customer Care: Having a customer-oriented service; e.g. introducing a customer care policy. Example techniques: Comment cards, Complaint forms, and One-to-One interviews.
- Genuine Consultation: Government/agency actively discussing issues and current thinking with the community/public before taking action; e.g. liaison with interest groups. Example techniques: Citizen panels, Focus groups, and User panels.
- Effective Advisory Body: Inviting communities/interested parties to draw up proposals for government/agency consideration; e.g. community councils. Example techniques: Citizens' Juries, Planning for Real.
- Partnership: Solving problems in partnership with the community; e.g. formal partnership. Example techniques: co-option, stakeholder groups, and design game.
- Limited Decentralised Decision Making: Allowing communities to make their own decision on some issues; e.g. management of community hall or green space. Example technique: Application of participation techniques with political support to delegate power.
- Delegated Control: Delegating limited decision-making powers in a particular area or project; e.g. school boards. Example technique: Application of participation techniques with political support to delegate power.
- Independent Control: The Council is obliged to provide a service but chooses to do so by facilitating community groups or agencies to provide that service on their behalf; e.g. care services by the voluntary sector. Example technique: Application of participation techniques with political support to delegate power.
- Entrusted Control: Devolving substantial decision-making powers to communities; e.g. tenant management organization. Example technique = Application of participation techniques with political support to delegate power.

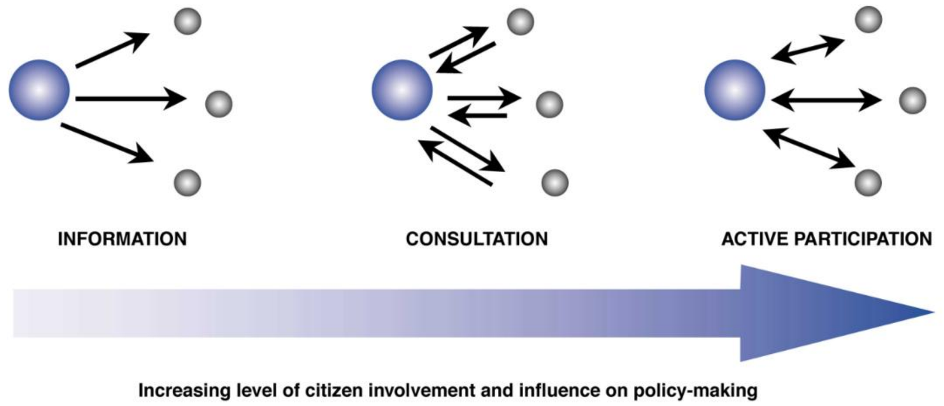

8.

Name: Analytical framework for conducting comparative surveys and country case studies

Source: Google Images Search > [7,10]

Co-Creation Characteristics: Participation

Description:

- Information: Information is a one-way relationship in which the government produces and delivers information for use by citizens. It covers both “passive” access to information upon demand by citizens and “active” measures by the government to disseminate information to citizens. Examples include: access to public records, official gazettes, and government websites.
- Consultation: Consultation is a two-way relationship in which citizens provide feedback to the government. It is based on the prior definition by the government of the issue on which citizens’ views are being sought and requires the provision of information. Governments define the issues for consultation, set the questions and manage the process, while citizens are invited to contribute their views and opinions. Examples include: public opinion surveys and comments on draft legislation.
- Active Participation: Active participation is a relation based on partnership with government, in which citizens actively engage in defining the process and content of policy-making. It acknowledges equal standing for citizens in setting the agenda, proposing policy options, and shaping the policy dialogue – although the responsibility for the final decision or policy formulation rests with the government. Examples include: consensus conferences, and citizens’ juries.”

9. 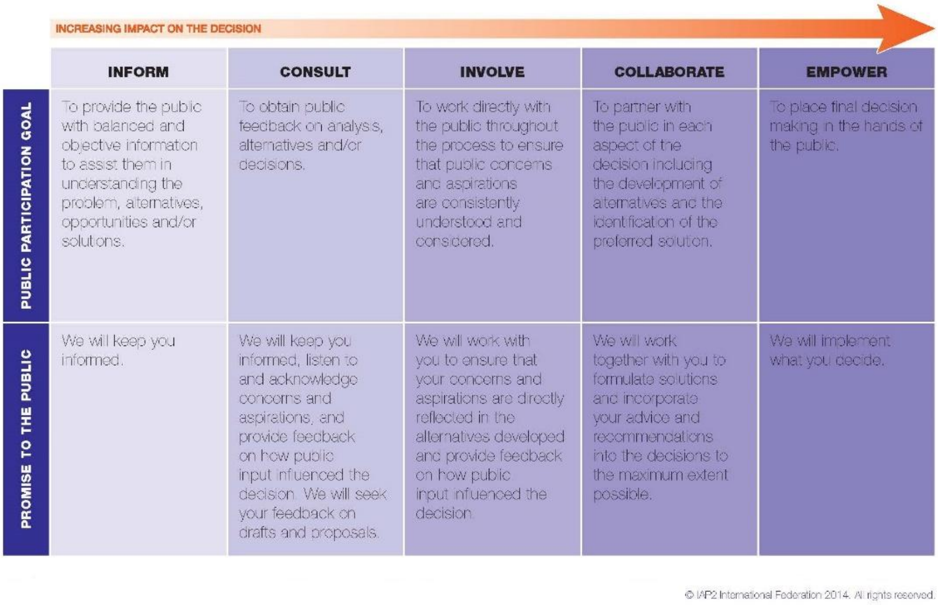

INCREASING IMPACT ON THE DECISION

	INFORM	CONSULT	INVOLVE	COLLABORATE	EMPOWER
PUBLIC PARTICIPATION GOAL	To provide the public with balanced and objective information to assist them in understanding the problem, alternatives, opportunities and/or solutions.	To obtain public feedback on analysis, alternatives and/or decisions.	To work directly with the public throughout the process to ensure that public concerns and aspirations are consistently understood and considered.	To partner with the public in each aspect of the decision including the development of alternatives and the identification of the preferred solution.	To place final decision making in the hands of the public.
PROMISE TO THE PUBLIC	We will keep you informed.	We will keep you informed, listen to and acknowledge concerns and aspirations, and provide feedback on how public input influenced the decision. We will seek your feedback on drafts and proposals.	We will work with you to ensure that your concerns and aspirations are directly reflected in the alternatives developed and provide feedback on how public input influenced the decision.	We will work together with you to formulate solutions and incorporate your advice and recommendations into the decisions to the maximum extent possible.	We will implement what you decide.

© IAP2 International Federation 2014. All rights reserved.

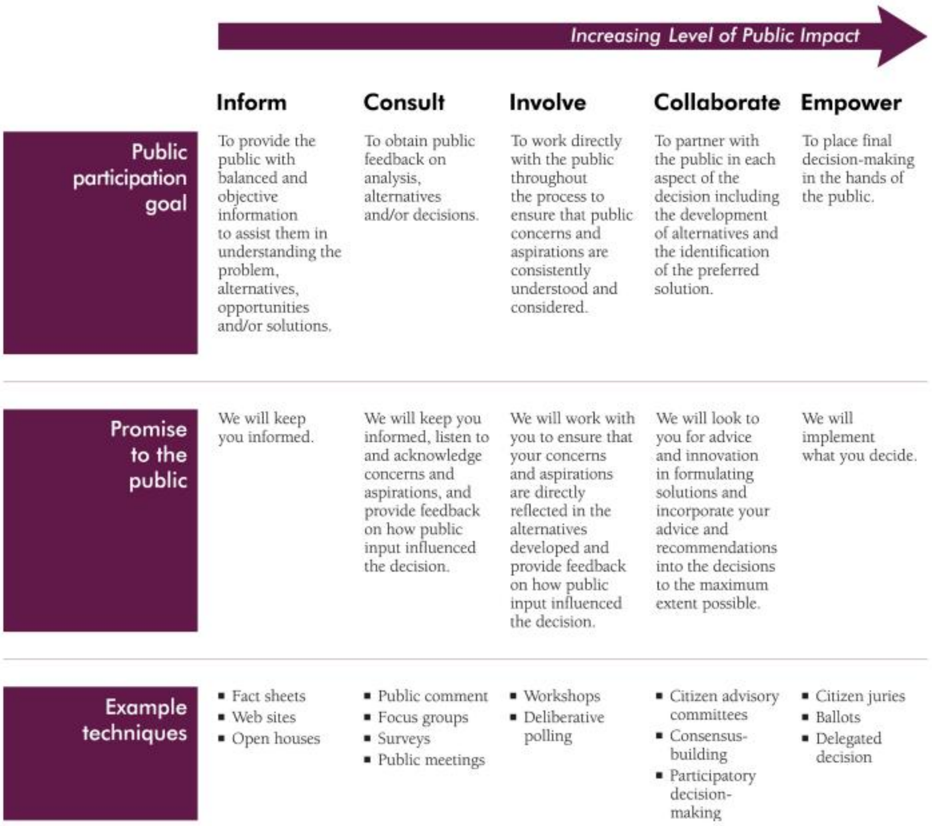

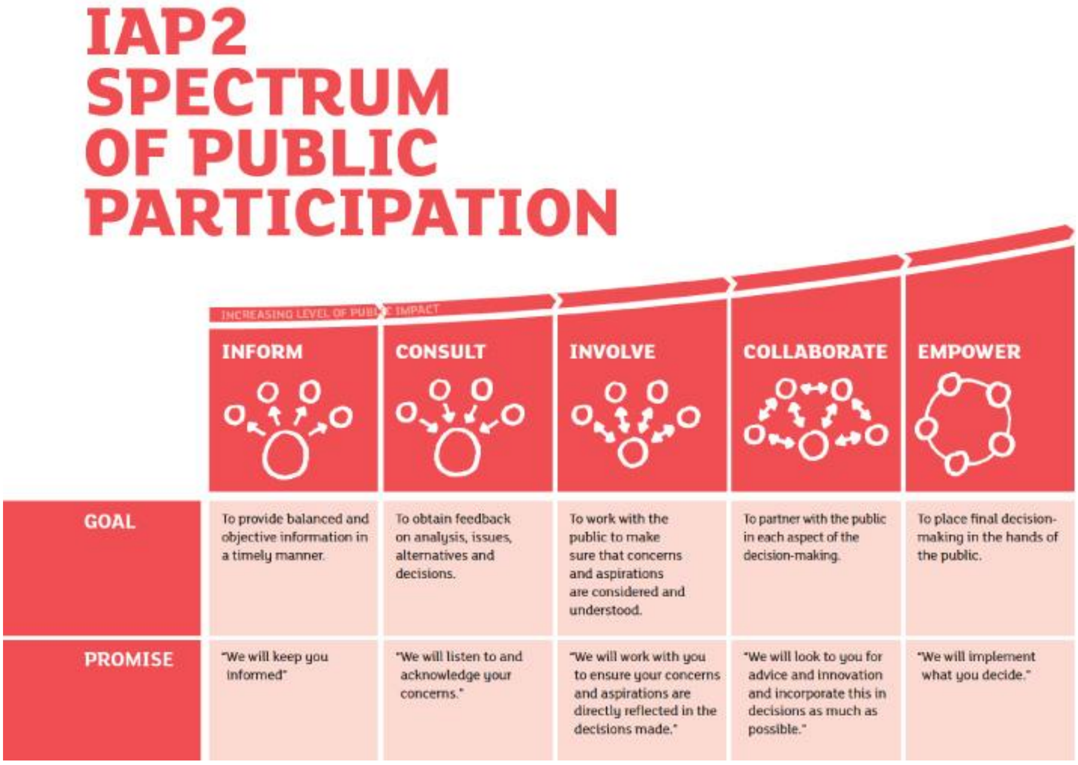

Name: IAP2 – Spectrum of Public Participation, by the International Association for Public Participation (2007)

Source: Google Images Search > [7,11]

Co-Creation Characteristics: Participation, decision-making, and empowerment

Description:

- Inform: To provide the public with balanced and objective information to assist them in understanding the problem, alternatives, opportunities, and/or solutions. Examples = Fact sheets, websites, and open houses.
- Consult: To obtain public feedback on analysis, alternatives, and/or decisions. Examples = public comment, focus groups, surveys, and public meetings.
- Involve: To work directly with the public throughout the process to ensure that public concerns and aspirations are consistently understood and considered. Examples = Workshops and Deliberative polling.
- Collaborate: To partner with the public in each aspect of the decision including the development of alternatives and the identification of the preferred solution. Examples = Citizen advisory committees, Consensus-building, and participatory decision-making.
- Empower: To place final decision-making in the hands of the public. Examples = Citizen juries, ballots, and delegated decisions.

10.

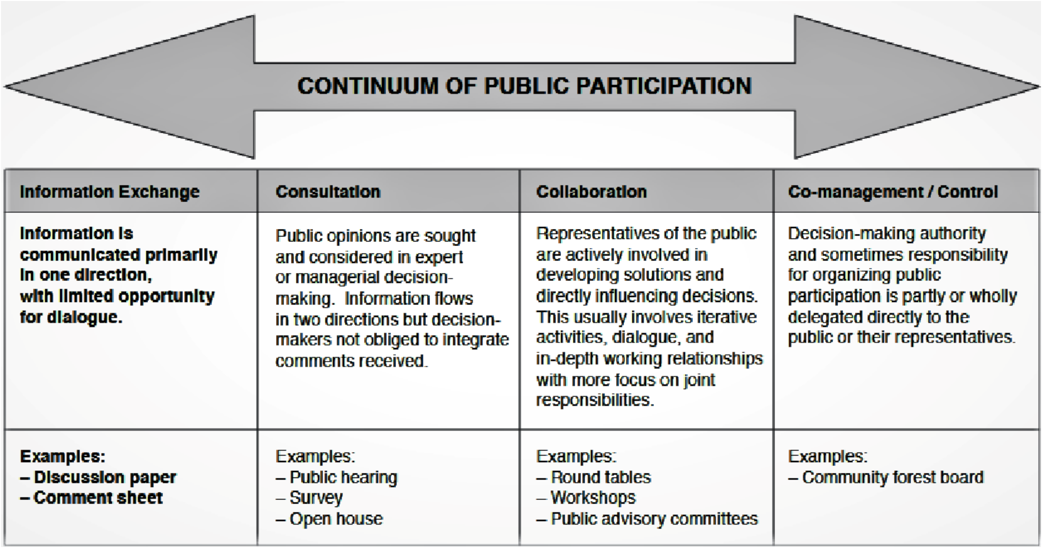

Information Exchange	Consultation	Collaboration	Co-management / Control
**Information is communicated primarily in one direction, with limited opportunity for dialogue.**	Public opinions are sought and considered in expert or managerial decision-making. Information flows in two directions but decision-makers not obliged to integrate comments received.	Representatives of the public are actively involved in developing solutions and directly influencing decisions. This usually involves iterative activities, dialogue, and in-depth working relationships with more focus on joint responsibilities.	Decision-making authority and sometimes responsibility for organizing public participation is partly or wholly delegated directly to the public or their representatives.
**Examples:**<br>**– Discussion paper**<br>**– Comment sheet**	Examples:<br>– Public hearing<br>– Survey<br>– Open house	Examples:<br>– Round tables<br>– Workshops<br>– Public advisory committees	Examples:<br>– Community forest board

Name: Continuum of Public Participation, by Muronda (2017)

Source: Google Images Search > [12]

Co-Creation Characteristics: Participation and decision-making

Description:

- Information Exchange: Information is communicated primarily in one direction, with limited opportunity for dialogue. Examples = discussion papers and comment sheets.
- Consultation: Public opinions are sought and considered in expert or managerial decision-making. Information flows in two directions but decision-makers are not obliged to integrate comments received. Examples = Public hearing, survey, and an open house.
- Collaboration: Representatives of the public are actively involved in developing solutions and directly influencing decisions. This usually involved iterative activities, dialogue, and in-depth working relationships with more focus on joint responsibilities. Examples = round tables, workshops, and public advisory committees.

- Co-management / Control: Decision-making authority and sometimes responsibility for organizing public participation is partly or wholly delegated directly to the public or their representatives. Examples = community forest board.

11. 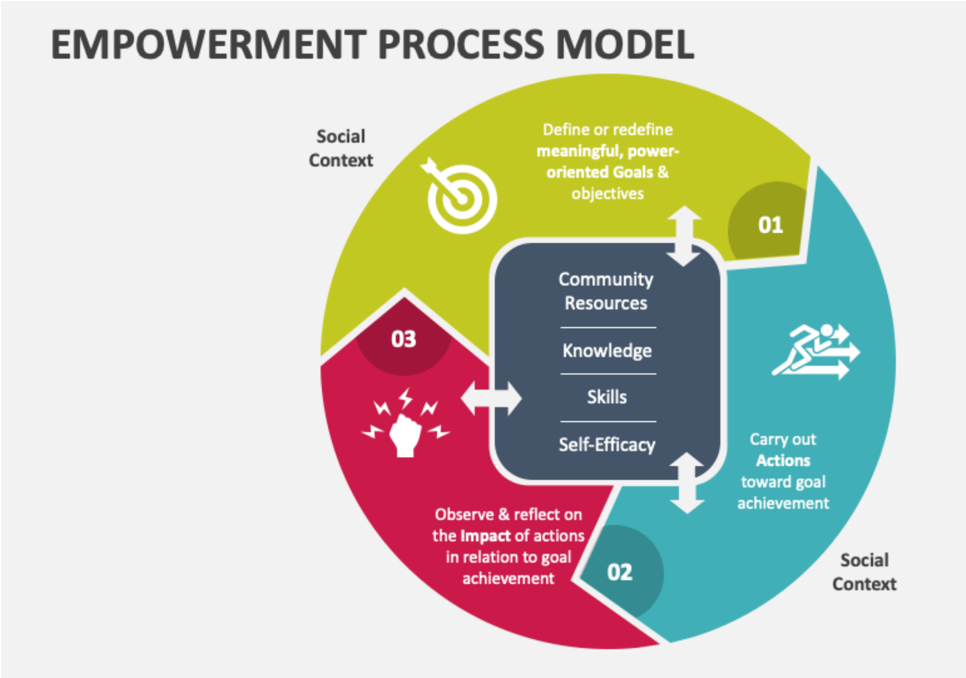

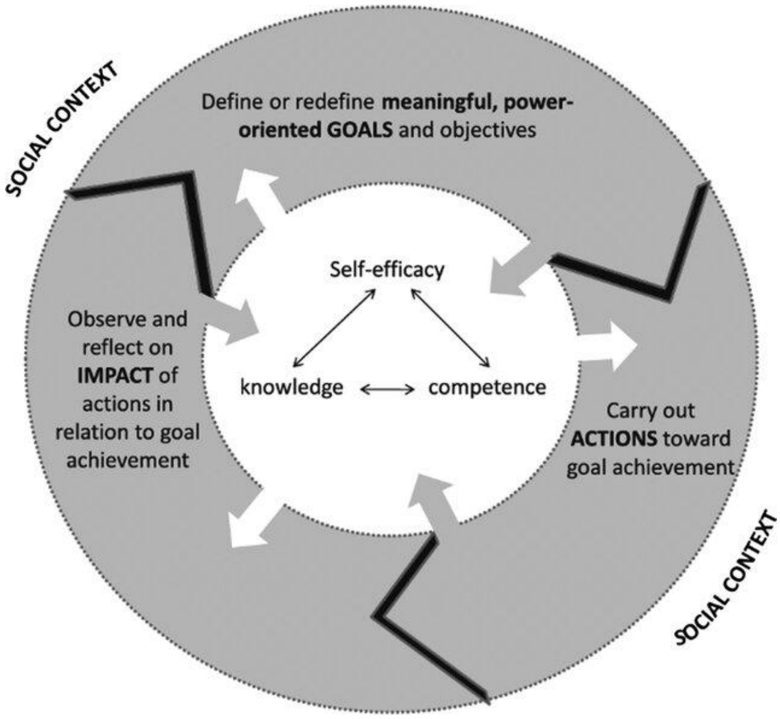

Name: The Empowerment Process Model, by Cattaneo and Goodman (2015)

Source: Google Images Search > [13]

Co-Creation Characteristics: Empowerment

Description: It is a model that clarifies the characteristics of empowerment. The source article defines empowerment as a meaningful shift in the experience of power attained through interaction in the social world. The model describes the process of building empowerment as an iterative one, in which a person who lacks power sets a personally meaningful goal-oriented toward increasing power, takes action, and makes progress toward that goal, drawing on his or her evolving self-efficacy, knowledge, skills, and community resources and supports, and observes and reflects on the impact of his or her actions. It is a process focused on specific goals, and it extends beyond the intrapsychic realm. After describing the model and the definition of power on which it rests, the article details how the model addresses challenges to the conceptualization of empowerment.

- Define or redefine meaningful, power-oriented goals and objectives.
- Carry out actions towards goal achievement.
- Observe and reflect on the impact of the actions concerning the goal achievement.

12. 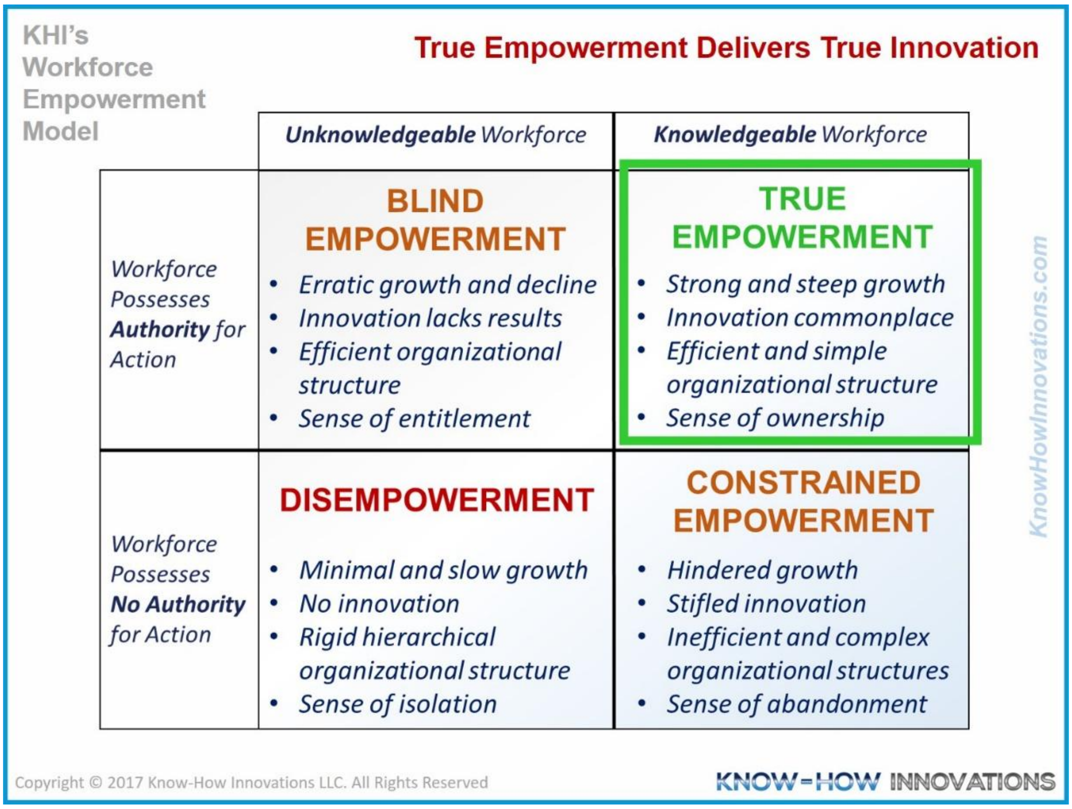

Name: Know How Innovations' Empowerment Model, 2017

Source: Google Images Search > [14]

Co-Creation Characteristics: Empowerment

Description: The matrix has two parameters, Knowledge, and Authority. Knowledge = The necessary, relevant information, skill, and experience to evaluate appropriate scenarios and make sound decisions. The gaining of knowledge is a continuous and unceasing process and, hence, must be regulated to prevent overwhelming the individual. Learning never stops. The more knowledge and experience an individual possesses the better the quality and outcomes of their decisions. Authority = The ability and willingness to independently make sound decisions and take action without prior approval of a supervisor. Authority is metered out in incrementally greater degrees as the individual gains the necessary knowledge and experience. It is a structured and controlled process.

- Disempowerment (less knowledge – less authority): When knowledge and skill progression is low priority and the workforce is allowed little to no authority, they become disempowered. Individual workers have no clear development path to learn the skills to gain greater authority within the organization. Individuals work alone with no opportunity or need for teams. This workforce feels a sense of isolation and has no loyalty to the organization.
- Constrained Empowerment (More Knowledge – Less Authority): Knowledge without authority is constrained empowerment. This workforce has no clear development path to gaining greater authority and rank within the organization. Deeply knowledgeable and highly skilled, this workforce possesses excellent problem-solving abilities and a penchant for innovation. However, they soon lose interest due to a lack of leadership encouragement, support, and forward momentum. This workforce feels stifled when their tremendous innovative potential remains untapped, which leads to a sense of abandonment.
- Blind Empowerment (Less Knowledge – More Authority): Authority without relevant knowledge is blind empowerment. Individuals feel a sense of false empowerment as they wield unbridled authority for decisions and actions. This workforce feels a strong sense of entitlement. However, this workforce lacks sufficient relevant knowledge to back their authority with sound thought and structure. In a strongly individualistic environment, employees believe that success and progression in the organization are entirely in their own hands. Team cohesion is weak in this arena with individuals jostling for personal credit and success.
- True Empowerment (More Knowledge – More Authority): Progressively greater knowledge-backed authority fuels true empowerment. This workforce possesses the knowledge to make sound decisions, actuated by their authority for relevant action. The inclusive yet independent culture creates a sense of ownership among the employees. This is an unstoppable workforce able to deliver innovation on demand and leverage the collective intellect to solve complex problems.

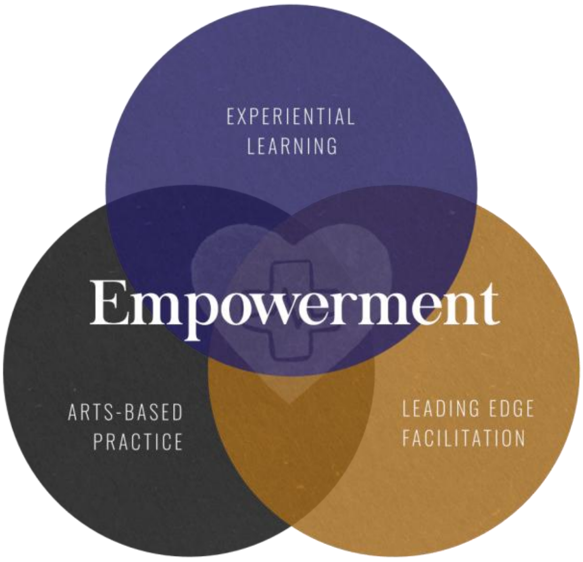

13.

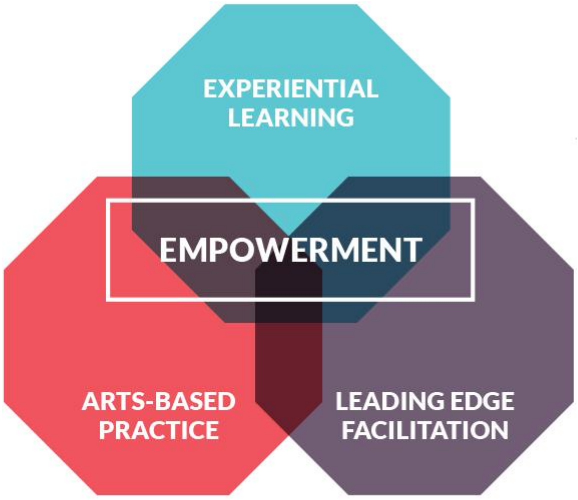

Name: Our Experience Model and Our Creative Empowerment Model

Source: Google Images Search > [15]

Co-Creation Characteristics: Empowerment

Description:

- Experiential Learning: Hands-on experiences spark new perspectives. Participation and reflection ignite possibility, offer insight, and transform attitudes, behaviours, and beliefs.
- Arts-based practice: People thrive in arts-based environments. Easy-to-lead activities drawn from art forms like poetry, visual arts, music, and theatre boost bonding and creativity, build motivation and bring joy into the process of learning.
- Leading Edge Facilitation: Social and empathetic growth happens best in well-facilitated groups. The most current techniques in leadership and group dynamics prepare practitioners to build great groups.

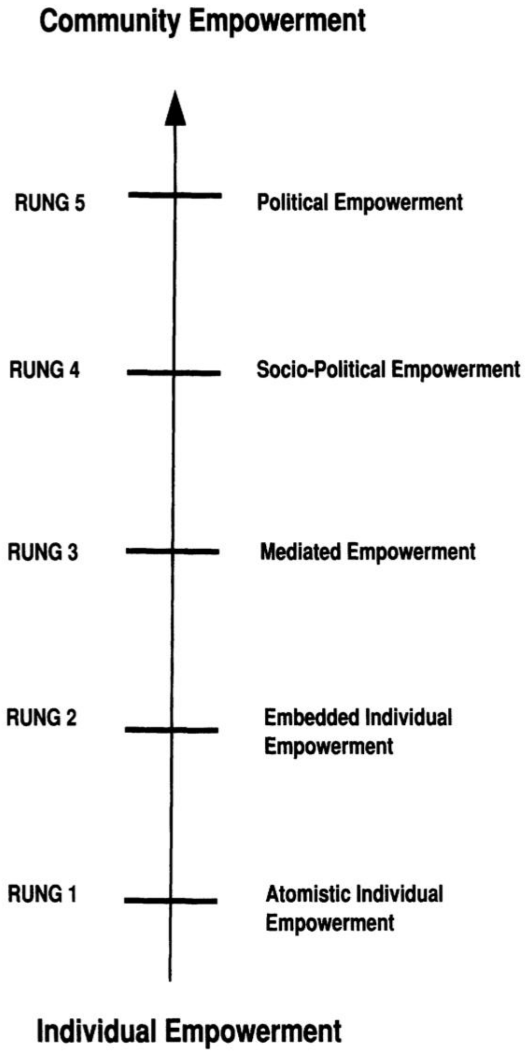

14.

Name: Ladder of Empowerment, by Rocha et al. (1997)

Source: Google Images Search > [16,17]

Co-Creation Characteristics: Empowerment

Description:

- Atomistic individual empowerment: the locus is the individual; the goal is increasing individual efficacy; and the process consists of altering the emotional or physical state of the individual. Atomistic individual empowerment is built upon the rational actor model and explains the competencies of one person. It is conceptually modelled after mental health treatment processes and refers to changing individual characteristics in the tradition of the psychology of individual differences, often taking the form of increased coping skills. Strength is gained from the support of powerful others, and feelings of power are gained through self-control.
- Embedded individual empowerment: Emphasis is placed on the individual's immediate context. Although the locus of empowerment is individual, the process clearly includes recognition of the importance of the surrounding environment. It considers the person-environment fit, contextual variables and their relationship to empowerment. Empowerment is the ability to understand one's external context, to manoeuvre through it at a heightened level of facility with the goal of increasing personal efficacy and satisfaction. Power is

typified by support and strength gained from setting and organization participation; and in which the experience of power derives from the pursuit of autonomy through self-understanding and self-directed action.

- Mediated Empowerment: Is a highly professionalized model in which the process of empowerment is mediated by an expert or professional. The locus in this type can either be the individual or community, depending upon the specific circumstances. Its goals are to provide knowledge and information necessary for individual or community decision-making and action. This revolves around the relationship, between the expert and the client/consumer, through which empowerment is realized.
- Socio-Political Empowerment: This model of individual and community empowerment focuses on what has been termed transformative populism, in which community development is conceptualized as developing the people who comprise the community as the first priority, then attending to the physical development of the neighbourhoods in which people live. This type of empowerment focuses on the process of change within a community locus in the context of a collaborative struggle to alter social, political, or economic relations. This type of empowerment uses collaborative, grassroots, political action as a benchmark, and it consists of two core elements: 1) critical reflection by the community and members-of-community (individuals) rethinking their relationship to structures of power; and 2) collective action upon those structures. The power experiences that characterize this type of empowerment are developmental in nature; at each developmental phase, a different type of power experience will predominate for the community as well as for members of the community.
- Political Empowerment: This type of empowerment is the community, conceptualized as a network of like-minded individuals with or without a geographic dimension as its defining characteristic. The process of empowerment is political action directed toward institutional change. It can be comprised of voting and voter registration. It involves expanding access to group resources, e.g. in education, housing, employment, government benefits, health care, or political representation. The focus is not on the process of change within the individual or group, but on the outcome, thus equating empowerment with visible results. The primary power experience is a group version of stage three, the community experience of competition for a winning resource, such as the control of the resource allocation process through which community resources are garnered.

15. 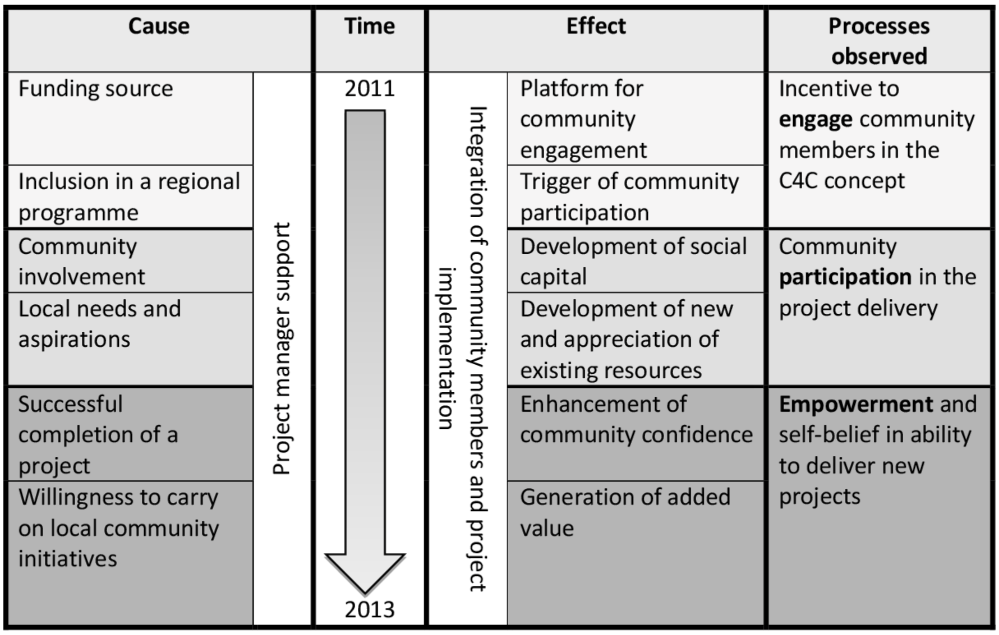

Name: Engage, Participate and Empower Model, by Author[s]
Source: Google Images Search > [18]
Co-Creation Characteristics: Empowerment and Participation
Description: Empowerment is an outcome of the process. Through the engagement and participation stimulated, there was evidence that the C4C programme grew links between different people and groups within the communities, and thus increased social capital.

- Engage: incentive to engage community members in the Capacity for Change Community development initiative. Triggered by 1) Funding as a stimulus of initial engagement; and 2) Support of the project manager and the development of interests in running a local project. Effect = platform for community engagement, and trigger of community participation. The key actor is the project manager. The observed empowerment practice is exogenous. Community development is focused on capacity building.
- Participate: Community participation in the project delivery. Triggered by 1) being part of a regional programme as a trigger of community participation; 2) confidence as an essential component to building community empowerment; and 3) development of social capital and integrated leadership through community involvement. Effect = development of social capital and appreciation of existing resources. The key actor is the project manager with increasing power of community members. The observed empowerment practice is exogenous with endogenous.
- Empower: Self-belief in the ability to deliver new projects. Triggered by 1) development of appreciation of existing resources; and 2) citizen power. Effect = Enhanced community confidence, and generation of added value. The key actors are the community members. The observed empowerment practice is endogenous.

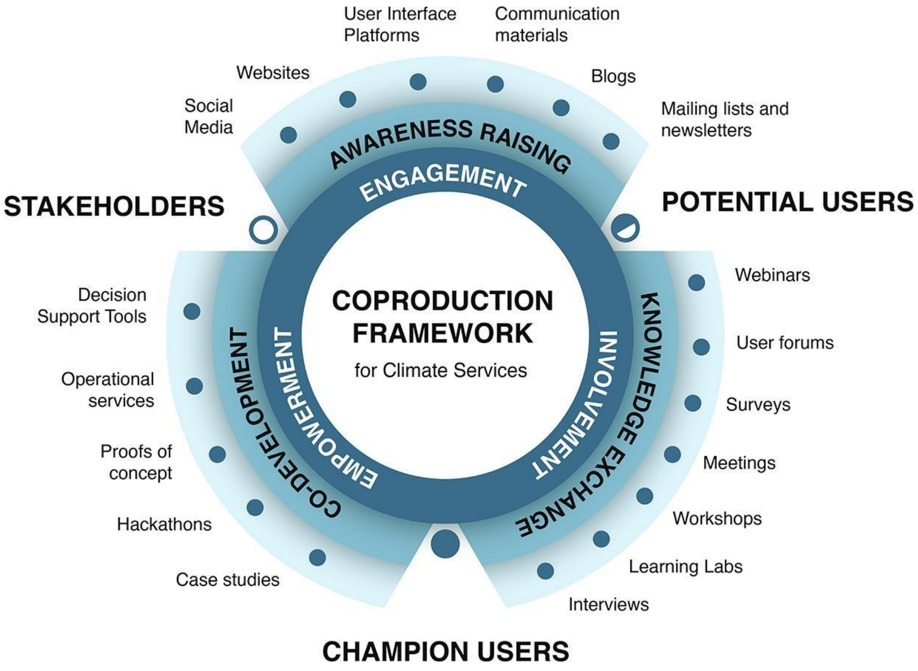

16.

Name: Coproduction Framework, by Bojovic et al. (2021)
Source: Google Images Search > [19]
Co-Creation Characteristics: Empowerment and decision-making,
Description: This is an iterative framework. The status of participants gradually changes as they move through the framework, from stakeholders to potential users, to champion users. The more we learn from the knowledge exchange and co-development of new knowledge in the involvement and empowerment realms, the more customized material we have for sharing and engaging with new stakeholders. The three realms that form the coproduction framework for climate services are not discrete phases but rather a continuum: they build on and interact with each other as the coproduction process builds throughout the cycle.

- Engagement realm: The framework engages stakeholders by raising awareness through different communication tools. Building awareness about existing initiatives and available knowledge is the first step in facilitating access to climate data. Far-reaching web tools have a key role in establishing this initial engagement of stakeholders. Information and communication technologies, epitomised by the Internet, provide an excellent opportunity for engaging citizens, raising awareness about climate information, and scaling up participation. Other approaches such as interactive user interfaces and blogs can be more effective by allowing for bi-directional and more dynamic engagement. The engagement realm should continue throughout the co-production process.
- Involvement realm: It then involves stakeholders in knowledge exchange and co-learning, using various participatory approaches. A more involved and intensive approach includes interaction with stakeholders through surveys, interviews, and meetings. An online survey can easily reach a large number of stakeholders, provided they are motivated to participate. Prior engagement activities can add to this motivation. To deepen our understanding of stakeholders' needs, perceptions, and rationales, we need a more intensive and meaningful exchange that can be achieved through interviews. Although semi-structured interviews are time-intensive, they can encourage participants to speak freely and enable the emergence of new discourses and narratives. In workshops, round-tables and focus groups, participants can actively exchange knowledge, confront opinions, sort out disagreements, build consensus and find common solutions for potentially conflicting interests and views. If skillfully facilitated, open online discussion spaces such as thematic online forums can also provide strong

involvement, while the issue of the pre-selection of participants can be addressed by such spaces being made freely open for everyone to participate.

- <u>Empowerment realm</u>: it empowers users of climate services, who take part in their co-development. From the network of participants established in the previous steps, some stakeholders want to be more actively involved in problem analysis, exploring, and identifying possible as well as preferred solutions, the so-called “champion users”. Building on the information-sharing and knowledge exchange accomplished in the previous coproduction realms, this realm involves more thorough interaction with champion users. Collaborating on a case study or a service prototype development allows data providers and champion users to co-develop a tailored service, test its usability, and assess its added value. Only a service that proves useful and practical for users and that is tested with them can have a role in decision-making processes, meaningfully informing decisions that require consideration of past, current or future climate changes. Increasingly, hackathons and hackathon-like events, e.g. climathons are being used to stimulate creativity in problem-solving and enable co-development of new, shared knowledge. This part of the process is here termed “empowerment” and can include incremental or transformative learning. This process generates a sense of shared ownership since responsibilities are redistributed among all the participants. Finally, these newly built relationships can positively affect information-sharing and awareness-raising within collaborators’ networks.

17.

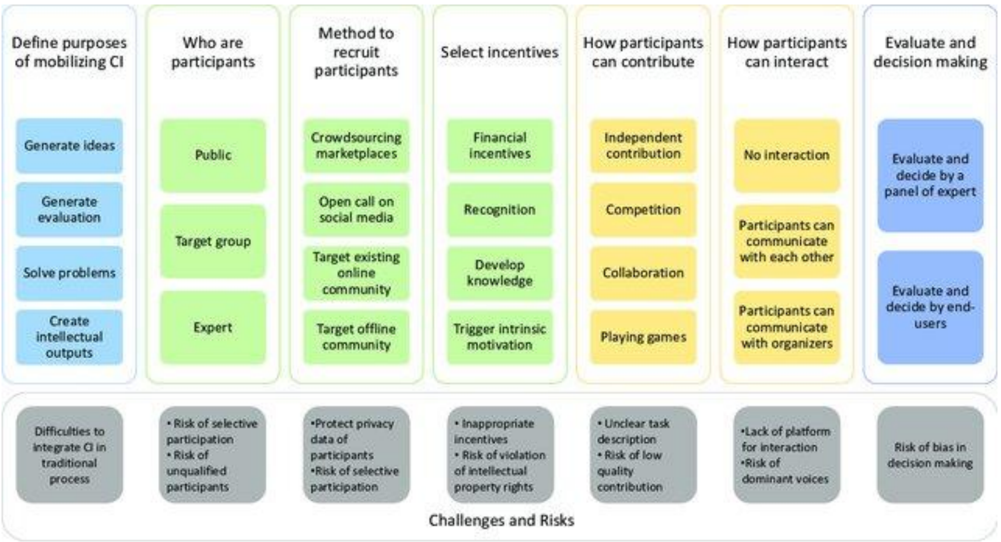

Name: Framework of process of mobilizing collective intelligence, by Nguyen et al. (2018)

Source: Google Images Search > [20]

Co-Creation Characteristics: Collective intelligence and decision-making

Description: The framework presents the types of participants, how participants contribute to projects, interactions among participants, and the evaluation of participants’ contributions and decision-making according to the different reasons for using collective intelligence. To generate an evaluation and solve problems, independent contributions were used often, with mostly no interaction among participants. In contrast, competition was often used to generate ideas, and participants were able to exchange ideas and receive feedback from each other. To create intellectual products, participants collaborated and were able to receive feedback from other participants and organizers to improve their products.

- Independent contribution of participants (collection): work is divided into small pieces; participants can work independently. There is a mechanism for aggregating contributions from all participants (e.g. averaging, voting).
- Competition: gives a well-defined problem to solve. Gives clear criteria for evaluation to recognize innovative ideas. Provides a strong communication plan before, during and after the competition. Uses different channels to publicize the competition in advance and provides real-time updates. Gives time to participants to understand the problem such as organizing an introduction workshop, providing a data set, and tutorials for training. Provides a forum for participants to exchange ideas and form their teams. Rewards for winners.
- Play games: Web-based, mobile-based applications accessible to a wide range of participants. Provides tutorials to participants. Creates different levels of complexity. Real-time updates and leaderboards are used to increase engagement from participants.
- Collaboration: Work is not able to be divided into independent pieces. Provides a platform for discussion, a way to record ideas from all participants (e.g. Wikis), and a moderator who supports the discussion. Provides tools to navigate ideas contributed by participants (e.g. text analysis) to identify patterns of ideas; automatic team matching.

18.

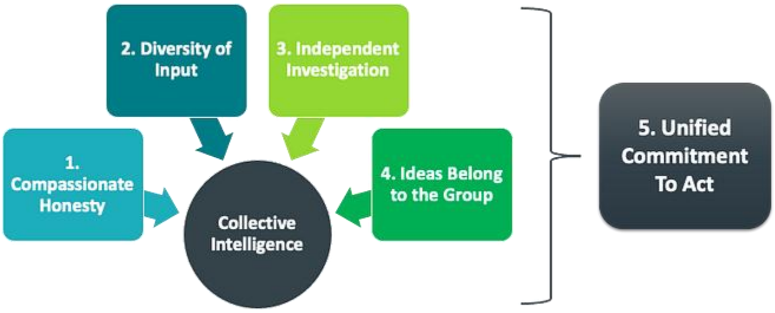

A Team Inventory: Five Capacities for Collective Intelligence

1. Compassionate Honesty
Truthfully and thoughtfully expressing our views, with courtesy and care.

Signs of Success	Practice More When…
✓ Many people openly express their views in meetings.<br>✓ When someone departs from team agreements (ex., interrupts or belittles someone else), another calls out the behavior and helps get the team back on track.<br>✓ Team members express gratitude and appreciation for those who offer their concerns.	⇒ Team meetings are heavily dominated by a few strong voices.<br>⇒ Team members who express their honest views are shunned by the group.<br>⇒ Team members avoid important and difficult conversations.

2. Diversity of Input
Seeking out perspectives that differ from our own, when trying to solve problems and come up with new ideas.

Signs of Success	Practice More When…
✓ Important conversations include diverse voices from a wide range of perspectives and backgrounds.<br>✓ Team members seek out the views of quieter members.<br>✓ Team members ask lots of questions, in order to understand the situation from many angels.	⇒ A few people from similar backgrounds, are making most of the decisions.<br>⇒ Team members are not asking questions of one another.

3. Independent Investigation
Investigating situations through the unique lens of our values, experiences, and set of data.

Signs of Success	Practice More When…
✓ Team members independently examine data before coming together to make important decisions.<br>✓ Teams utilize practices that support independent thinking, such as the consent process, reflection time, and/or breaking out into pairs for small group discussions.	⇒ Team members quickly conform their views to those of the group, instead of first forming their own opinion.<br>⇒ Decisions around the future, tend to look like decisions from the past.

4. Ideas Belong to the Group
Sharing ideas with detachment from our ego and contributing them fully to the group.

Signs of Success	Practice More When…
✓ Team members share ideas freely, and allow these ideas to be challenged, shaped, and molded by the group.<br>✓ Team members quickly embrace ideas from others when they are better than their own.<br>✓ After each view has been considered, we submit to the majority decision, and move the group forward.	⇒ Team members tend to cling to and defend their personal opinion.<br>⇒ Team members use possessive phrases like "my idea" or "your idea".

5. Unified Commitment to Act
Speaking and acting with unity, and whole-heartedly executing decisions.

Signs of Success	Practice More When…
✓ Team members communicate public support for decisions, even though they may have privately disagreed.<br>✓ Team members keep their commitments and act in accordance with team decisions.	⇒ Team members vocalize disagreement with group decisions after they are made.<br>⇒ Execution of decisions is falling short.

Name: Five Capacities for Collective Intelligence, Miller (2021)
Source: Google Images Search > [21]
Co-Creation Characteristics: Collective intelligence and decision-making
Description:

- Compassionate Honesty: Collective intelligence requires a commitment to discovering 'the best solution' or 'the truth'. Truth can only be discovered through honesty. In the absence of honesty, the best solutions and most creative ideas will remain hidden from us. When we hear a difficult yet honest message from someone who is uncaring and rude, it is easy to dismiss it. On the other hand, when the speaker is compassionate and caring, we are much more likely to hear what they have to say, explore that different perspective, and seek to understand even the most challenging suggestion.
- Diversity of Input: Research consistently shows that diverse groups of problem solvers consistently outperform homogenous groups — even when those homogeneous groups are made up of the best and brightest individuals.
- Independent investigations: Surprisingly, experiment after experiment, shows that humans tend to conform to the group, MORE than we tend to stand up for what we think individually. This phenomenon, known as "groupthink" has been noted as the cause of many decision-making disasters in the world, such as the Space Shuttle Challenger Disaster. At times, we might conform to the group due to fear of being different. At other times, it may be that we simply have not taken the time, done the research, nor given the topic enough thought to come up with our own independent view. Collective intelligence only arises when we first individually investigate the situation with our own set of data and from the lens of our own unique experience and understanding, and then come together to aggregate our collective view.

- Ideas belong to the group: When our ego is attached to our ideas, we work hard to defend those ideas — even when they are wrong. When, on the other hand, we contribute those ideas to the group, it is much easier to challenge them, test them, and mould them into something much better than the original. Just as fire is used to test and refine gold, similarly, testing, and challenging ideas, purifies and perfects them. Furthermore, if we believe in a "field of consciousness", then giving ideas to the group makes perfect sense. This concept maintains that there is a field, which is a source of inspiration and creativity, and it is equally available to all. Through developing our consciousness, we can more readily access this field. If ideas come from a field of consciousness, then they do not belong to us, as individuals. We are merely vessels, through which these ideas travel.
- Unified Commitment to Act: Action is the most important phase of decision-making. When we act with unified conviction, we generate a powerful force that supports our collective success. Furthermore, unified action enables us to clearly see the results of our decision, so that even when the idea is imperfect, we can continuously improve our decisions and actions. Sharing dissenting views after a decision is made, undermines the decision, weakens the team as a whole, and sets up the team for failure. Decision-making is meaningless if we fail to execute those decisions.

19. 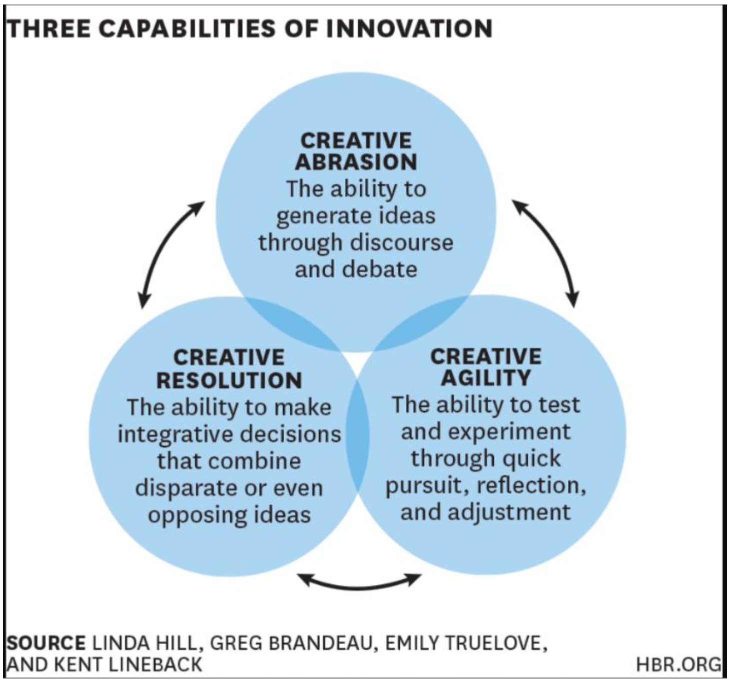

Name: Three capabilities of Innovation, Hill et al. (2015)
Source: Google Images Search > [22]
Co-Creation Characteristics: Collective creativity and decision-making
Description:

- Creative Abrasion: New and useful ideas emerge as people with diverse expertise, experience, or points of view thrash out their differences. The kind of collaboration that produces innovation is more than simple "get-along" cooperation. It involves and should involve passionate discussion and disagreement. To collaborate means making oneself vulnerable to hard questions and push-back. Not everyone wants to do that all the time. It's no wonder that some and perhaps many people choose to remain silent rather than participate.
- Creative Agility: Almost by definition, a truly creative solution is something that cannot be foreseen or planned. Thus, innovation is a problem-solving process that proceeds by trial and error. A portfolio of ideas is generated and tested, then revised, and retested, in an often

lengthy process of repeated experimentation. Instead of following some linear process that can be carefully planned in advance, it's messy and unpredictable. Organizations that innovate not only attempt new things, but they invite failure as part of the cost of discovery.

- Creative Resolution: Integrating ideas – incorporating the best of option A and option B to create something new, option C, that's better than A or B – often produces the most innovative solution. However, the process of integration can be inherently discomforting, emotionally, and intellectually. The problem – and the leadership challenge – arises because options A and B are often incompatible, even completely opposable, ideas. To arrive at option C means people must keep both A and B on the table, and that is difficult to do. When faced with two seemingly mutually exclusive alternatives, the human impulse is to choose one and discard the other as soon as possible, or to forge a simple compromise. It takes courage to hold open a multitude of possibilities long enough that new ways of combining them can emerge. There is often great pressure to make a choice, any choice, and move on. Innovative teams, however, know that integrative decision-making often involves more than simply and mechanically combining ideas. Rather, it requires a willingness to play with ideas and experiments until they "click." Discoveries emerge through constant iteration, through trying different approaches, including approaches that at first seemed inconsistent, through the involvement of lots of talented people, and through a willingness to wait and see what works and what doesn't.

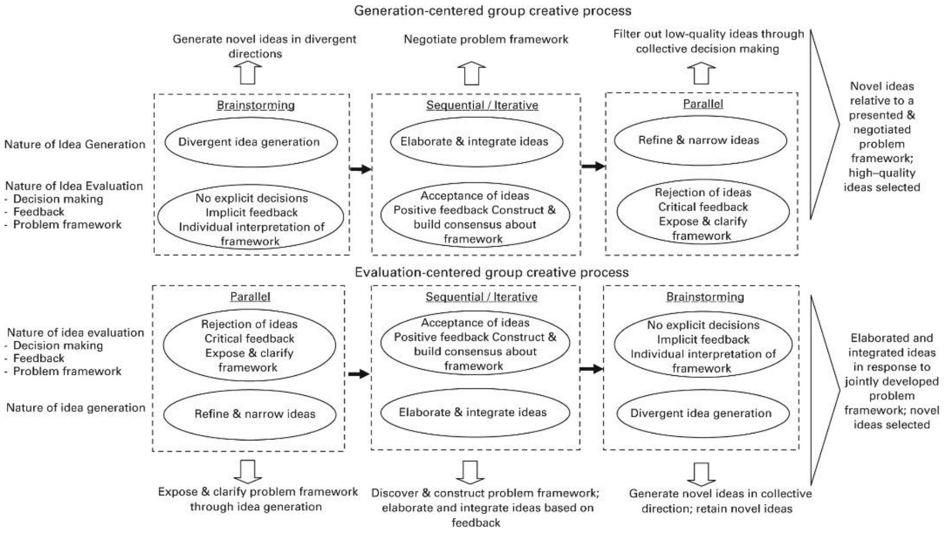

20.

Name: Alternative models of the group creative process, Harvey et al. (2013)
Source: Google Images Search > [23]
Co-Creation Characteristics: Collective creativity, collective intelligence, and decision-making
Description: Examining the 33 meeting segments revealed four different modes of interaction over creative ideas. In brainstorming mode, ideas were generated without evaluation; in sequential interactions, one idea was generated, elaborated, and evaluated; in parallel interactions, several ideas were generated and then evaluated simultaneously; and in iterative interactions, the group evaluated multiple ideas regarding group goals. Each mode involved different ways of evaluating and generating ideas. Groups did not engage in the four modes of interaction in the same sequence over time. Instead, we observed two broad ways that the modes of interactions were ordered.

- Brainstorming mode: In some cases, groups interacted in a way that closely resembled the traditional conception of idea generation. Brainstorming mode was characterized by group members generating ideas with little if any evaluation, relying on their own interpretation of the problem framework to do so. Decisions rarely occurred in this mode. Groups exchanged a great deal of information either before or during brainstorming, but information was rarely

used to elaborate or evaluate ideas. Groups also very rarely made decisions about ideas in brainstorming mode.

- Sequential mode: A second pattern was the sequential generation, discussion, and evaluation of one idea at a time. In this mode, groups elaborated on ideas and built consensus about the problem framework by considering the advantages and disadvantages of each idea. The sequential mode was the most productive. Ideas generated in this mode tended to be elaborations of existing ideas because members generally agreed with and built on a focal idea. Sequential discussion of new ideas therefore appeared to be a mechanism through which groups attended to and built on a single idea, rather than diverging in different directions. Sequential interactions also built consensus about the problem framework.
- Parallel mode: A third mode that emerged from the data was the parallel discussion of multiple ideas at the same time. In parallel mode, groups generated then compared and contrasted a small number of ideas, clarifying the problem framework and making decisions. Ideas generated in parallel mode tended to be alternatives to one another. Because ideas were compared with one another, the nature of idea generation was often to disagree and therefore to refine rather than build on ideas. These disagreements were task-based conflicts between group members. Their effect was to narrow the scope of ideas. This conflict did not prevent idea generation, however.
- Iterative mode: an iterative interaction in which groups introduced and discussed one idea, then introduced a new idea without directly comparing it with the previous idea, and then returned to the original idea. Ideas from earlier in the group discussion may have been re-introduced in this mode. This mode involved integrating ideas and shaping the problem framework in the process of making decisions. In iterative mode, group interactions are also built on and elaborated ideas, similar to sequential interactions, but by moving back and forth between ideas, groups also identified ways to integrate multiple ideas. This seemed to occur naturally, in response to additional information or others' ideas, rather than because the group was focused on a particular idea. Disagreements during this type of interaction tended to focus on a single idea, rather than the trade-offs between ideas. This contrasts with parallel discussions, in which group members argued that others' ideas, such as focusing on a specific disease category, were not possible. As in parallel and sequential interactions, ideas were likely to be decided on in iterative mode.

21. 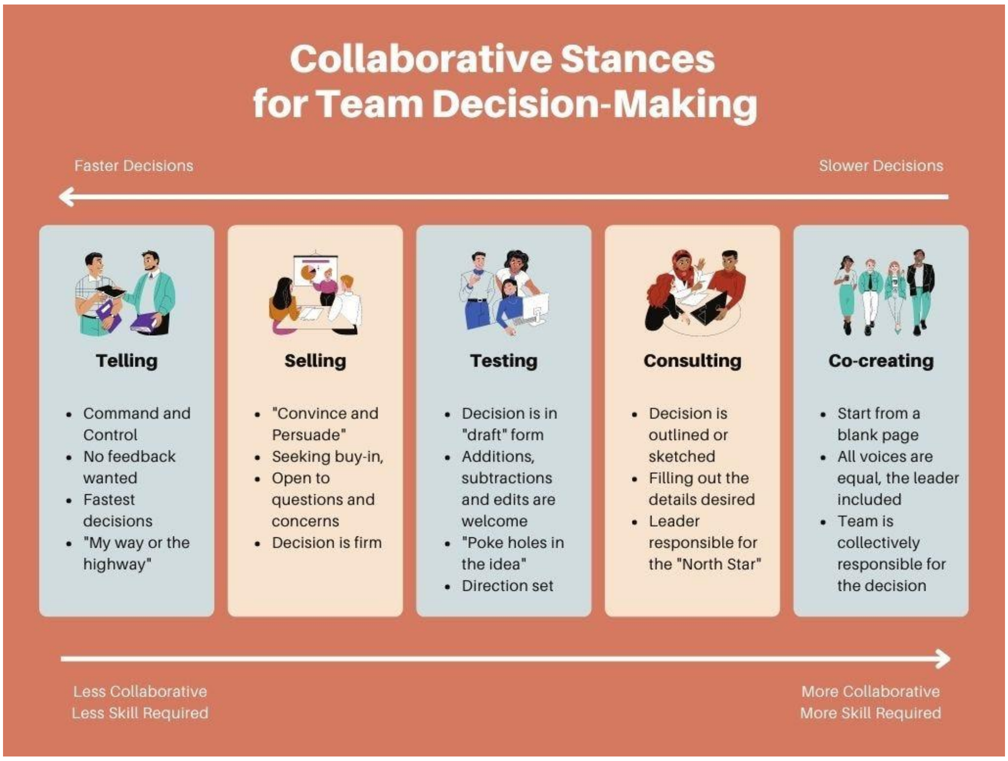

Name: Collaborative Stances for Team Decision-Making, by Penner (2022)
Source: Google Images Search > [24]
Co-Creation Characteristics: Decision-making

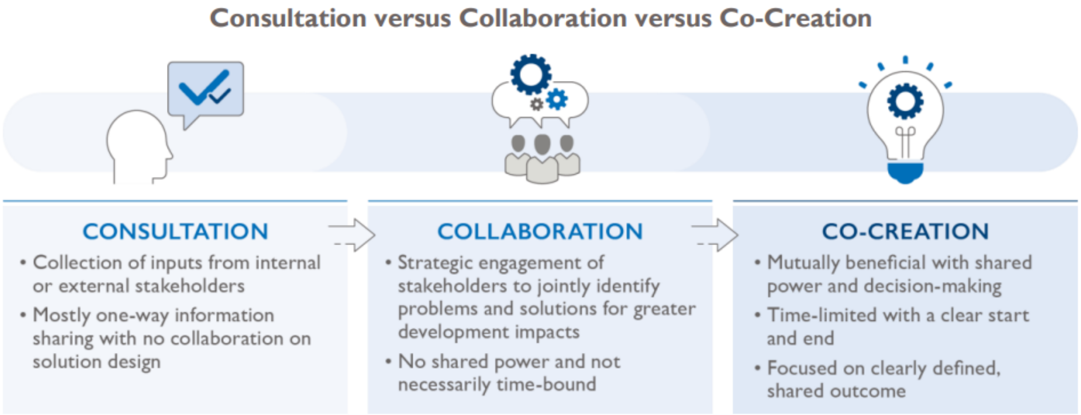

22.

Name: Consultation versus Collaboration versus Co-Creation
Source: Google Images Search > [25]
Co-Creation Characteristics: Empowerment and Decision-making
Description:

- Consultation: Collection of inputs from internal and external stakeholders. Mostly one-way information sharing with no collaboration on solution design.
- Collaboration: Strategic engagement of stakeholders to jointly identify problems and solutions for greater development impacts. No shared power and not necessarily time-bound.
- Co-Creation: Mutually beneficial with shared power and decision-making. Time-limited with a clear start and end. Focused on clearly defined, shared outcomes.

23.

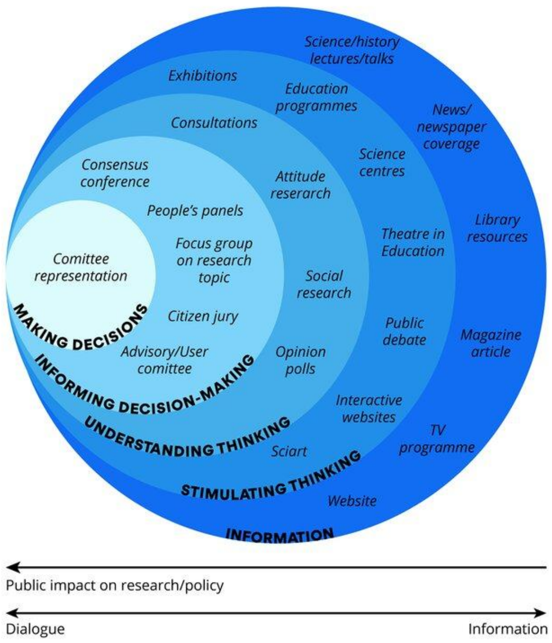

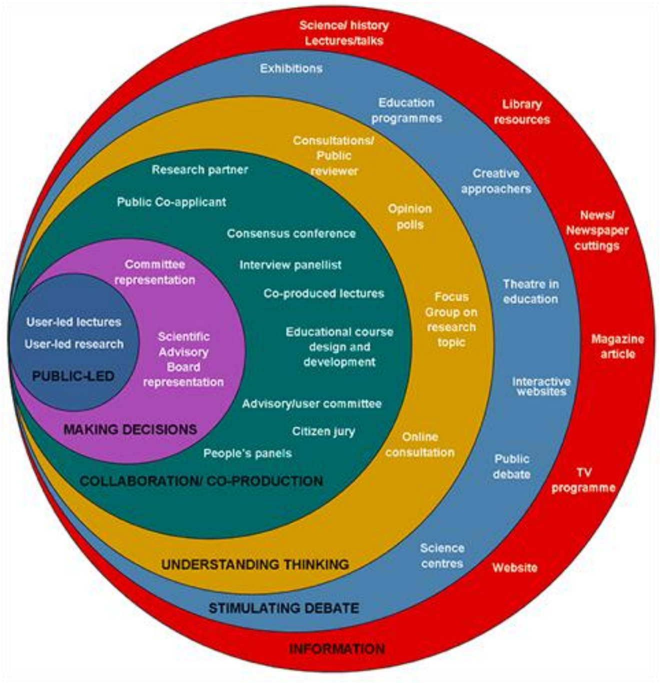

Name: Wellcome Trust's Public Engagement Onion
Source: Google Images Search > [8,26]
Co-Creation Characteristics: Participation and Decision-making
Description: Many of the more recent participation models, such as the Wellcome Trust's 'Onion', focus on public engagement with research (PER). This model shows how different activities can enable the publics to influence research and policy to different extents and is used by organisations such as Oxford University to inform their PER activity. The outer layer of the onion is made up of one-way interactions designed to share information, but as you peel away the sections the opportunities for dialogue grow until at the centre of the onion the power is transferred to the public.

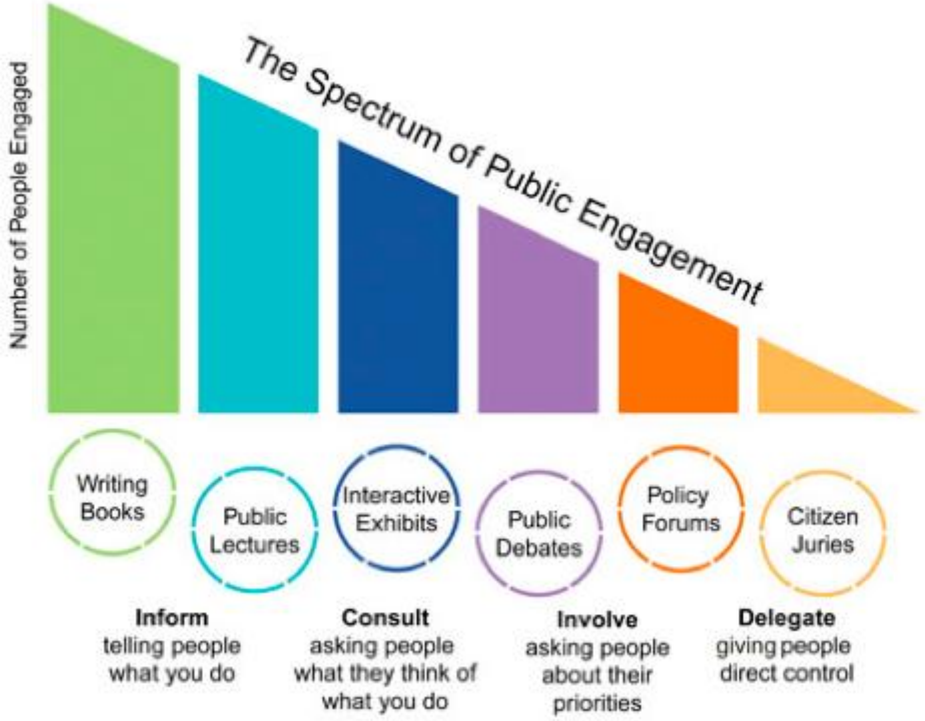

24.

Name: The Spectrum of Public Engagement, by Beltain
Source: Google Images Search > [26]
Co-Creation Characteristics: Participation and Decision-making
Description: Edinburgh University's Beltain Public Engagement network developed its own model which goes one step further to consider the number of people involved at each level. Shaped like a wedge, this model helps to show the value at both ends of the scale. Lighter touch activities only designed to inform can have a wider reach, while more intense projects with smaller groups can have a deeper impact.

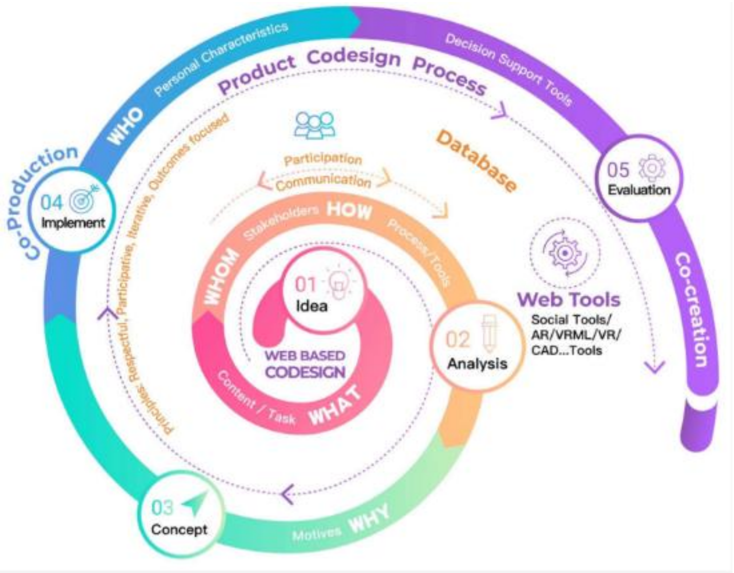

25.

Name: The framework of web-based co-design, by Kang et al. (2020)

Source: Google Images Search > [27]

Co-Creation Characteristics: Participation

Description: An online co-design project usually starts with the submission of an idea. The online product co-design process is divided into five main steps:

- Idea: An individual, team, or society comes up with an idea.
- Analysis: Co-planning and developing product strategies, gathering general and specific materials, and conducting data mining and social diffusion research.
- Concept: Open-innovation collaborative design, understanding and clearly defining the issue.
- Implement: Build prototypes and implement plans. It is enabled by digital production technologies. This step involves the management and delivery process of co-production.
- Evaluate: Testing the idea and improvements. Evaluation is not limited to the final product but is based on iterative and participatory relational processes.

References

1. Duarte AMB, Brendel N, Degbelo A, Kray C. Participatory Design and Participatory Research: An HCI Case Study with Young Forced Migrants. ACM Trans Comput-Hum Interact. 2018;25:3:1-3:39.

2. Ladder of Citizen Participation [Internet]. Organ. Engagem. 2019 [cited 2023 Sep 26]. Available from: https://organizingengagement.org/models/ladder-of-citizen-participation/

3. Eberson D. 360participation - » Models of participation [Internet]. [cited 2023 Oct 25]. Available from: https://360participation.com/models-of-participation/

4. Pretty JN. Participatory learning for sustainable agriculture. World Dev. 1995;23:1247–63.

5. Kimengsi J, Balgah R, Gwan A. Enhancing Community Participation for Rural Development in Central Ejagham of Cameroon: Challenges and Prospects. Int J Community Dev. 2016;4:20–32.

6. Dooris M, Heritage Z. Healthy Cities: Facilitating the Active Participation and Empowerment of Local People. J Urban Health. 2013;90:74–91.

7. Participation Models: Citizens, Youth, Online [Internet]. Creative Commons; 2012. Available from: https://www.nonformality.org/wp-content/uploads/2012/11/Participation_Models_20121118.pdf

8. Alex. Flow's Engagement Thresholds Model – Flow Associates [Internet]. [cited 2023 Oct 26]. Available from: https://flowassociates.com/2021/07/flows-engagement-thresholds-model/

9. Carter C. The Power and Pitfalls of Participatory Processes. 2023;

10. OECD. Citizens as Partners: Information, Consultation and Public Participation in Policy-Making [Internet]. Paris: Organisation for Economic Co-operation and Development; 2001 [cited 2023 Oct 25]. Available from: https://www.oecd-ilibrary.org/governance/citizens-as-partners_9789264195561-en

11. Stuart G. What is the Spectrum of Public Participation? [Internet]. Sustain. Community. 2017 [cited 2023 Oct 25]. Available from: https://sustainingcommunity.wordpress.com/2017/02/14/spectrum-of-public-participation/

12. Muronda B. A conceptual public participation framework for ward committees to promote local government democracy. 2017 [cited 2023 Oct 25]. Available from:

https://www.semanticscholar.org/paper/A-conceptual-public-participation-framework-for-to-Muronda/4bbbbdda44a7b3ae4979a7409fad2f9fb854ac1c

13. Cattaneo LB, Goodman LA. W(13) (PDF) What Is Empowerment Anyway? A Model for Domestic Violence Practice, Research, and Evaluation. Psychol Violence. 2014;5:84–94.

14. Rao R. True Empowerment [Internet]. Know- Innov. 2018 [cited 2023 Oct 25]. Available from: https://knowhowinnovations.com/true-empowerment/

15. Our Experience Model [Internet]. Late Nite Art. [cited 2023 Dec 15]. Available from: https://www.lateniteart.com/methodology

16. Ladder of Empowerment [Internet]. Organ. Engagem. 2019 [cited 2023 Oct 25]. Available from: https://organizingengagement.org/models/ladder-of-empowerment/

17. Rocha EM. A Ladder of Empowerment. J Plan Educ Res. 1997;17:31–44.

18. Steiner AA, Farmer J. Engage, participate, empower: Modelling power transfer in disadvantaged rural communities. Environ Plan C Polit Space. 2018;36:118–38.

19. Bojovic D, St. Clair AL, Christel I, Terrado M, Stanzel P, Gonzalez P, et al. Engagement, involvement and empowerment: Three realms of a coproduction framework for climate services. 2021;

20. Nguyen TV, Benchoufi M, Young B, Chall LE, Ravaud P, Boutron I. 63 Methods of mobilising collective intelligence through crowdsourcing in research:a scoping review. BMJ Evid-Based Med. 2018;23:A31–2.

21. Miller LM. FIVE CAPACITIES FOR UNLEASHING COLLECTIVE INTELLIGENCE: A TEAM INVENTORY [Internet]. Inst. Leadersh. Excell. LLC. 2021 [cited 2023 Oct 25]. Available from: https://www.instituteforleadershipexcellence.com/five-capacities-for-unleashing-collective-intelligence-a-team-inventory/

22. Hill LA, Brandeau G, Truelove E, Lineback K. The Capabilities Your Organization Needs to Sustain Innovation. Harv Bus Rev [Internet]. 2015 Jan 14 [cited 2023 Oct 25]; Available from: https://hbr.org/2015/01/the-capabilities-your-organization-needs-to-sustain-innovation

23. Harvey S, Kou C-Y. Collective Engagement in Creative Tasks: The Role of Evaluation in the Creative Process in Groups. Adm Sci Q. 2013;58:346–86.

24. Penner J. Collaborative Stances: A Decision-Making Framework for Leaders and Teams [Internet]. Soul Startups. 2022 [cited 2023 Oct 26]. Available from: https://medium.com/the-soul-of-startups/collaborative-stances-a-decision-making-framework-for-leaders-and-teams-a9ddfa4dd8a6

25. co-creation an interactive guide [Internet]. USAID; 2022. Available from: https://www.usaid.gov/sites/default/files/2022-12/Co-Creation_Toolkit_Interactive_Guide_-_March_2022_1.pdf

26. Costa GB, Smithyman R, O'Neill SL, Moreira LA. How to engage communities on a large scale? Lessons from World Mosquito Program in Rio de Janeiro, Brazil [Internet]. Gates Open Research; 2021 [cited 2023 Oct 26]. Available from: https://gatesopenresearch.org/articles/4-109

27. Kang X, Kang J, Chen W. Conceptualization and Research Progress on Web-Based Product Co-Design. Informatics. 2020;7:30.
