# Supplementary material for: Developing and validating the co-creation rainbow framework for intrinsic evaluation of methods: a health CASCADE structured review of models representing co-creation principles
Source: Health Res Policy Syst. 2025 Oct 10;23:127. doi: 10.1186/s12961-025-01381-1 (PMC12512873; doi:10.1186/s12961-025-01381-1)
Supplement: Supplementary file 3 — Additional file 3. Source models and features [file 12961_2025_1381_MOESM3_ESM.pdf]

Additional File 3. Source Models and Features

The table below illustrates which features of the source models were added to the integrated framework.

Source Model Name	Extracted Feature	Source Description of the Feature	Feature of the Co-Creation Rainbow Framework
1. Stage model of participation[1]	1. Power to make decisions	The power and control over decisions shift from researchers to co-researchers. At this level, practice- or community partners make important decisions essentially by themselves, while researchers assume the role of mere consultants.	Collective decision-making section; Empower Theme
2. Ladder of Citizen Participation[2]	2. Partnership	They agree to share planning and decision-making responsibilities through such structures as joint policy boards, planning committees, and mechanisms for resolving impasses.	Collective decision-making section
Ladder of Citizen Participation[2]	3. Delegated Power	Participation as delegated power occurs when public institutions, officials, or administrators give up at least some degree of control, management, decision-making authority	Collective decision-making section
3. IAP2 Spectrum of Public Participation[3]	4. Collaborate	To partner with the public in each aspect of the decision including the development of alternatives and the identification of the preferred solution. Examples: Citizen advisory committees, Consensus-building, and participatory decision-making.	Collective decision-making section

IAP2 Spectrum of Public Participation[4]	5. Inform	To provide the public with balanced and objective information to assist them in understanding the problem, alternatives, opportunities, and/or solutions. Examples: Fact sheets, websites, and open houses.	Informing section
IAP2 Spectrum of Public Participation[4]	6. Empower	To place final decision-making in the hands of the public. Examples: Citizen juries, ballots, and delegated decisions	Empower Theme; Collective decision-making section
IAP2 Spectrum of Public Participation[4]	7. Consult	To obtain public feedback on analysis, alternatives, and/or decisions. Examples: public comment, focus groups, surveys, and public meetings.	Understanding Section
4. Coproduction Framework[5]	8. Engagement realm	The framework engages stakeholders by raising awareness through different communication tools. Building awareness about existing initiatives and available knowledge is the first step in facilitating access	Engagement Theme
Coproduction Framework[5]	9. Involvement realm	To deepen our understanding of stakeholders' needs, perceptions, and rationales, we need a more intensive and meaningful exchange that can be achieved through interviews. Semi-structured interviews can encourage participants to speak freely and enable the emergence of new discourses and narratives.	Understanding Section
Coproduction Framework[5]	10. Empowerment realm	it empowers users who take part in their co-development. From the network of participants established in the previous steps, some stakeholders want to be more actively involved in problem analysis, exploring, and identifying possible as	Empower Theme

		well as preferred solutions. This process generates a sense of shared ownership, since responsibilities are redistributed among all the participants.	
Coproduction Framework[5]	11. Empowerment realm	Collaborating on a case study or a service prototype development allows data providers and champion users to co-develop a tailored service, test its usability and assess its added value.	Collaborating Section
5. Framework of process of mobilizing collective intelligence[6]	12. Collaboration	Work is not able to be divided into independent pieces. Provides a platform for discussion, a way to record ideas from all participants (e.g. Wikis), and a moderator who supports the discussion.	Collaborating Section
Framework of process of mobilizing collective intelligence[6]	13. Collaboration	Provides tools to navigate ideas contributed by participants (e.g. text analysis) to identify patterns of ideas; automatic team matching.	Analysis Category
Framework of process of mobilizing collective intelligence[6]	14. Independent contribution of participants	work is divided into small pieces; participants can work independently. There is a mechanism for aggregating contributions from all participants	Individual to Collective Spectrum
6. Five Capacities for Collective Intelligence[7]	15. Independent investigations	"groupthink" has been noted as the cause of many decision-making disasters in the world. Collective intelligence only arises when we first individually investigate the situation with our own set of data and from the lens of our own unique experience and understanding, and then come together to aggregate our collective view.	Individual to Collective Spectrum
Five Capacities for Collective Intelligence[7]	16. Unified Commitment to Act	Action is the most important phase of decision-making. Unified action enables us to	Individual to Collective Spectrum;

		clearly see the results of our decision, so that even when the idea is imperfect, we can continuously improve our decisions and actions.	Collective decision-making section
7. Three capabilities of Innovation[8]	17. Creative Abrasion	New and useful ideas emerge as people with diverse expertise, experience, or points of view thrash out their differences. The kind of collaboration that produces innovation is more than simple "get-along" cooperation. It involves and should involve passionate discussion and disagreement. To collaborate means making oneself vulnerable to hard questions and push-back.	Individual to Collective Spectrum; Collaborating Section
Three capabilities of Innovation[8]	18. Creative Resolution	There is often great pressure to make a choice, any choice, and move on. Innovative teams, however, know that integrative decision-making often involves more than simply and mechanically combining ideas. Rather, it requires a willingness to play with ideas and experiments until they "click." Discoveries emerge through constant iteration through the involvement of lots of talented people, and through a willingness to wait and see what works and what does not.	Individual to Collective Spectrum; Collective decision-making section
8. Alternative models of the group creative process[9]	19. Brainstorming mode	Brainstorming mode was characterized by group members generating ideas with little if any evaluation, relying on their own interpretation of the problem framework to do so. Decisions rarely occurred in this mode. Groups exchanged a great deal of information either before or	Stimulating Section

		during brainstorming, but information was rarely used to elaborate or evaluate ideas	
Alternative models of the group creative process[9]	20. Sequential mode	In this mode, groups elaborated on ideas and built consensus about the problem framework by considering the advantages and disadvantages of each idea. Sequential discussion of new ideas therefore appeared to be a mechanism through which groups attended to and built on a single idea, rather than diverging in different directions.	Collaborating Section
9. Consultation versus Collaboration versus Co-Creation[10]	21. Consultation	Collection of inputs from internal and external stakeholders. Mostly one-way information sharing with no collaboration on solution design.	Understanding Section
Consultation versus Collaboration versus Co-Creation[10]	22. Collaboration	Strategic engagement of stakeholders to jointly identify problems and solutions for greater development impacts. No shared power.	Collaborating Section
Consultation versus Collaboration versus Co-Creation[10]	23. Co-Creation	Mutually beneficial with shared power and decision-making. Time-limited with a clear start and end. Focused on clearly defined, shared outcomes.	Collective decision-making section
10. Wellcome Trust's Public Engagement Onion[11]	24. Information	The first layer includes: a website, TV programme, magazine article, library resources, science/history lectures/talks, and magazine articles.	Informing Section
Wellcome Trust's Public Engagement Onion[11]	25. Stimulating Debate	The second layer includes: Science centres, public debate, interactive websites, theatre in education, creative approaches, education programmes, and exhibitions.	Stimulating Section
Wellcome Trust's Public	26. Understanding Thinking	The third layer includes: online consultation, focus	Understanding Section

Engagement Onion[11]		group on research topic, opinion polls, consultation/public reviewer.	
Wellcome Trust's Public Engagement Onion[11]	27. Collaboration/Co-Production	The fourth layer includes: People's panels, citizen jury, advisory/user committees, education course design and development, co-produced lectures, consensus conference, etc.	Collaborating Section
Wellcome Trust's Public Engagement Onion[11]	28. Making Decisions	The fifth layer includes: the Scientific Advisory Board representative, and Committee representative.	Collective decision-making section
11. The Spectrum of Public Engagement[12]	29. Inform	Telling people what you do. Example: writing books or public lectures. Involves the most people.	Informing Section
The Spectrum of Public Engagement[12]	30. Consult	Asking people what they think of what you do. Example: Interactive Exhibits. This involves fewer people than inform.	Understanding Section
12. An Effective Community Participation Model[13]	31. Stage 1: Information, Education and Planning (IEP) Stage	Provides adequate and timely information, educating people about the development initiatives and outlining a plan of action which is critical in generating a process of participation.	Informing Section
An Effective Community Participation Model[13]	32. Stage 4: Feedback Stage of Participation	Includes consultations with local people to assess their needs and evaluate the outcome of development projects, and hold local people accountable for successes and failures.	Understanding Section
13. Wheel of Participation[14]	33. Good Quality Information	Providing information that the community wants and/or needs; e.g. discussion papers for development plans. Example techniques: Leaflets and exhibitions for planned projects.	Informing Section
Wheel of Participation[14]	34. Genuine Consultation	Government/agency actively discussing issues and current thinking with community/public before	Understanding Section

		taking action; e.g. liaison with interest groups. Example techniques: Citizen panels, Focus groups, User panels.	
Wheel of Participation[14]	35. Partnership	Solving problems in partnership with the community; e.g. formal partnership. Example techniques: co-option, stakeholder groups, and design game.	Collaborating Section
Wheel of Participation[14]	36. Delegated Control	Delegating limited decision-making powers in a particular area or project	Collective decision-making section
14. Analytical framework for conducting comparative surveys and country case Studies[15]	37. Consultation	Consultation is a two-way relationship in which citizens provide feedback to the government. It is based on the prior definition by the government of the issue on which citizens' views are being sought and requires the provision of information. Governments define the issues for consultation, set the questions and manage the process, while citizens are invited to contribute their views and opinions. Examples: public opinion surveys, comments on draft legislation.	Understanding Section
15. Know How Innovations' Empowerment Model[16]	38. True Empowerment (More Knowledge – More Authority)	Progressively greater knowledge-backed authority fuels true empowerment. This workforce possesses the knowledge to make sound decisions, actuated by their authority for relevant action. The inclusive yet independent culture creates a sense of ownership among the employees.	Empower Theme
16. Ladder of Empowerment[17]	39. Mediated Empowerment	Is a highly professionalized model in which the process of empowerment is mediated by an expert or professional. The locus in this type can either be the individual or	Empower Theme; Collective decision-making section

		community. Its goals are to provide knowledge and information necessary for individual or community decision making and action.	
17. Engage, Participate and Empower Model[18]	40. Engage	Platform for community engagement, and trigger of community participation	Engage Theme
18. The framework of web-based co-design[19]	41. Analysis	Gathering the general and specific materials and conducting data mining and social diffusion research.	Analysis Category
19. Typology of Participation[20]	42. Functional Participation	not real power-sharing; looks good but decisions are taken externally.	Participate Theme
20. Continuum of Public Participation[21]	43. Collaboration	Representatives of the public are actively involved in developing solutions and directly influencing decisions. This usually involved iterative activities, dialogue, and in-depth working relationships with more focus on joint responsibilities. Examples: round tables, workshops, and public advisory committees.	Participate Theme; Collaborating Section
[1] [1]<br>[2][2]<br>[3][3,4]<br>[4][3,4]<br>[5][5]<br>[6][6]<br>[7][7]<br>[8][8]<br>[9][9]<br>[10][10]<br>[11][11,12]<br>[12][12]<br>[13][13]<br>[14][3,11,14,15]<br>[15][3,16]<br>[16][17]<br>[17][18,19]<br>[18][20]<br>[19][21]<br>[20][22,23]<br>[21][24]			

References

1. Duarte AMB, Brendel N, Degbelo A, Kray C. Participatory Design and Participatory Research: An HCI Case Study with Young Forced Migrants. ACM Trans Comput-Hum Interact. 2018;25:3:1-3:39.

2. Ladder of Citizen Participation [Internet]. Organ. Engagem. 2019 [cited 2023 Sep 26]. Available from: https://organizingengagement.org/models/ladder-of-citizen-participation/

3. Participation Models: Citizens, Youth, Online [Internet]. Creative Commons; 2012. Available from: https://www.nonformality.org/wp-content/uploads/2012/11/Participation_Models_20121118.pdf

4. Stuart G. What is the Spectrum of Public Participation? [Internet]. Sustain. Community. 2017 [cited 2023 Oct 25]. Available from: https://sustainingcommunity.wordpress.com/2017/02/14/spectrum-of-public-participation/

5. Bojovic D, St. Clair AL, Christel I, Terrado M, Stanzel P, Gonzalez P, et al. Engagement, involvement and empowerment: Three realms of a coproduction framework for climate services. 2021;

6. Nguyen TV, Benchoufi M, Young B, Chall LE, Ravaud P, Boutron I. 63 Methods of mobilising collective intelligence through crowdsourcing in research:a scoping review. BMJ Evid-Based Med. 2018;23:A31–2.

7. Miller LM. FIVE CAPACITIES FOR UNLEASHING COLLECTIVE INTELLIGENCE: A TEAM INVENTORY [Internet]. Inst. Leadersh. Excell. LLC. 2021 [cited 2023 Oct 25]. Available from: https://www.instituteforleadershipexcellence.com/five-capacities-for-unleashing-collective-intelligence-a-team-inventory/

8. Hill LA, Brandeau G, Truelove E, Lineback K. The Capabilities Your Organization Needs to Sustain Innovation. Harv Bus Rev [Internet]. 2015 Jan 14 [cited 2023 Oct 25]; Available from: https://hbr.org/2015/01/the-capabilities-your-organization-needs-to-sustain-innovation

9. Harvey S, Kou C-Y. Collective Engagement in Creative Tasks: The Role of Evaluation in the Creative Process in Groups. Adm Sci Q. 2013;58:346–86.

10. co-creation an interactive guide [Internet]. USAID; 2022. Available from: https://www.usaid.gov/sites/default/files/2022-12/Co-Creation_Toolkit_Interactive_Guide_-_March_2022_1.pdf

11. Alex. Flow's Engagement Thresholds Model – Flow Associates [Internet]. [cited 2023 Oct 26]. Available from: https://flowassociates.com/2021/07/flows-engagement-thresholds-model/

12. Costa GB, Smithyman R, O'Neill SL, Moreira LA. How to engage communities on a large scale? Lessons from World Mosquito Program in Rio de Janeiro, Brazil [Internet]. Gates Open

Research; 2021 [cited 2023 Oct 26]. Available from: https://gatesopenresearch.org/articles/4-109

13. Kimengsi J, Balgah R, Gwan A. Enhancing Community Participation for Rural Development in Central Ejagham of Cameroon: Challenges and Prospects. Int J Community Dev. 2016;4:20–32.

14. Dooris M, Heritage Z. Healthy Cities: Facilitating the Active Participation and Empowerment of Local People. J Urban Health. 2013;90:74–91.

15. Carter C. The Power and Pitfalls of Participatory Processes. 2023;

16. OECD. Citizens as Partners: Information, Consultation and Public Participation in Policy-Making [Internet]. Paris: Organisation for Economic Co-operation and Development; 2001 [cited 2023 Oct 25]. Available from: https://www.oecd-ilibrary.org/governance/citizens-as-partners_9789264195561-en

17. Rao R. True Empowerment [Internet]. Know- Innov. 2018 [cited 2023 Oct 25]. Available from: https://knowhowinnovations.com/true-empowerment/

18. Ladder of Empowerment [Internet]. Organ. Engagem. 2019 [cited 2023 Oct 25]. Available from: https://organizingengagement.org/models/ladder-of-empowerment/

19. Rocha EM. A Ladder of Empowerment. J Plan Educ Res. 1997;17:31–44.

20. Steiner AA, Farmer J. Engage, participate, empower: Modelling power transfer in disadvantaged rural communities. Environ Plan C Polit Space. 2018;36:118–38.

21. Kang X, Kang J, Chen W. Conceptualization and Research Progress on Web-Based Product Co-Design. Informatics. 2020;7:30.

22. Eberson D. 360participation -» Models of participation [Internet]. [cited 2023 Oct 25]. Available from: https://360participation.com/models-of-participation/

23. Pretty JN. Participatory learning for sustainable agriculture. World Dev. 1995;23:1247–63.

24. Muronda B. A conceptual public participation framework for ward committees to promote local government democracy. 2017 [cited 2023 Oct 25]. Available from: https://www.semanticscholar.org/paper/A-conceptual-public-participation-framework-for-to-Muronda/4bbbbdda44a7b3ae4979a7409fad2f9fb854ac1c
