# Supplementary material for: Developing and validating the co-creation rainbow framework for intrinsic evaluation of methods: a health CASCADE structured review of models representing co-creation principles
Source: Health Res Policy Syst. 2025 Oct 10;23:127. doi: 10.1186/s12961-025-01381-1 (PMC12512873; doi:10.1186/s12961-025-01381-1)
Supplement: Supplementary file 4 — Additional file 4. Detailed Results on Phase 1 [file 12961_2025_1381_MOESM4_ESM.pdf]

Additional File 4. Detailed Results on Phase 1

Results

Phase 1: Framework Development

Step 1: Screening and Testing Existing Models

From an initial 1,200 hits, 1,117 models were excluded based on selection criteria 1, leaving 83 models for inclusion (see Table 5 for details on search terms and results).

Table 1. *Model and framework search strategy and results*

**Search term 1**	**Search term 2**	**Search term 3**	**Screened Models**	**Included Models**
Model	Participation	—	100	23
Framework	Participation	—	100	5
Model	Empowerment	—	100	23
Framework	Empowerment	—	100	1
Model	Collective Intelligence	—	100	5
Framework	Collective Intelligence	—	100	5
Model	Collective Creativity	—	100	4
Framework	Collective Creativity	—	100	4
Model	Decision-making	Co-Creation[1]	100	6
Framework	Decision-making	Co-Creation	100	0
Model	Methods	Co-Creation	100	4
Framework	Methods	Co-Creation	100	3
TOTAL INCLUDED MODELS				83

Of these 83 models, 25 met selection criteria 2 and proceeded to the testing phase. An additional file provides detailed information on these models, including their sources, descriptions, and co-creation principles [see Additional File 1]. Testing the models revealed that five did not offer sufficient structure or guidance to support the classification of methods and were therefore excluded. Figure 1 shows the process of model inclusion, resembling a PRISMA-like flow chart. Figure 2 illustrates the included models and their associated principles, and an additional file contains the completed PRISMA checklist [see Additional File 2].

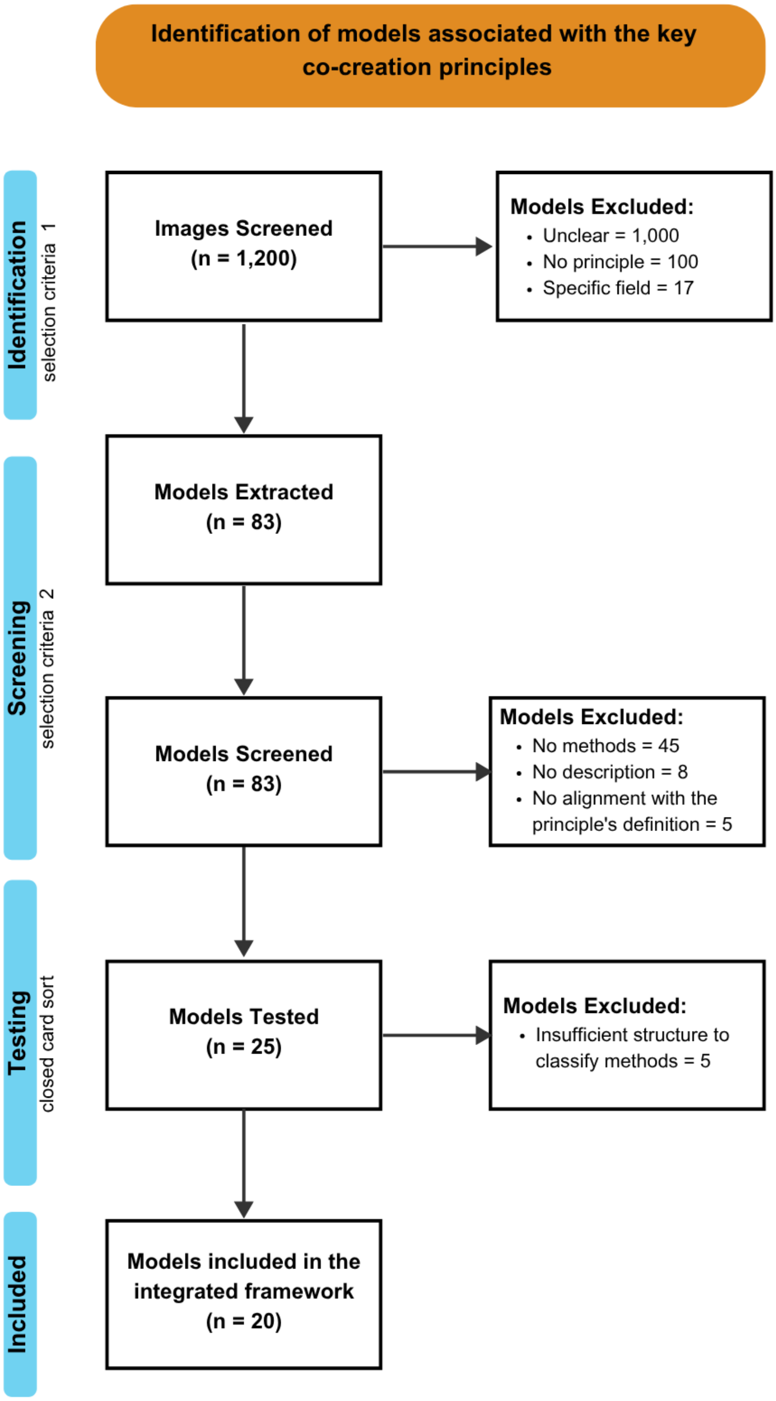

Figure 1*. PRISMA-like flow chart of models and frameworks. Visualize the steps from identification to the inclusion of the final set of 20 models associated with the co-creation principles.*

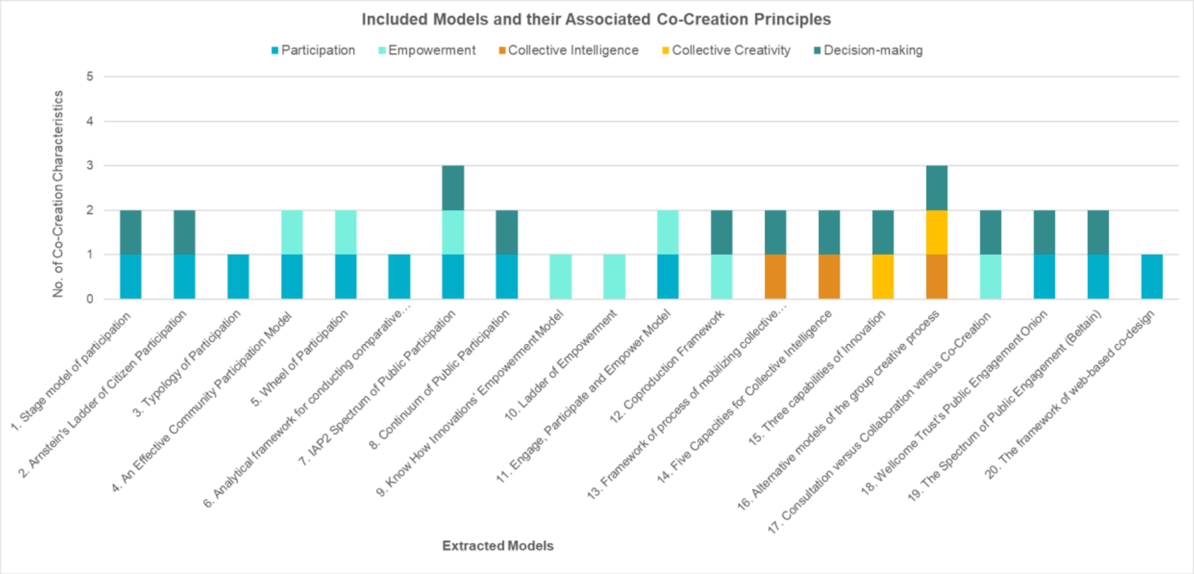

Figure 2. *Included models and their associated co-creation principles. The graph represented the distribution of co-creation principles to the 20 included models.*

Of the 20 models included in the study, 60% (12/20) feature participation, 60% (12/20) include decision-making, 40% (8/20) address empowerment, 15% (3/20) involve collective creativity, and 10% (2/20) incorporate collective intelligence. The models with the most principles are the IAP2 Spectrum of Public Participation (covering participation, empowerment, and decision-making) and the Alternative Models of the Group Creative Process (covering collective intelligence, collective creativity, and decision-making).

Thirteen models exhibit two co-creation principles: five models combine participation and decision-making, three combine participation and empowerment, two combine empowerment and decision-making, two combine collective intelligence and decision-making, and one combines collective creativity and decision-making. Additionally, there are models with a single co-creation principle: with three models focused solely on participation and two on empowerment.

Step 2: Hybrid Framework Development

These 20 included models' key features were integrated into the hybrid framework. An additional file contains details about which features of the source models were added to the integrated framework [see Additional File 3]. The Co-Creation Rainbow framework integrates the strengths and features of 20 distinct models to form a robust hybrid tool for evaluating whether methods uphold key co-creation principles.

The name "Co-Creation Rainbow" was chosen to reflect the framework's role in examining the spectrum of methods and to symbolise the diverse range of co-creation principles it encompasses. The term 'rainbow' signifies the diverse characteristics and positions of methods within the framework, serving as a visual metaphor that underscores that no single point holds more significance than another. Instead, the framework is designed to discern how methods contribute to the spectrum of co-creation principles. This name highlights the

concept that co-creation can manifest in diverse forms and intensities, providing a systematic approach to understanding and assessing methods used in the co-creation process.
