# Supplementary material for: Developing and validating the co-creation rainbow framework for intrinsic evaluation of methods: a health CASCADE structured review of models representing co-creation principles
Source: Health Res Policy Syst. 2025 Oct 10;23:127. doi: 10.1186/s12961-025-01381-1 (PMC12512873; doi:10.1186/s12961-025-01381-1)
Supplement: Supplementary file 5 — Additional file 5. The co-Creation Rainbow Framework: card sorting outcome report [file 12961_2025_1381_MOESM5_ESM.pdf]

Additional File 5. The Co-Creation Rainbow framework: Card Sorting Outcome Report

Definitions

- **Agreement rate** = The agreement rate is the percentage of users agreeing that a card belongs in this category.
- **Category** = the section of the hybrid
- **Card** = the method to be sorted into the category

Card Sorting Outcomes

Sorted Cards (Methods):

These cards had an agreement rate of 50% or more, placing them into a distinct section of the framework. Therefore, these methods are added to the Co-Creation Rainbow framework at this stage.

Table S1. Sorted Cards

Number	Sorted Cards (method name)	Section	Agreement rate (%)	Sourced from Grey or Academic Lit.
1.	Pie Chart Agenda	Informing	90.91	Grey
2.	Operation Manual(S)	Informing	90.91	Grey
3.	Audiovisual Patient Information	Informing	81.82	Academic
4.	Pecha Kucha / Ignite	Informing	72.73	Grey
5.	RACI Matrix	Informing	63.64	Grey
6.	Train The Trainer / Train-The-Trainer	Informing	63.64	Academic
7.	Video Prototyping	Informing	63.64	Grey
8.	IDOARRT Meeting Design	Informing	54.44	Grey
9.	Ad-Hoc User Acceptance Questionnaire	Understanding	100	Academic
10.	Capability Opportunity Motivation And Behaviour (COM-B) Self-Evaluation Questionnaire	Understanding	100	Academic
11.	Cherokee Self-Reliance Questionnaire	Understanding	100	Academic
12.	Community-Based Survey / Community Based Survey	Understanding	100	Academic
13.	Daily Diaries / Daily Diary Method	Understanding	100	Academic
14.	Direct Observation(S)	Understanding	100	Academic
15.	Ethnographic Interviews	Understanding	100	Academic
16.	Ethnographic Observation	Understanding	100	Academic
17.	Evidence-Based Practice Questionnaire	Understanding	100	Academic
18.	Expert Interviews	Understanding	100	Academic
19.	Exploratory Interviews	Understanding	100	Academic
20.	Face-To-Face Interviews / Face-To-Face Dialogue / In-Person Interviews	Understanding	100	Academic

21.	Health Literacy Questionnaire	Understanding	100	Academic
22.	Health Questionnaire	Understanding	100	Academic
23.	Health Survey	Understanding	100	Academic
24.	In-Depth Interviews	Understanding	100	Academic
25.	Individual Interviews	Understanding	100	Academic
26.	Key Informant Interviews	Understanding	100	Academic
27.	Narrative Interviews	Understanding	100	Academic
28.	Non-Participant Observation	Understanding	100	Academic
29.	Observational Study	Understanding	100	Academic
30.	One-To-One Interviews	Understanding	100	Academic
31.	Online Questionnaire / Internet Questionnaire / Web-Based Questionnaire	Understanding	100	Academic
32.	Open-Ended Interview(S)	Understanding	100	Academic
33.	Open-Ended Questionnaire / Open Ended Questionnaire	Understanding	100	Academic
34.	Participant Observation	Understanding	100	Academic
35.	Participatory Observation(S)	Understanding	100	Academic
36.	Picker Patient Experience Questionnaire	Understanding	100	Academic
37.	Psychiatric Inpatient Experience Questionnaire	Understanding	100	Academic
38.	Psychiatric Out-Patient Experiences Questionnaire (Popeq)	Understanding	100	Academic
39.	Qualitative Interviews	Understanding	100	Academic
40.	Qualitative Survey / Qualitative Questionnaire	Understanding	100	Academic
41.	Quality Involvement Questionnaire	Understanding	100	Academic
42.	Quality Of Life Questionnaire (QOLQ)	Understanding	100	Academic
43.	Quantitative Interviews / Standardized Interviews	Understanding	100	Academic
44.	Quantitative Survey	Understanding	100	Academic
45.	Semi-Structured Interviews / Semistructured Interviews / Semi-Structured Dialogues	Understanding	100	Academic
46.	Stakeholder Values Questionnaire	Understanding	100	Academic
47.	Stakeholder Interviews	Understanding	100	Academic
48.	Structured Interviews	Understanding	100	Academic
49.	Students International Physical Activity Questionnaire	Understanding	100	Academic
50.	Telephone Interview(S) / Phone Interview	Understanding	100	Academic
51.	Telephone Survey /Computer-Assisted Telephone Interviewing (CATI)	Understanding	100	Academic
52.	Therapeutic Engagement Questionnaire	Understanding	100	Academic
53.	Travel Diaries / Travel Journals	Understanding	100	Academic
54.	Unstructured Interviews	Understanding	100	Academic

55.	Virtual Reality Symptoms Questionnaire	Understanding	100	Academic
56.	Working Alliance Inventory – Short Revised (Wai-Sr)	Understanding	100	Academic
57.	Bespoke Questionnaire	Understanding	100	Academic
58.	Close Ended Questionnaire	Understanding	100	Academic
59.	Cross-Sectional Observations	Understanding	100	Academic
60.	Elicitation Interview	Understanding	100	Academic
61.	Evaluation Questionnaire	Understanding	100	Academic
62.	Exit Interview(S)	Understanding	100	Academic
63.	Feedback Questionnaire	Understanding	100	Academic
64.	Field Notes / Ethnographic Field Notes / Observations Field Notes / Qualitative Field Notes / Personal Field Notes	Understanding	100	Academic
65.	Field Observation(S)	Understanding	100	Academic
66.	Geo-Questionnaire	Understanding	100	Academic
67.	Interactive Interviewing	Understanding	100	Academic
68.	Intercept Interviews	Understanding	100	Academic
69.	Interviewer-Administered Questionnaire	Understanding	100	Academic
70.	Patient Narratives / Patient Experience Narratives	Understanding	100	Academic
71.	Patient Satisfaction Questionnaire	Understanding	100	Academic
72.	Personal Diaries / Customer Diaries	Understanding	100	Academic
73.	Postintervention Questionnaires	Understanding	100	Academic
74.	Practical Observations	Understanding	100	Academic
75.	Satisfaction Questionnaire	Understanding	100	Academic
76.	Self-Administered Questionnaire	Understanding	100	Academic
77.	Self-Reporting Questionnaire (SRQ)	Understanding	100	Academic
78.	Semistructured Questionnaire / Semi-Structured Questionnaire	Understanding	100	Academic
79.	Standardized Questionnaire	Understanding	100	Academic
80.	Structured Questionnaire	Understanding	100	Academic
81.	Systematic Observation	Understanding	100	Academic
82.	Usability Questionnaire	Understanding	100	Academic
83.	User Narratives / Life Journey Narratives	Understanding	100	Academic
84.	Videotaped Observations	Understanding	100	Academic
85.	Community Consultation	Understanding	90.91	Academic
86.	Convergent Interviews / Convergent Interviewing	Understanding	90.91	Academic
87.	Cross-Sectional Survey	Understanding	90.91	Academic
88.	Environmental Survey	Understanding	90.91	Academic
89.	Experiential Observation	Understanding	90.91	Academic
90.	Expert Consultation	Understanding	90.91	Academic
91.	Motivational Interviewing	Understanding	90.91	Academic
92.	Patient Diaries	Understanding	90.91	Academic
93.	People Shadowing	Understanding	90.91	Grey

94.	Practice Environment Scale Of The Nursing Work Index (PES-NWI)	Understanding	90.91	Academic
95.	Questionnaire Survey / Questionnaire Surveys	Understanding	90.91	Academic
96.	Reflective Diaries	Understanding	90.91	Academic
97.	Scenario-Based Interviews / Situational Interviews	Understanding	90.91	Grey
98.	Second-Order Observation	Understanding	90.91	Academic
99.	Walk-Along Interviews / Go-Alongs / Walking Interviews	Understanding	90.91	Academic
100.	Contextual Observation	Understanding	90.91	Grey
101.	Contingent Valuation	Understanding	90.91	Academic
102.	Naturalistic Observations	Understanding	90.91	Academic
103.	Peers Observing Peers	Understanding	90.91	Grey
104.	Personal Narratives (PN)	Understanding	90.91	Academic
105.	Pictogram Interview	Understanding	90.91	Grey
106.	Repertory Grid Technique	Understanding	90.91	Academic
107.	Self-Assessment Questionnaire	Understanding	90.91	Grey
108.	Critical Incident Technique (CIT)	Understanding	81.82	Academic
109.	Day-In-The-Life Mapping	Understanding	81.82	Grey
110.	Emotional Touchpoints	Understanding	81.82	Academic
111.	Mental Mapping	Understanding	81.82	Grey
112.	Participatory Survey	Understanding	81.82	Academic
113.	Reflection Notes	Understanding	81.82	Academic
114.	Stakeholder Consultation	Understanding	81.82	Academic
115.	Insights And Learnings	Understanding	81.82	Grey
116.	Narrative Ethnography	Understanding	81.82	Academic
117.	Narrative Interview	Understanding	81.82	Academic
118.	Pathographies	Understanding	81.82	Academic
119.	Reflective Field Notes / Reflexive Field Notes	Understanding	81.82	Academic
120.	Reflective Narratives	Understanding	81.82	Academic
121.	Cognitive Mapping	Understanding	72.73	Academic
122.	Neighborhood Mapping	Understanding	72.73	Academic
123.	Q-Methodology	Understanding	72.73	Academic
124.	Structured Interview Matrix Facilitation Technique / Interview Matrix (SIM)	Understanding	72.73	Academic
125.	We Saw, We Heard	Understanding	72.73	Grey
126.	Behavioural Observation	Understanding	72.73	Grey
127.	Case Study Method	Understanding	72.73	Academic
128.	Conversation Capture	Understanding	72.73	Grey
129.	Explanatory Narratives	Understanding	72.73	Academic
130.	Pain-Gain Map	Understanding	72.73	Grey
131.	Photojournal	Understanding	72.73	Grey
132.	Reference Project / Case Studies	Understanding	72.73	Grey
133.	Self-Assessment Method	Understanding	72.73	Academic
134.	Show Me Your Values	Understanding	72.73	Grey
135.	Asset Mapping	Understanding	63.64	Academic
136.	Goal Attainment Scaling (Gas)	Understanding	63.64	Academic

137.	Learning Log	Understanding	63.64	Grey
138.	Participatory Surveillance	Understanding	63.64	Academic
139.	Product Prototyping	Understanding	63.64	Academic
140.	Reflective Writing	Understanding	63.64	Academic
141.	The Five Whys Method	Understanding	63.64	Academic
142.	Direct Experience Storyboard	Understanding	63.64	Grey
143.	Emotional Journey	Understanding	63.64	Grey
144.	Experience Mapping /Experience Mapping / Customer Journey Mapping	Understanding	63.64	Grey
145.	Guided Conversation	Understanding	63.64	Academic
146.	Reflective Practice	Understanding	63.64	Academic
147.	Resource Flow	Understanding	63.64	Grey
148.	Retrospective Case Note Review	Understanding	63.64	Academic
149.	Summing Up Rounds	Understanding	63.64	Grey
150.	User Tests	Understanding	63.64	Grey
151.	Videovoice / Videovoice Diaries	Understanding	63.64	Academic
152.	Visual Narratives / Visual Storytelling	Understanding	63.64	Academic
153.	Writing A Letter To Yourself	Understanding	63.64	Grey
154.	4 Plus 1 Question	Understanding	54.55	Grey
155.	Break Up Letter / Love Letter	Understanding	54.55	Grey
156.	Card Sort	Understanding	54.55	Academic
157.	Empathy Map / Empathy Mapping	Understanding	54.55	Grey
158.	Feedback Map	Understanding	54.55	Grey
159.	Five-Fingered Consensus/ Fist To Five	Understanding	54.55	Grey
160.	Fuzzy Cognitive Mapping	Understanding	54.55	Academic
161.	GIS Mapping Techniques / GIS	Understanding	54.55	Academic
162.	Good Day/Bad Day	Understanding	54.55	Grey
163.	Group Delphi	Understanding	54.55	Grey
164.	Mind Mapping	Understanding	54.55	Academic
165.	Photo Voice / Photovoice / Photo Interview(S) / Photo Diary	Understanding	54.55	Academic
166.	Power Mapping	Understanding	54.55	Academic
167.	Social Mapping	Understanding	54.55	Academic
168.	Speed Boat	Understanding	54.55	Grey
169.	Structured Reflection	Understanding	54.55	Academic
170.	The AIR Feedback Model	Understanding	54.55	Grey
171.	Audio-Recordings	Understanding	54.55	Academic
172.	Concept Walkthrough	Understanding	54.55	Grey
173.	Deliberative Poll / Deliberative Polling	Understanding	54.55	Grey
174.	Narrative Scenarios	Understanding	54.55	Academic
175.	Narratives	Understanding	54.55	Academic
176.	Personal Take-Aways	Understanding	54.55	Grey
177.	Reflection Workshop(S)	Understanding	54.55	Academic
178.	Science Mapping	Understanding	54.55	Academic
179.	Service Safari	Understanding	54.55	Grey

180.	Show And Tell	Understanding	54.55	Grey
181.	The Circle Of Trust / Trust Circle	Understanding	54.55	Grey
182.	Thumbs Spectrum	Understanding	54.55	Grey
183.	Bodystorming	Stimulating	100	Grey
184.	I Love You Honey…	Stimulating	100	Grey
185.	Marimba!	Stimulating	100	Grey
186.	Shake All Hands	Stimulating	100	Grey
187.	Shake Down 5-4-3-2-1 / Shake Down	Stimulating	100	Grey
188.	Trigger Storming	Stimulating	100	Grey
189.	Figure Storming	Stimulating	100	Grey
190.	Go Bananas	Stimulating	100	Grey
191.	Hello Kitty	Stimulating	100	Grey
192.	Hot Air Balloon	Stimulating	100	Grey
193.	Love Your Neighbour?	Stimulating	100	Grey
194.	Portrait Drawing	Stimulating	100	Grey
195.	Rock Paper Scissor Tournament	Stimulating	100	Grey
196.	Stop And Go	Stimulating	100	Grey
197.	The Viking	Stimulating	100	Grey
198.	What Are You Doing?	Stimulating	100	Grey
199.	What's In The Bag?	Stimulating	100	Grey
200.	Who Are We Now?	Stimulating	100	Grey
201.	Who Is It?	Stimulating	100	Grey
202.	Keys Method	Stimulating	90.91	Grey
203.	Questorming	Stimulating	90.91	Grey
204.	The Reverse Perspective	Stimulating	90.91	Grey
205.	Count Up / Count To Ten	Stimulating	90.91	Grey
206.	Dance, Dance, Dance	Stimulating	90.91	Grey
207.	Draw Toast	Stimulating	90.91	Grey
208.	Find Your Pair	Stimulating	90.91	Grey
209.	Graphic Jam	Stimulating	90.91	Grey
210.	Green, Heart, I	Stimulating	90.91	Grey
211.	Ideastorming / Ideastorms	Stimulating	90.91	Grey
212.	Line-Up	Stimulating	90.91	Grey
213.	Pronoun Rounds	Stimulating	90.91	Grey
214.	Reverse Brainstorming / Reverse Thinking	Stimulating	90.91	Grey
215.	Start Your Day	Stimulating	90.91	Grey
216.	The Shouting Game	Stimulating	90.91	Grey
217.	"What If" Brainstorming / What If-Ing	Stimulating	81.82	Grey
218.	4 Quadrants	Stimulating	81.82	Grey
219.	Brainstorming	Stimulating	81.82	Academic
220.	Challenge Prizes	Stimulating	81.82	Grey
221.	Draw An Apple / Apple-Drawing Ideation	Stimulating	81.82	Grey
222.	Portrait Gallery	Stimulating	81.82	Grey
223.	Round Robin / Round Robin Brainstorming	Stimulating	81.82	Grey
224.	Sky The Limit Brainstorm	Stimulating	81.82	Grey
225.	Starbursting	Stimulating	81.82	Grey

226.	Trust Falls In Pairs	Stimulating	81.82	Grey
227.	Two Truths And A Lie	Stimulating	81.82	Grey
228.	Wizards, Pixies, Giants / Giants, Wizards, Elves	Stimulating	81.82	Grey
229.	Bed Sheet Ping-Pong	Stimulating	81.82	Grey
230.	Connect The Dots	Stimulating	81.82	Grey
231.	Human Machine	Stimulating	81.82	Grey
232.	Knot Game / Untangle Yourself	Stimulating	81.82	Grey
233.	Lower A Bamboo Cane To The Ground	Stimulating	81.82	Grey
234.	Pass A Loop Round The Circle	Stimulating	81.82	Grey
235.	Pass The Squeeze	Stimulating	81.82	Grey
236.	Rain Making	Stimulating	81.82	Grey
237.	Role Storming / Rolestorming	Stimulating	81.82	Grey
238.	Shrinking Islands	Stimulating	81.82	Grey
239.	Structured Brainstorm	Stimulating	81.82	Academic
240.	Sync Claps	Stimulating	81.82	Grey
241.	The 6 Thinking Hats / Feedback Hats	Stimulating	81.82	Grey
242.	Trading Cards	Stimulating	81.82	Grey
243.	Trust Ladder	Stimulating	81.82	Grey
244.	Walkabout	Stimulating	81.82	Grey
245.	3-12-3 Brainstorm	Stimulating	72.73	Grey
246.	Apple-Drawing Ideation	Stimulating	72.73	Grey
247.	Bisociation	Stimulating	72.73	Grey
248.	Check-In / Check-Out	Stimulating	72.73	Grey
249.	Cover Story	Stimulating	72.73	Grey
250.	Crazy 8's	Stimulating	72.73	Grey
251.	Forced Analogy	Stimulating	72.73	Grey
252.	Mingle	Stimulating	72.73	Grey
253.	Mirroring	Stimulating	72.73	Grey
254.	My Daily P.A.C.T	Stimulating	72.73	Grey
255.	Post-Up	Stimulating	72.73	Grey
256.	Quiet Brainstorming / Silent Brainstorming	Stimulating	72.73	Grey
257.	Super Hero	Stimulating	72.73	Grey
258.	Who Inspires Us?	Stimulating	72.73	Grey
259.	Write A Blurb	Stimulating	72.73	Grey
260.	Body Part Twister	Stimulating	72.73	Grey
261.	Image-ination	Stimulating	72.73	Grey
262.	Inspiration Walk	Stimulating	72.73	Grey
263.	Pair Introductions / Introduction In Pairs	Stimulating	72.73	Grey
264.	People Bingo	Stimulating	72.73	Grey
265.	Protobot	Stimulating	72.73	Grey
266.	Start Small	Stimulating	72.73	Grey
267.	Stinky Fish	Stimulating	72.73	Grey
268.	The Anti-Problem	Stimulating	72.73	Grey
269.	This N' That	Stimulating	72.73	Grey
270.	5-4-3-2-1 Grounding Technique	Stimulating	63.64	Grey
271.	6*8*5 Method	Stimulating	63.64	Grey

272.	Brain Writing / Brainwriting / Group Passing / Slip Writing	Stimulating	63.64	Grey
273.	Feedback: Appreciation Mingle	Stimulating	63.64	Grey
274.	Feedback: I Appreciate…	Stimulating	63.64	Grey
275.	Future Wheel	Stimulating	63.64	Grey
276.	I Like, I Wish, I Wonder / I Like, I Wish, What If	Stimulating	63.64	Grey
277.	Jam Session / Design Jam / Innovation Jam	Stimulating	63.64	Grey
278.	Lo-Tech Social Networking / Low-Tech Social Network	Stimulating	63.64	Grey
279.	Marshmallow Challenge	Stimulating	63.64	Grey
280.	Mood Board(S)	Stimulating	63.64	Grey
281.	NUF Test	Stimulating	63.64	Grey
282.	Opening Circle	Stimulating	63.64	Grey
283.	Prune The Future	Stimulating	63.64	Grey
284.	Question Balloons	Stimulating	63.64	Grey
285.	Roving Ideastorms	Stimulating	63.64	Grey
286.	Squiggle Birds	Stimulating	63.64	Grey
287.	Synesthesia	Stimulating	63.64	Grey
288.	Talking Stick(S) / Wooden Stick	Stimulating	63.64	Grey
289.	Air Time Mastermind	Stimulating	63.64	Grey
290.	Crazy 8	Stimulating	63.64	Grey
291.	Crocodile River	Stimulating	63.64	Grey
292.	Current Strongest Impression	Stimulating	63.64	Grey
293.	Dinner Challenge	Stimulating	63.64	Grey
294.	Go-Round(S)	Stimulating	63.64	Grey
295.	Mission Impossible	Stimulating	63.64	Grey
296.	Object Brainstorm	Stimulating	63.64	Grey
297.	Picture Yourself	Stimulating	63.64	Grey
298.	Prune The Product Tree	Stimulating	63.64	Grey
299.	Random Inputs	Stimulating	63.64	Grey
300.	Rollercoaster / Rollercoaster Check-In	Stimulating	63.64	Grey
301.	Science Fiction Prototyping (SFP)	Stimulating	63.64	Grey
302.	The Blind Side	Stimulating	63.64	Grey
303.	Whole Product Game	Stimulating	63.64	Grey
304.	A Journey Of Discovery	Stimulating	54.55	Grey
305.	Campfire Method	Stimulating	54.55	Grey
306.	Ethos, Logos, Pathos	Stimulating	54.55	Grey
307.	Feedback: Start, Stop, Continue / Start, Stop, Continue	Stimulating	54.55	Grey
308.	Hero Stories	Stimulating	54.55	Grey
309.	How-Now-Wow Matrix	Stimulating	54.55	Grey
310.	Kanban Pizza Game	Stimulating	54.55	Grey
311.	LEGO Serious Play / LEGO® Serious Play	Stimulating	54.55	Academic
312.	Lego Challenge	Stimulating	54.55	Grey
313.	Letter To Myself	Stimulating	54.55	Grey
314.	Quickly Sketch Noting / Sketchnoting	Stimulating	54.55	Grey

315.	Role-Playing Game / Role Play(S)	Stimulating	54.55	Academic
316.	Skill Share	Stimulating	54.55	Grey
317.	Talking Chips	Stimulating	54.55	Grey
318.	WhoDo	Stimulating	54.55	Grey
319.	Brainwriting 6-3-5	Stimulating	54.55	Grey
320.	Closing Circle	Stimulating	54.55	Grey
321.	Coach Yourself Out Of A Funk In 3 Steps	Stimulating	54.55	Grey
322.	Difficult Co-Creators	Stimulating	54.55	Grey
323.	Extreme Characters	Stimulating	54.55	Grey
324.	Future Backcasting	Stimulating	54.55	Grey
325.	Habit Reflection: Create A Habit That Sticks	Stimulating	54.55	Grey
326.	Here, There, Everywhere	Stimulating	54.55	Grey
327.	Innovation Tournament	Stimulating	54.55	Academic
328.	Paint A Picture	Stimulating	54.55	Grey
329.	Story Puzzle	Stimulating	54.55	Grey
330.	Team Brainstorming	Stimulating	54.55	Academic
331.	Tomorrow's Narratives / Tomorrow's Headline / Advertising Posters / Service Evidencing / Future Newspaper / Newspaper Headlines Of The Future	Stimulating	54.55	Grey
332.	Unintended Consequences / Dormant Opportunities	Stimulating	54.55	Grey
333.	Flower Of Thoughts	Collaborating	100	Grey
334.	Fish Bowl / Fishbowl	Collaborating	100	Grey
335.	Collective Reflection	Collaborating	90.91	Academic
336.	Collective Vision Building	Collaborating	90.91	Academic
337.	Idea & Concept Development	Collaborating	90.91	Grey
338.	Open Space Technology	Collaborating	90.91	Grey
339.	Walt Disney Method	Collaborating	90.91	Grey
340.	World Café	Collaborating	90.91	Academic
341.	Collective Narratives	Collaborating	90.91	Academic
342.	90-Minute Prototypes	Collaborating	81.82	Grey
343.	Align On Your Impact Goals	Collaborating	81.82	Grey
344.	Idea Remix	Collaborating	81.82	Grey
345.	Sketching Group / C-Sketch / Collaborative Sketching / 5-1-3 G	Collaborating	81.82	Grey
346.	Alignment & Autonomy	Collaborating	72.73	Grey
347.	Carousel / Carousel Technique	Collaborating	72.73	Grey
348.	Design The Box	Collaborating	72.73	Grey
349.	Dilemma Café	Collaborating	72.73	Grey
350.	Group Model Building / Group-Model Building / Group Model-Building / Building A Model	Collaborating	72.73	Academic
351.	Participatory System Dynamics (SD)	Collaborating	72.73	Academic
352.	People Map / People Mapping	Collaborating	72.73	Grey

353.	Public Stack Puzzle	Collaborating	72.73	Grey
354.	1,2,4, All!	Collaborating	63.64	Grey
355.	5 Bold Steps	Collaborating	63.64	Grey
356.	Action Group(S)	Collaborating	63.64	Academic
357.	Collaborative Pilot Schedule	Collaborating	63.64	Grey
358.	Dialogue Cafés	Collaborating	63.64	Academic
359.	Dialogue Of Wisdom (Wisdom Dialogues)	Collaborating	63.64	Academic
360.	Do, Redo & Undo	Collaborating	63.64	Grey
361.	Geographical Mapping / GIS	Collaborating	63.64	Grey
362.	Group Level Assessment	Collaborating	63.64	Academic
363.	Grow An Idea	Collaborating	63.64	Grey
364.	Innovation Portfolio Exercise	Collaborating	63.64	Grey
365.	Lights, Camera, Action!	Collaborating	63.64	Grey
366.	Nagging	Collaborating	63.64	Grey
367.	Neo-Socratic Dialogue	Collaborating	63.64	Grey
368.	Vision Factory	Collaborating	63.64	Grey
369.	Collaboration Analysis/ Collaborative Data Analysis	Collaborating	63.64	Academic
370.	Draw The Problem	Collaborating	63.64	Grey
371.	Future Mapping / Future Map	Collaborating	63.64	Grey
372.	Make A World	Collaborating	63.64	Grey
373.	Mini-Campaign Challenge	Collaborating	63.64	Grey
374.	Participatory Photography (PP)	Collaborating	63.64	Academic
375.	Video Card Family Game	Collaborating	63.64	Grey
376.	Atomize	Collaborating	54.55	Grey
377.	Cogenerative Dialogues (Cogens)	Collaborating	54.55	Academic
378.	Four Square Story	Collaborating	54.55	Grey
379.	Life Café	Collaborating	54.55	Academic
380.	Participatory Theatre	Collaborating	54.55	Academic
381.	Post-It Note Race / Sticky Note Race	Collaborating	54.55	Grey
382.	Quaker Conversation / Quaker Dialogue	Collaborating	54.55	Grey
383.	The Blue Sky Vision Exercise / Blue Skye Envisioning	Collaborating	54.55	Grey
384.	Argument Map / Argument Mapping	Collaborating	54.55	Grey
385.	Bundle Ideas	Collaborating	54.55	Grey
386.	Challenge Cards	Collaborating	54.55	Grey
387.	Collaborative Mapping (Aka Citizen Mapping)	Collaborating	54.55	Academic
388.	Conflict Responses	Collaborating	54.55	Grey
389.	Customer, Employee, Shareholder	Collaborating	54.55	Grey
390.	Design Critique	Collaborating	54.55	Grey
391.	Engineering Your Team OS	Collaborating	54.55	Grey
392.	Friend Or Foe?	Collaborating	54.55	Grey
393.	Future Trends	Collaborating	54.55	Grey
394.	Mash-Up Innovation / Mash-Ups	Collaborating	54.55	Grey
395.	Scenario Mapping	Collaborating	54.55	Academic

396.	Dotmocracy / Dot Voting / Coloured Dot Voting / Prioritisation Dots	Decision-Making	100	Grey
397.	Impact/Effort Matrix / Impact & Effort Matrix	Decision-Making	100	Grey
398.	Near And Dear	Decision-Making	81.82	Grey
399.	Yonmenkaigi System Method	Decision-Making	81.82	Academic
400.	Pair-Wise Ranking	Decision-Making	81.82	Academic
401.	20/20 Vision	Decision-Making	72.73	Grey
402.	3 Action Steps	Decision-Making	72.73	Grey
403.	Non-Structural Fuzzy Decision Support System	Decision-Making	72.73	Academic
404.	Simple Multi-Attribute Rating Technique (SMART)	Decision-Making	72.73	Academic
405.	Sorting Important To/For	Decision-Making	72.73	Grey
406.	Electronic Voting	Decision-Making	72.73	Academic
407.	Direct Ranking / Direct Matrix Ranking	Decision-Making	63.64	Academic
408.	Forced Ranking	Decision-Making	63.64	Grey
409.	Urgent/Important Grid (Aka Eisenhower Matrix, Or Eisenhower Decision Matrix)	Decision-Making	63.64	Grey
410.	Diamond Ranking / Diamond Ranking Strategy	Decision-Making	63.64	Grey
411.	Deliberative Dialogue	Decision-Making	54.55	Academic
412.	Interdisciplinary Work Groups	Decision-Making	54.55	Grey
413.	Narrow The Set	Decision-Making	54.55	Grey
414.	Soft Systems Methodology	Decision-Making	54.55	Academic
415.	Bang-For-The-Buck	Decision-Making	54.55	Grey
416.	Pros & Cons / Pros Cons List	Decision-Making	54.55	Grey
417.	Content Analysis / Qualitative Content Analysis	Analysis	100	Academic
418.	Foucauldian Discourse Analysis	Analysis	100	Academic
419.	Structural Equation Modeling (SEM)	Analysis	100	Academic
420.	Thematic Analysis (TA)	Analysis	100	Academic
421.	Discourse Analysis	Analysis	100	Academic
422.	Framework Analysis	Analysis	100	Academic

423.	Fuzzy Set Qualitative Comparative Analysis (FS/QCA)	Analysis	100	Academic
424.	Quantitative Bibliometric Analysis	Analysis	100	Academic
425.	Critical Appraisal	Analysis	90.91	Academic
426.	Comparison Analysis	Analysis	90.91	Academic
427.	Opinion Mining / Sentiment Analysis	Analysis	81.82	Grey
428.	Two-Step Clustering Method	Analysis	81.82	Academic
429.	Analytic Hierarchy Process	Analysis	72.73	Academic
430.	Cost Benefit Analysis / Cost-Benefit Analysis	Analysis	72.73	Academic
431.	Quantitative Forecasting	Analysis	72.73	Academic
432.	Legal Analysis	Analysis	63.64	Academic
433.	Systematic Mapping	Analysis	63.64	Academic
434.	Analytic Network Process	Analysis	54.55	Academic
435.	Benchmarking	Analysis	54.55	Academic
436.	Test-Retest Method / Test-Retest Reliability	Analysis	54.55	Academic

Outlier Cards (unsorted Methods):

These cards had too low of an agreement rate to be placed in a section of the framework. Therefore, these methods will not be added to the Co-Creation Rainbow framework at this stage.

**Table S2. Outlier Cards (unsorted Methods)**				
**Number**	**Method name**	**Potential Section(s)**	**Avg. Agreement rate (%)**	**Sourced from Grey or Academic Lit.**
1.	Scenario Workshop(S)	Collaborating, Decision-Making or Stimulating	33.33	Grey
2.	History Map / History Mapping	Collaborating, Stimulating, or Understanding	33.33	Grey
3.	A/B Testing	Analysis, Understanding, or Collaborating	33.33	Grey
4.	Deliberative Online Forum	Collaborating, Decision-Making or Understanding	33.33	Grey
5.	Democs (Deliberative Meetings Organised By Citizens)	Decision-Making, Collaborating, Stimulating	33.33	Grey
6.	Is - Is Not - Does - Does Not	Stimulating, Collaborating, or Understanding	33.33	Grey
7.	Matchstick Discussion	Collaborating, Stimulating or Understanding	33.33	Grey
8.	Onboarding Kit	Informing, Collaborating, or Stimulating	33.33	Grey
9.	Paired Listening	Stimulating, Collaborating or Understanding	33.33	Grey
10.	Science Theatre	Stimulating, Collaborating, or Informing	33.33	Grey
11.	$100 Test	Decision-Making, Stimulating, or Collaborating	33.33	Grey

12.	Button Method	Collaborating, Stimulating, or Understanding	33.33	Grey
13.	Caravan Method	Stimulating, Collaborating or Understanding	33.33	Grey
14.	Circle Of Influence	Collaborating, Decision-Making or Understanding	33.33	Grey
15.	Collaborative Brainstorming / Crowdstorming	Collaborating, Stimulating, or Understanding	33.33	Grey
16.	Elevator Pitch	Stimulating, Informing or Understanding	33.33	Grey
17.	Explore Your Values	Stimulating, Understanding, or Decision-Making	33.33	Grey
18.	Exploring Client Centricity	Collaborating, Understanding, or Decision-Making	33.33	Grey
19.	Find Themes	Analysis, Understanding or Collaborating	33.33	Grey
20.	Flip It	Collaborating, Stimulating, or Informing	33.33	Grey
21.	Hero's Journey Agenda	Informing, Stimulating, or Understanding	33.33	Grey
22.	Knowledge Hunt	Collaborating, Stimulating and Understanding	33.33	Grey
23.	Lightning Decision Jam	Collaborating, Decision-Making, Understanding	33.33	Grey
24.	One Word Before Leaving	Stimulating, Understanding, and Decision-Making	33.33	Grey
25.	Paired Roleplay	Stimulating, Collaborating, or Decision-Making	33.33	Grey
26.	Personal Presentations	Stimulating, Informing, Understanding	33.33	Grey
27.	Plus/Delta	Understanding, Collaborating, or Stimulating	33.33	Grey
28.	Post The Path	Understanding, Collaborating, or Stimulating	33.33	Grey
29.	Rapid Research	Collaborating, Stimulating, or Understanding	33.33	Grey
30.	Relationship Circle	Collaborating, Analysis, or Understanding	33.33	Grey
31.	Reverse Role Play	Collaborating, Stimulating or Decision-Making	33.33	Grey
32.	Stakeholder Working Groups	Understanding, Decision-Making, or Collaborating	33.33	Grey
33.	Step Ladder Brainstorming / Stepladder Technique	Decision-making, Stimulating, or Collaborating	33.33	Grey
34.	Take A Stand	Collaborating, Stimulating, or Understanding	33.33	Grey

35.	The Sketch Game	Stimulating, Collaborating, or Understanding	33.33	Grey
36.	Understanding Chain / Understanding Chain Game	Collaborating, Stimulating, or Understanding	33.33	Grey
37.	Welcome To My World	Understanding, Collaborating, or Stimulating	33.33	Grey
38.	Concept Mapping / Conceptual Mapping	Understanding, Analysis, or Collaborating	33.33	Academic
39.	Critical Dialogue	Stimulating, Collaborating or Understanding	33.33	Academic
40.	Two-Way Dialogue / Two-Way Communication	Collaborating, Stimulating, or Understanding	33.33	Academic
41.	Causal Loop Diagrams	Understanding, Analysis or Collaborating	33.33	Academic
42.	Likert Scale	Understanding, Decision-Making or Analysis	33.33	Academic
43.	Reflection Meetings	Stimulating, Understanding or Collaborating	33.33	Academic
44.	Memos	Analysis, Understanding or Stimulating	33.33	Academic
45.	Scenario Analysis	Analysis, Understanding or Collaborating	33.33	Academic
46.	Stakeholder Mapping	Understanding, Analysis or Collaborating	33.33	Academic
47.	Facilitated Dialogue	Collaborating, Stimulating or Understanding	33.33	Academic
48.	Forum Theatre	Collaborating, Stimulating, or Informing	33.33	Academic
49.	Participatory Social Network Mapping	Collaborating, Understanding, or Analysis	33.33	Academic
50.	Wealth Ranking	Decision-Making, Understanding, Collaborating	33.33	Academic
51.	Participatory Art-Based Inquiry	Stimulating, Collaborating, or Understanding	33.33	Academic
52.	Reflective Group Sessions	Understanding, Collaborating, or Stimulating	33.33	Academic
53.	Transformative Dialogues (TD)	Collaborating, Understanding or Stimulating	33.33	Academic
54.	Note To Self	Stimulating, Understanding, or Analysis	30.3	Grey
55.	Hand Signals	Stimulating, Collaborating, or Informing	30.3	Grey
56.	Red: Green Cards	Understanding, Stimulating, or Collaborating	30.3	Grey
57.	Circles Of Connection	Collaborating, Stimulating, Understanding or Decision-Making	25	Grey

58.	Deliberative Poll / Deliberative Polling	Understanding, Decision-making, Informing or Stimulating	25	Grey
59.	Idea Card(S)	Collaborating, Stimulating, Decision-Making, or Understanding	25	Grey
60.	Idea Dashboard	Stimulating, Informing, Collaborating or Understanding	25	Grey
61.	Parking Lot / Parking Space	Collaborating, Stimulating, Decision-Making, or Understanding	25	Grey
62.	Six Thinking Hats	Stimulating, Collaborating, Understanding, or Analysis	25	Grey
63.	Valorisation Panels / User Committee	Decision-Making, Collaborating, Understanding, or Stimulating	25	Grey
64.	Commons Mapping	Understanding, Collaborating, Decision-making, or Stimulating	25	Grey
65.	Context Mapping	Collaborating, Understanding, Stimulating, or Informing	25	Grey
66.	Controlled Dialogue	Stimulating, Collaborating, Informing or Understanding	25	Grey
67.	Mapping Organizational Culture	Collaborating, Understanding, Stimulating, or Informing	25	Grey
68.	Spectrum Lines	Decision-Making, Collaborating, Stimulating, or Understanding	25	Grey
69.	Stakeholder(S) Trust Map	Understanding, Collaborating, Analysis or Stimulating	25	Grey
70.	Street Vote	Understanding, Decision-Making, Stimulating, or Collaborating	25	Grey
71.	Value Map / Value Mapping	Understanding, Collaborating, Analysis or Decision-Making	25	Grey
72.	Walk Shop	Understanding, Collaborating, Stimulating, or Informing	25	Grey
73.	Action Plan Workshop: The Arrow / The Arrow	Collaborating, Decision-Making, Stimulating, or Informing	25	Grey
74.	Analogous Inspiration	Decision-Making, Collaborating, Analysis or Understanding	25	Grey
75.	Appreciation Train	Stimulating, Collaborating, Analysis or Understanding	25	Grey

76.	Back-Turned Feedback	Understanding, Collaborating, Stimulating, or Analysis	25	Grey
77.	Build & Run Prototypes	Collaborating, Understanding, Informing, or Stimulating	25	Grey
78.	Circles And Soup	Decision-Making, Collaborating, Understanding, or Stimulating	25	Grey
79.	Demands Continuums	Collaborating, Understanding, Analysis, or Stimulating	25	Grey
80.	Diffusion Curve Reflection	Stimulating, Understanding, Collaborating or Analysis	25	Grey
81.	Draw It	Stimulating, Understanding, Collaborating, or Informing	25	Grey
82.	Dynamic Personas	Collaborating, Understanding, Analysis or Stimulating	25	Grey
83.	Ecology Mapping (*Same As Ecosystem Map)	Understanding, Collaborating, Stimulating, or Analysis	25	Grey
84.	Function Vision Mapping / Function Mapping	Collaborating, Analysis, Understanding, or Decision-Making	25	Grey
85.	Graphic Gameplan	Collaborating, Decision-Making, Understanding, or Stimulating	25	Grey
86.	Gut Check	Stimulating, Collaborating, Understanding, or Decision-Making	25	Grey
87.	Heart, Hand, Mind	Stimulating, Collaborating, Understanding, or Decision-Making	25	Grey
88.	Heuristic Ideation Technique	Collaborating, Stimulating, Analysis, or Informing	25	Grey
89.	Live Prototyping	Analysis, Collaborating, Understanding, Decision-Making	25	Grey
90.	Memory Wall	Stimulating, Collaborating, Analysis or Understanding	25	Grey
91.	Methodkit For Team Development	Collaborating, Understanding, Stimulating, or Analysis	25	Grey
92.	Myers-Briggs Team Reflection	Collaborating, Stimulating, Understanding, or Analysis	25	Grey
93.	Pattern Quest	Collaborating, Understanding, Analysis or Stimulating	25	Grey

94.	Pessimist Vs. Optimist	Collaborating, Stimulating, Understanding, or Informing	25	Grey
95.	Pre-Mortem / Premortem	Collaborating, Stimulating, Understanding, or Analysis	25	Grey
96.	Product Pinocchio	Collaborating, Decision-Making, Stimulating or Understanding	25	Grey
97.	Project Wrap-Up	Understanding, Collaborating, Stimulating, or Decision-Making	25	Grey
98.	Question Walk / Walking Questions	Stimulating, Collaborating, Informing or Understanding	25	Grey
99.	Rings Of Connection	Stimulating, Collaborating, Analysis or Understanding	25	Grey
100.	Scenario Slider	Collaborating, Stimulating, Analysis, or Understanding	25	Grey
101.	Selection Idea Matrix/Idea Selection Table	Collaborating, Decision-Making, Analysis or Stimulating	25	Grey
102.	Share Inspiring Stories	Understanding, Stimulating, Collaborating, or Informing	25	Grey
103.	SOAR Analysis	Analysis, Collaborating, Stimulating or Understanding	25	Grey
104.	Staple Yourself To Something	Stimulating, Collaborating, Informing or Understanding	25	Grey
105.	Synthesis Wall	Collaborating, Understanding, Decision-Making or Informing	25	Grey
106.	System Map / System Mapping	Collaborating, Understanding, Analysis or Stimulating	25	Grey
107.	System Scenario	Collaborating, Understanding, Analysis, or Stimulating	25	Grey
108.	Team Purpose & Culture	Collaborating, Understanding, Stimulating, or Informing	25	Grey
109.	Team Retrospective / Retros	Understanding, Stimulating, Collaborating, or Informing	25	Grey
110.	Teams Self-Assessment	Understanding, Collaborating, Stimulating, or Analysis	25	Grey
111.	The Doughnut	Stimulating, Understanding, Collaborating, or Informing	25	Grey
112.	Timelapse	Understanding, Stimulating, Collaborating, or Analysis	25	Grey

113.	Transition Journey	Understanding, Collaborating, Informing, or Stimulating	25	Grey
114.	User Day-Parting	Stimulating, Collaborating, Analysis or Understanding	25	Grey
115.	Ways To Grow Framework	Collaborating, Stimulating, Understanding, or Analysis	25	Grey
116.	Participatory GIS / PGIS / Public Participation GIS	Collaborating, Understanding, Stimulating, or Decision-Making	25	Academic
117.	Nominal Group Technique	Decision-Making, Stimulating, Collaborating, or Analysis	25	Academic
118.	Problem Trees / Problem Analysis / Situational Analysis	Understanding, Collaborating, Analysis, or Stimulating	25	Academic
119.	Forecasting	Decision-Making, Understanding, Analysis, or Stimulating	25	Academic
120.	Stakeholder Dialogue(S)	Collaborating, Stimulating, Understanding, or Informing	25	Academic
121.	Think Aloud / Think-Aloud / Thinking Aloud	Stimulating, Understanding, Informing, or Collaborating	25	Academic
122.	Reflexive Dialogue / Reflective Dialogue(S)	Collaborating, Stimulating, Understanding or Analysis	25	Academic
123.	Participatory Reflection (And Action)	Collaborating, Understanding, Stimulating, or Decision-Making	25	Academic
124.	Constructive Dialogue	Collaborating, Stimulating, Understanding, or Analysis	25	Academic
125.	Participatory Systems Mapping (PSM)	Collaborating, Understanding, Analysis, or Decision-Making	25	Academic
126.	Participatory Theme Elicitation (PTE)	Collaborating, Understanding, Analysis or Stimulating	25	Academic
127.	Visual Mapping	Understanding, Analysis, Collaborating, or Stimulating	25	Academic
128.	Scenario Narratives / Exploratory Scenario Narratives	Decision-Making, Understanding, Stimulating, or Collaborating	25	Academic
129.	Critical Self-Reflection (Reflective Essay)	Stimulating, Understanding, Analysis or Collaborating	25	Academic
130.	Design Meeting	Decision-Making, Collaborating, Analysis or Informing	25	Academic

131.	Horizontal Dialogues	Understanding, Stimulating, Collaborating, or Informing	25	Academic
132.	Most Significant Change Method / Most Significant Change Technique (MSC)	Collaborating, Understanding, Analysis or Decision-Making	25	Academic
133.	Actor-Centred Mapping / Actor Mapping	Understanding, Analysis, Collaborating or Informing	25	Academic
134.	ARDI (Actors Resources Dynamics And Interactions) Approach	Understanding, Analysis, Collaborating or Informing	25	Academic
135.	Arts-Infused Dialogue / Art Dialogue	Understanding, Collaborating, Stimulating, or Informing	25	Academic
136.	Optimized Honeycomb Model For User Experience (UX)	Understanding, Analysis, Informing, Stimulating	22.73	Academic
137.	Back Of The Napkin	Informing, Stimulating, Analysis or Collaborating	22.73	Grey
138.	Signal Cards	Understanding, Stimulating, Collaborating, or Informing	22.73	Grey
139.	7ps Framework	Stimulating, Understanding, Collaborating, or Informing	22.73	Grey
140.	Balloon And Kite Mapping	Understanding, Stimulating, Analysis, or Collaborating	22.73	Grey
141.	Extremes And Mainstreams	Understanding, Stimulating, Analysis or Collaborating	22.73	Grey
142.	Iteration Dice	Decision-Making, Stimulating, Collaborating, or Informing	22.73	Grey
143.	Rapid Prototyping	Understanding, Collaborating, Analysis or Stimulating	22.73	Academic
144.	Listening Levels	Understanding, Collaborating, Stimulating, Analysis or Informing	20	Grey
145.	Who/What/When Matrix	Decision-Making, Collaborating, Informing, Stimulating or Analysis	20	Grey
146.	Affinity Diagram / Affinity Diagramming / Affinity Map / Affinity Mapping	Collaborating, Analysis, Understanding, Informing or Stimulating	20	Grey

147.	Ambition Ranking	Decision-Making, Understanding, Stimulating, Analysis or Collaborating	20	Grey
148.	Photo Safari	Collaborating, Understanding, Analysis, Decision-Making or Stimulating	20	Grey
149.	Stakeholder Journey	Understanding, Stimulating, Collaborating, Analysis, or Informing	20	Grey
150.	2 Minutes Madness	Stimulating, Analysis, Collaborating, Informing or Understanding	20	Grey
151.	3d-Prop	Collaborating, stimulating, Analysis, Informing, or Understanding	20	Grey
152.	Actions For Retrospectives	Collaborating, Decision-Making, Stimulating, Informing, or Understanding	20	Grey
153.	Altitude	Stimulating, Collaborating, Analysis, Decision-Making or Informing	20	Grey
154.	Blink Testing	Understanding, Stimulating, Analysis, Collaborating, or informing	20	Grey
155.	Ecosystems Loops	Understanding, Collaborating, Analysis, Informing, or Stimulating	20	Grey
156.	Frame Your Design Challenge	Collaborating, Analysis, Stimulating, Understanding, or Informing	20	Grey
157.	Long Range Forecasts	Collaborating, Informing, Stimulating, Understanding or Decision-Making	20	Grey
158.	Paper Prototyping	Collaborating, Understanding, Analysis, Informing or Stimulating	20	Grey
159.	Personal Swot Assessment	Understanding, Analysis, Stimulating, Collaborating, or Informing	20	Grey
160.	Project Mid-Way Evaluation	Collaborating, Understanding, Analysis, Decision-Making or Stimulating	20	Grey
161.	Project Point Of Departure	Collaborating, Decision-Making, Understanding, Informing or Stimulating	20	Grey
162.	Quantify Yourself	Stimulating, Understanding, Analysis, Collaborating, or Informing	20	Grey

163.	Remember The Future	Understanding, Collaborating, Decision-Making, Analysis, or Stimulating	20	Grey
164.	Rough Prototyping	Collaborating, Analysis, Stimulating, Understanding, or Informing	20	Grey
165.	Service Roadmap	Collaborating, Informing, Analysis, Decision-Making or Stimulating	20	Grey
166.	Spectrum Mapping	Understanding, Analysis, Collaborating, Stimulating, or Decision-Making	20	Grey
167.	Telling Our Stories	Collaborating, Understanding, Stimulating, Analysis, or Informing	20	Grey
168.	Digital Stories / Digital Storytelling	Stimulating, Analysis, Collaborating, Understanding, or Informing	20	Academic
169.	Convenience Sampling	Analysis, Understanding, Collaborating, Informing or Stimulating	20	Academic
170.	Purposeful Sampling / Judgmental Sampling	Analysis, Understanding, Collaborating, Informing or Stimulating	20	Academic
171.	Random Sampling	Analysis, Understanding, Collaborating, Informing or Stimulating	20	Academic
172.	Rapid Participatory Appraisal (RPA)	Understanding, Collaborating, Analysis, Decision-Making or Stimulating	20	Academic
173.	Maximum Variation Sampling	Analysis, Understanding, Collaborating, Informing, or Stimulating	20	Academic
174.	Impact Mapping	Understanding, Collaborating, Decision-Making, Analysis or Informing	20	Academic
175.	Opportunistic Sampling	Analysis, Understanding, Collaborating, Informing or Stimulating	20	Academic
176.	Pair-Wise Comparison	Analysis, Decision-Making, Collaborating, Stimulating, or Understanding	20	Academic
177.	Solution Trees / Opportunity Solution Tree (OST)	Analysis, Understanding, Collaborating, Informing or Stimulating	20	Academic
178.	Temperature Check	Stimulating, Understanding, Analysis, Collaborating or Informing	18.18	Grey

179.	Seasonal Calendar	Understanding, Informing, Analysis, Collaborating, or Stimulating	18.18	Academic
180.	Values Tree	Stimulating, Collaborating, Informing, Analysis, Decision-Making or Understanding	16.67	Grey
181.	Assumption Mapper / Assumption Mapping	Collaborating, Decision-Making, Understanding, Analysis, Informing or Stimulating	16.67	Grey
182.	Road Mapping	Decision-Making, Analysis, Stimulating, Collaborating, Informing or Understanding	16.67	Grey
183.	Persona Method	Understanding, Decision-Making, Stimulating, Analysis, Collaborating or Informing	16.67	Academic

Post-Sorting Reflection Questions

When the card sorting participant completed the closed card sorting task, they were asked a few questions to gauge their immediate reflections. The following are the responses from the card-sorting participants.

Q1: Considering the options available, please add any additional categories you think were missing?

- "I was comfortable with the categories added. I would perhaps consider adding sub-groups to themes. Could be *that* stimulating is "stimulating exchange/idea creation" and separately something like "stimulating the process forward" (I added there some methods that helped recap and co-creators be on the same page, e.g. identify next steps, etc). Also, for the Understanding may be worth adding, as you mentioned, a distinction between ones that can be "active" and ones that can't be "actively involving co-creators."
- "maybe something about specific 'tools' for lack of a better word that would enable to group recordings, cards, visual tools more effectively?"
- "One consideration I had is to split stimulation into generation and energizing. "
- "I think I would consider a separate category for methods that can contribute to the co-creation process itself, for example, methods used for co-creators to get to know each other or building trust."
- "Maybe a category for cards that we consider not to be co-creation methods? "
- "I suggest expanding on the analysis category, maybe dividing it into collaborative analysis, theory/model/framework, and data analysis method."
- "no additional category is needed."

Q2: Please provide any reflections on the activity you did today, or your experience sorting the methods:

- "I appreciated the categories. I don't feel they need changing - just perhaps some further sub-themes or clarification. "

- "It was quite challenging to explore and understand the different categories, but once the cards were sorted (approximately halfway through) it becomes much clearer the division among the different categories."
- "it took me 4h. I became confused halfway through as the various items to be categorised are sometimes similar (for example why the distinction in so many different types of interviews?) or sometimes difficult to apportion to a scope when they are tools, like note to self or audio recording. "
- "It was faster than I thought, and actually very interesting. I also realized that I actually used way more methods in practice than I thought (I just didn't identify them as such)."
- "It was nice to see that most methods fit within the sections of the Co-Creation Rainbow framework. I think that if we had the opportunity to use a model/taxonomy like this when we were conducting our co-creation session it would have had a big impact on our preparation."
- "Super helpful to have the descriptions of the categories in-built to quickly refer to. Extremely helpful having the Word document methods and the method list in Maze in the same order because it was easier to match up and find methods to sort. "
- "This was difficult because there are nuances in levels of power and equality in each category, which can determine whether something is collaborative or not. "
- "I like the [Co-Creation Rainbow] framework. It looks super! But while sorting the methods, I found that there are some overlapping between collaborating and understanding, (like World Cafe...), and between informing and understanding (like product prototyping...)... It makes sense for the overlapping, I think. I think it is worth noting."
- “I highlighted some for myself to discuss whether these belong in methods."
- It took me over five hours – I wasn’t familiar with a lot of the methods so had to use the Word document, which was 34000 words so took a while to get through and Google.
- I’m not sure different types of sampling are ‘methods’ so I’d maybe remove them? Sampling would be done before any co-creation started."
- "I did not sort a few methods that I think should be removed from this set. There were some duplicates. Some mapping methods were hard to place between stimulating vs. collaborating. "

The Final Co-Creation Rainbow Framework

The Co-Creation Rainbow framework contains a total of 430 methods that were sorted into the 5 sections of the framework, and the ‘analysis’ category.

Informing = 8 methods

1. Audiovisual Patient Information
2. IDOARRT Meeting Design
3. Operation Manual(S)
4. Pecha Kucha / Ignite
5. Pie Chart Agenda
6. RACI Matrix
7. Train The Trainer / Train-The-Trainer
8. Video Prototyping

Understanding = 174 methods

1. 4 Plus 1 Question
2. Ad-Hoc User Acceptance Questionnaire
3. Asset Mapping
4. Audio-Recordings
5. Behavioural Observation
6. Bespoke Questionnaire
7. Break Up Letter / Love Letter
8. Capability Opportunity Motivation And Behaviour (COM-B) Self-Evaluation Questionnaire
9. Card Sort
10. Case Study Method
11. Cherokee Self-Reliance Questionnaire
12. Close Ended Questionnaire
13. Cognitive Mapping
14. Community Consultation
15. Community-Based Survey / Community Based Survey
16. Concept Walkthrough
17. Contextual Observation
18. Contingent Valuation
19. Convergent Interviews / Convergent Interviewing
20. Conversation Capture
21. Critical Incident Technique (Cit)
22. Cross-Sectional Observations
23. Cross-Sectional Survey
24. Daily Diaries / Daily Diary Method
25. Day-In-The-Life Mapping
26. Deliberative Poll / Deliberative Polling
27. Direct Experience Storyboard
28. Direct Observation(S)
29. Elicitation Interview
30. Emotional Journey
31. Emotional Touchpoints
32. Empathy Map / Empathy Mapping
33. Environmental Survey
34. Ethnographic Interviews
35. Ethnographic Observation
36. Evaluation Questionnaire
37. Evidence-Based Practice Questionnaire
38. Exit Interview(S)
39. Experience Mapping /Experience Mapping / Customer Journey Mapping
40. Experiential Observation
41. Expert Consultation
42. Expert Interviews
43. Explanatory Narratives
44. Exploratory Interviews
45. Face-To-Face Interviews / Face-To-Face Dialogue / In-Person Interviews
46. Feedback Map

47. Feedback Questionnaire
48. Field Notes / Ethnographic Field Notes / Observations Field Notes / Qualitative Field Notes / Personal Field Notes
49. Field Observation(S)
50. Five-Fingered Consensus/ Fist To Five
51. Fuzzy Cognitive Mapping (FCM)
52. Geo-Questionnaire
53. GIS Mapping Techniques / GIS
54. Goal Attainment Scaling (GAS)
55. Good Day/Bad Day
56. Group Delphi
57. Guided Conversation
58. Health Literacy Questionnaire
59. Health Questionnaire
60. Health Survey
61. In-Depth Interviews
62. Individual Interviews
63. Insights And Learnings
64. Interactive Interviewing
65. Intercept Interviews
66. Interviewer-Administered Questionnaire
67. Key Informant Interviews
68. Learning Log
69. Mental Mapping
70. Mind Mapping
71. Motivational Interviewing
72. Narrative Ethnography
73. Narrative Interview
74. Narrative Interviews
75. Narrative Scenarios
76. Narratives
77. Naturalistic Observations
78. Neighborhood Mapping
79. Non-Participant Observation
80. Observational Study
81. One-To-One Interviews
82. Online Questionnaire / Internet Questionnaire / Web-Based Questionnaire
83. Open-Ended Interview(S)
84. Open-Ended Questionnaire / Open Ended Questionnaire
85. Pain-Gain Map
86. Participant Observation
87. Participatory Observation(S)
88. Participatory Surveillance
89. Participatory Survey
90. Pathographies
91. Patient Diaries
92. Patient Narratives / Patient Experience Narratives
93. Patient Satisfaction Questionnaire

94. Peers Observing Peers
95. People Shadowing
96. Personal Diaries / Customer Diaries
97. Personal Narratives
98. Personal Take-Aways
99. Photo Voice / Photovoice / Photo Interview(S) / Photo Diary
100. Photojournal
101. Picker Patient Experience Questionnaire
102. Pictogram Interview
103. Postintervention Questionnaires
104. Power Mapping
105. Practical Observations
106. Practice Environment Scale Of The Nursing Work Index
107. Product Prototyping
108. Psychiatric Inpatient Experience Questionnaire (PIPEQ)
109. Psychiatric Out-Patient Experiences Questionnaire (POPEQ)
110. Q-Methodology
111. Qualitative Interviews
112. Qualitative Survey / Qualitative Questionnaire
113. Quality Involvement Questionnaire
114. Quality Of Life Questionnaire (QOLQ)
115. Quantitative Interviews / Standardized Interviews
116. Quantitative Survey
117. Questionnaire Survey / Questionnaire Surveys
118. Reference Project / Case Studies
119. Reflection Notes
120. Reflection Workshop(S)
121. Reflective Diaries
122. Reflective Field Notes / Reflexive Field Notes
123. Reflective Narratives
124. Reflective Practice
125. Reflective Writing
126. Repertory Grid Technique
127. Resource Flow
128. Retrospective Case Note Review
129. Satisfaction Questionnaire
130. Scenario-Based Interviews / Situational Interviews
131. Science Mapping
132. Second-Order Observation
133. Self-Administered Questionnaire
134. Self-Assessment Method
135. Self-Assessment Questionnaire
136. Self-Reporting Questionnaire (SRQ)
137. Semi-Structured Interviews / Semistructured Interviews / Semi-Structured Dialogues
138. Semistructured Questionnaire / Semi-Structured Questionnaire
139. Service Safari
140. Show And Tell
141. Show Me Your Values

142. Social Mapping
143. Speed Boat
144. Stakeholder Consultation
145. Stakeholder Interviews
146. Stakeholder Values Questionnaire
147. Standardized Questionnaire
148. Structured Interview Matrix Facilitation Technique / Interview Matrix (Sim)
149. Structured Interviews
150. Structured Questionnaire
151. Structured Reflection
152. Students International Physical Activity Questionnaire (IPAQ)
153. Summing Up Rounds
154. Systematic Observation
155. Telephone Interview(S) / Phone Interview
156. Telephone Survey /Computer-Assisted Telephone Interviewing (CATI)
157. The AIR Feedback Model
158. The Circle Of Trust / Trust Circle
159. The Five Whys Method
160. Therapeutic Engagement Questionnaire (TEQ)
161. Thumbs Spectrum
162. Travel Diaries / Travel Journals
163. Unstructured Interviews
164. Usability Questionnaire
165. User Narratives / Life Journey Narratives
166. User Tests
167. Videotaped Observations
168. Videovoice / Videovoice Diaries
169. Virtual Reality Symptoms Questionnaire (VRSQ)
170. Visual Narratives / Visual Storytelling
171. Walk-Along Interviews / Go-Alongs / Walking Interviews
172. We Saw, We Heard
173. Working Alliance Inventory – Short Revised (WAI-SR)
174. Writing A Letter To Yourself

Stimulating = 150 methods

1. "What If" Brainstorming / What If-Ing
2. 3-12-3 Brainstorm
3. 4 Quadrants
4. 5-4-3-2-1 Grounding Technique
5. 6*8*5 Method
6. A Journey Of Discovery
7. Air Time Mastermind
8. Apple-Drawing Ideation
9. Bed Sheet Ping-Pong
10. Bisociation
11. Body Part Twister
12. Bodystorming

13. Brain Writing / Brainwriting / Group Passing / Slip Writing
14. Brainstorming
15. Brainwriting 6-3-5
16. Campfire Method
17. Challenge Prizes
18. Check-In / Check-Out
19. Closing Circle
20. Coach Yourself Out Of A Funk In 3 Steps
21. Connect The Dots
22. Count Up / Count To Ten
23. Cover Story
24. Crazy 8
25. Crazy 8's
26. Crocodile River
27. Current Strongest Impression
28. Dance, Dance, Dance
29. Difficult Co-Creators
30. Dinner Challenge
31. Draw An Apple / Apple-Drawing Ideation
32. Draw Toast
33. Ethos, Logos, Pathos
34. Extreme Characters
35. Feedback: Appreciation Mingle
36. Feedback: I Appreciate…
37. Feedback: Start, Stop, Continue / Start, Stop, Continue
38. Figure Storming
39. Find Your Pair
40. Forced Analogy
41. Future Backcasting
42. Future Wheel
43. Go Bananas
44. Go-Round(S)
45. Graphic Jam
46. Green, Heart, I
47. Habit Reflection: Create A Habit That Sticks
48. Hello Kitty
49. Here, There, Everywhere
50. Hero Stories
51. Hot Air Balloon
52. How-Now-Wow Matrix
53. Human Machine
54. I Like, I Wish, I Wonder / I Like, I Wish, What If
55. I Love You Honey…
56. Ideastorming / Ideastorms
57. Image-Ination
58. Innovation Tournament
59. Inspiration Walk
60. Jam Session / Design Jam / Innovation Jam

61. Kanban Pizza Game
62. Keys Method
63. Knot Game / Untangle Yourself
64. Lego Challenge
65. LEGO Serious Play / LEGO® Serious Play
66. Letter To Myself
67. Line-Up
68. Lo-Tech Social Networking / Low-Tech Social Network
69. Love Your Neighbour?
70. Lower A Bamboo Cane To The Ground
71. Marimba!
72. Marshmallow Challenge
73. Mingle
74. Mirroring
75. Mission Impossible
76. Mood Board(S)
77. My Daily P.A.C.T
78. NUF Test
79. Object Brainstorm
80. Opening Circle
81. Paint A Picture
82. Pair Introductions / Introduction In Pairs
83. Pass A Loop Round The Circle
84. Pass The Squeeze
85. People Bingo
86. Picture Yourself
87. Portrait Drawing
88. Portrait Gallery
89. Post-Up
90. Pronoun Rounds
91. Protobot
92. Prune The Future
93. Prune The Product Tree
94. Question Balloons
95. Questorming
96. Quickly Sketch Noting / Sketchnoting
97. Quiet Brainstorming / Silent Brainstorming
98. Rain Making
99. Random Inputs
100. Reverse Brainstorming / Reverse Thinking
101. Rock Paper Scissor Tournament
102. Role Storming / Rolestorming
103. Role-Playing Game / Role Play(S)
104. Rollercoaster / Rollercoaster Check-In
105. Round Robin / Round Robin Brainstorming
106. Roving Ideastorms
107. Science Fiction Prototyping (SFP)
108. Shake All Hands

109. Shake Down 5-4-3-2-1 / Shake Down
110. Shrinking Islands
111. Skill Share
112. Sky The Limit Brainstorm
113. Squiggle Birds
114. Starbursting
115. Start Small
116. Start Your Day
117. Stinky Fish
118. Stop And Go
119. Story Puzzle
120. Structured Brainstorm
121. Super Hero
122. Sync Claps
123. Synesthesia
124. Talking Chips
125. Talking Stick(S) / Wooden Stick
126. Team Brainstorming
127. The 6 Thinking Hats / Feedback Hats
128. The Anti-Problem
129. The Blind Side
130. The Reverse Perspective
131. The Shouting Game
132. The Viking
133. This N' That
134. Tomorrow's Narratives / Tomorrow's Headline / Advertising Posters / Service Evidencing / Future Newspaper / Newspaper Headlines Of The Future
135. Trading Cards
136. Trigger Storming
137. Trust Falls In Pairs
138. Trust Ladder
139. Two Truths And A Lie
140. Unintended Consequences / Dormant Opportunities
141. Walkabout
142. What Are You Doing?
143. What's In The Bag?
144. Who Are We Now?
145. Who Inspires Us?
146. Who Is It?
147. WhoDo
148. Whole Product Game
149. Wizards, Pixies, Giants / Giants, Wizards, Elves
150. Write A Blurb

Collaborating = 63 methods

1. 1,2,4, All!
2. 5 Bold Steps

3. 90-Minute Prototypes
4. Action Group(S)
5. Align On Your Impact Goals
6. Alignment & Autonomy
7. Argument Map / Argument Mapping
8. Atomize
9. Bundle Ideas
10. Carousel / Carousel Technique
11. Challenge Cards
12. Cogenerative Dialogues (Cogens)
13. Collaboration Analysis/ Collaborative Data Analysis
14. Collaborative Mapping (Aka Citizen Mapping)
15. Collaborative Pilot Schedule
16. Collective Narratives
17. Collective Reflection
18. Collective Vision Building
19. Conflict Responses
20. Customer, Employee, Shareholder
21. Design Critique
22. Design The Box
23. Dialogue Cafés
24. Dialogue Of Wisdom (Wisdom Dialogues)
25. Dilemma Café
26. Do, Redo & Undo
27. Draw The Problem
28. Engineering Your Team OS
29. Fish Bowl / Fishbowl
30. Flower Of Thoughts
31. Four Square Story
32. Friend Or Foe?
33. Future Mapping / Future Map
34. Future Trends
35. Geographical Mapping / GIS
36. Group Level Assessment (GLA)
37. Group Model Building / Group-Model Building / Group Model-Building / Building A Model
38. Grow An Idea
39. Idea & Concept Development
40. Idea Remix
41. Innovation Portfolio Exercise
42. Life Café
43. Lights, Camera, Action!
44. Make A World
45. Mash-Up Innovation / Mash-Ups
46. Mini-Campaign Challenge
47. Nagging
48. Neo-Socratic Dialogue
49. Open Space Technology
50. Participatory Photography (PP)

51. Participatory System Dynamics (SD)
52. Participatory Theatre
53. People Map / People Mapping
54. Post-It Note Race / Sticky Note Race
55. Public Stack Puzzle
56. Quaker Conversation / Quaker Dialogue
57. Scenario Mapping
58. Sketching Group / C-Sketch / Collaborative Sketching / 5-1-3 G
59. The Blue Sky Vision Exercise / Blue Skye Envisioning
60. Video Card Family Game
61. Vision Factory
62. Walt Disney Method
63. World Café

Collective Decision-making = 21 methods

1. 20/20 Vision
2. 3 Action Steps
3. Bang-For-The-Buck
4. Deliberative Dialogue
5. Diamond Ranking / Diamond Ranking Strategy
6. Direct Ranking (Direct Matrix Ranking DMR)
7. Dotmocracy / Dot Voting / Coloured Dot Voting / Prioritisation Dots
8. Electronic Voting
9. Forced Ranking
10. Impact/Effort Matrix / Impact & Effort Matrix
11. Interdisciplinary Work Groups
12. Narrow The Set
13. Near And Dear
14. Non-Structural Fuzzy Decision Support System (NSFDSS)
15. Pair-Wise Ranking
16. Pros & Cons / Pros Cons List
17. Simple Multi-Attribute Rating Technique (SMART)
18. Soft Systems Methodology (SSM)
19. Sorting Important To/For
20. Urgent/Important Grid (Aka Eisenhower Matrix, Or Eisenhower Decision Matrix)
21. Yonmenkaigi System Method (YSM)
